# Altered amygdala and striatal responsivity and prediction error encoding of temporal reward dynamics in post-trauma psychopathology

**DOI:** 10.1038/s41386-025-02276-z

**Published:** 2025-11-17

**Authors:** Lauren K. Enten, Annamarie DeMarco, Rachel Kline, Franziska M. Globisch, Joseph E. Dunsmoor, Josh M. Cisler, Charles B. Nemeroff, Gregory A. Fonzo

**Affiliations:** 1 Department of Psychiatry and Behavioral Sciences, The University of Texas at Austin Dell Medical School, Austin, TX USA; 2 Department of Neuroscience, The University of Texas at Austin, Austin, TX USA

**Keywords:** Reward, Post-traumatic stress disorder

## Abstract

Diminished positive affect is a hallmark of post-trauma psychopathology (PTP), e.g. posttraumatic stress disorder and major depressive disorder, yet the circuit dysfunction underlying these PTP symptoms is poorly understood. Computational models offer a powerful framework to probe learning processes by linking such processes to brain activity in regions involved in updating reward predictions. Here, we examined fMRI neural responsivity to temporal prediction errors—deviations in timing of expected reward delivery—and applied a temporal difference (TD) learning model to characterize model-based prediction error neural encoding in individuals with PTP (*N* = 45; 32 females) and trauma-exposed healthy controls (*N* = 45; 25 females). Participants learned that a visual cue reliably predicted timing of a primary reward (oral juice bolus). To induce temporal prediction errors, we introduced occasional “catch” trials, which unexpectedly extended the cue-outcome interval. Compared to controls, the PTP group showed blunted activation in the left amygdala and putamen to reward receipt at unexpected vs. expected times and blunted deactivation to reward absence at unexpected vs. expected times (corrected *p’s* < 0.05). Interestingly, attenuated amygdala deactivation was associated with lower anhedonia (ρ_43_ = –0.50, *p* < 0.001), suggesting a possible mechanism for preserving hedonic capacity. Model-based fMRI revealed diminished amygdala and putamen prediction error encoding at reward delivery in PTP but exaggerated insula and putamen encoding at cue presentation (corrected *p’s* < 0.05). We provide new evidence of disrupted PTP amygdala-striatal reward responsivity and computational learning signals, demonstrating an unrecognized pervasiveness of dysfunction extending to the fundamental learning domain of cue-reward timing.

## Introduction

Nearly 80% of individuals will experience a traumatic event [1]. A subset will develop a post-trauma psychopathology (PTP) such as posttraumatic stress disorder (PTSD) and/or major depressive disorder (MDD) [2–4]. PTP is characterized by diminished positive affect [5–7], i.e., reduced frequency/intensity of positive emotions [8]. Diminished positive affect includes anhedonia in MDD and “emotional numbing” in PTSD, i.e., diminished positive emotion, diminished interest, and feeling detached/estranged from others [6]. Recent factor analyses separate emotional numbing symptoms into a distinct factor of post-traumatic anhedonia [9, 10], uniquely predictive of functional impairment [11]. In trauma survivors, PTSD diagnosis and symptom severity also account for variance in diminished positive affect symptoms above and beyond comorbid MDD [12], suggesting such symptoms are a core part of the PTSD diagnosis and not simply an artifact of comorbidity. Although prevalent following trauma [13], the biological and behavioral bases of post-trauma diminished positive affect symptoms are understudied [7, 14]. This is an important area for focus, as diminished positive affect symptoms predict: (a) greater distress and chronicity [15, 16]; (b) greater functional impairment [17, 18]; (c) poorer treatment outcomes [19]; and (d) increased suicidality [20].

Reward processing, i.e., behavior in response to or in pursuit of a reward [21], reliably recruits neural circuits implicated in positive affect [22]. This includes the so-called “reward circuit,” consisting of ventral anterior cingulate cortex (ACC) and orbitofrontal cortex (OFC); striatum; ventral pallidum; and midbrain nuclei including ventral tegmental area (VTA), and substantia nigra [23]. Additional circuitry is also implicated in reward processing through mediation of related affective functions, including dorsal ACC and dorsomedial/dorsolateral prefrontal cortex (PFC), amygdala, insula, hypothalamus, and lateral habenula [23]. Together, core and extended reward circuitry mediates an individual’s reward seeking behavior and clinically relevant constructs such as positive mood/affect [24–26].

Prior studies have examined reward processing circuitry/behavior in PTSD using functional magnetic resonance imaging (fMRI) [7] employing operant decision-making or passive observation paradigms with monetary rewards and losses [27–37]. These studies have revealed suboptimal reward processing behavior and altered responsivity of the reward circuit [7, 38], also linked to severity of diminished positive affect symptoms [28, 31]. Reinforcement learning (RL) models, which estimate and dissect latent components of information processing, have also been examined in these paradigms and in relation to reward circuit function. Evidence indicates diminished neural encoding of both expected value (EV; representation of future reward in response to a predictive cue) and prediction errors (PEs; differences in rewards expected vs. received) in nodes of the core and extended reward circuit in PTSD [31, 32].

However, prior studies have used abstract or secondary generalized reinforcers as probes of reward processing in PTSD, such as points [27, 36, 37], monetary rewards [28, 29, 31, 33, 39–41], or generic visual/social cues, e.g., smiling/attractive faces [42–44] or positive visual images [45]. Evidence indicates there are discernible differences in neural responses to primary vs. secondary rewards [46, 47]. While secondary rewards recruit a greater extent of ventral PFC regions, consistent with abstract representations of reward value, primary rewards (intrinsically rewarding stimuli) show preferential recruitment of evolutionarily primitive brain structures involved in salience detection and interoception (e.g., amygdala and insula) [46]. Thus, use of primary reinforcers (such as food/liquid rewards) in PTP may yield fuller insight into the nature of reward circuit dysfunction [7].

Rewards are also processed by several facets, including type, magnitude, and timing. Though the former are more extensively studied in PTSD, the latter (temporal relationships between reward cues and outcomes) are not well explored. Such temporal relationships have been extensively studied with Pavlovian (associative) reward conditioning paradigms, where a cue is learned to predict delivery of a subsequent outcome at a learned temporal interval [48]. Learning is thought to be mediated by dopamine signaling, particularly in the mesolimbic pathway [49], and is explained algorithmically by temporal difference (TD) learning, which effectively models dopamine neuron signaling dynamics [50]. TD learning, unlike traditional RL, considers timing within a trial [50]. This model captures how midbrain dopamine neuron firing occurs initially at the time of reward delivery (initial PEs generated) and then gradually backpropagates to the predictive cue. In this context, dopamine neurons are thought to encode signed PEs, increasing firing for positive PEs and decreasing firing for negative PEs. Here, we use the term “temporal PEs” to refer to violations in temporal expectations between cue and reward, while the term “TD learning PEs” refers to computational model numerical PE estimates.

Although the neural correlates of traditional PEs (deviations in magnitude or type of reward) have been extensively studied in healthy individuals [51–53] and to a lesser extent in both PTSD [32] and MDD [54, 55], the neural signaling of temporal PEs is less well investigated. A prior study [56] sought to identify functional magnetic resonance imaging (fMRI) neural correlates of temporal PEs in humans through violating expectancies for cue-reward timing and observed that the left putamen appears to signal a signed temporal PE. That is, it displayed greater activation for reward delivery at unexpected vs. expected times, i.e., positive temporal PE, and decreased activation for reward absence at unexpected vs. expected times, i.e., negative temporal PE. A different study [48] demonstrated that TD learning PEs to both a reward cue and outcome are encoded in blood oxygenation-level dependent (BOLD) fMRI signal in the extended reward circuit, including striatum, orbitofrontal cortex (OFC), and dorsal PFC.

Here, to expand knowledge of PTP-related reward dysfunction, we investigated neural processing of temporal reward cue-outcome relationships using a primary reward stimulus (oral juice bolus). This facilitates detection of abnormalities related to temporal PEs as well as expectation/receipt of primary rewards, neither of which has been examined in PTP. We adopted transdiagnostic inclusion criteria, as PTSD and MDD are both frequent trauma sequalae [2] and both manifest diminished positive affect. To ensure a current clinically significant level of diminished positive affect symptoms in the PTP group, we enforced a minimal cutoff on a common measure of anhedonia as an inclusion criterion while also allowing the groups to differ on other metrics of symptomatology. In addition, we required onset of symptoms directly following (within 1 month) a Criterion A traumatic event to capture trauma-related symptoms. We acquired fMRI during a passive reward learning task previously used in healthy individuals [56] and those with substance use disorders [57, 58] to map neural signaling of violations in cue-reward timing. While undergoing fMRI in a thirsty state, individuals saw two visual cues and were told that one reliably predicted a subsequent oral juice bolus while the other predicted a subsequent neutral visual cue (non-reward control), both after a consistent temporal delay. After learning temporal relationships, “catch” trials for both trial types occasionally extended the delay between cue and outcome. This allowed us to probe brain signaling of reward cues, reward presence and absence at expected trial times, and reward presence and absence at unexpected time points in the trial (temporal PEs). We began with a traditional mass univariate fMRI “activation” analysis isolating, via contrasts, effects of interest (reward vs. non-reward cues, outcomes, and positive and negative temporal PEs). We complemented this with model-based fMRI employing a TD learning model, informed by task structure/timing, which provided continuous TD learning PE estimates. TD learning PEs at cue presentations and outcome deliveries or absences at expected and unexpected times (where TD learning PEs are maximal) were examined as two separate parametric modulators of BOLD signal [59]. Finally, we assessed relationships of brain abnormalities to diminished positive affect symptoms in the PTP group. Given prior evidence [56, 60], we hypothesized the task would elicit signed temporal PE signals, i.e., greater activation for positive temporal PEs and less activation for negative temporal PEs, in left putamen and other areas of the striatum. We also predicted juice TD learning PEs would be encoded by the striatum, amygdala, ACC, and insula. Given attenuated reward circuit activation in PTSD to reward predictive cues and outcomes [7, 38, 61], we hypothesized the PTP vs. trauma-exposed healthy comparison (TEHC) group, would display, in the traditional analysis, attenuated activation in core/extended reward circuitry to juice vs. non-juice cue and outcome. Given evidence for attenuated traditional PE signaling in PTSD [32], we expected attenuated temporal PE BOLD signal changes in the traditional analysis and attenuated model-based neural encoding of TD learning PEs for the PTP vs. TEHC group in reward-responsive areas. Finally, we expected a subset of abnormalities to be associated with diminished positive affect severity in PTP.

## Materials and methods

The study was reviewed and approved by the University of Texas at Austin Institutional Review Board. All procedures were conducted in accordance with board and protocol guidelines.

### Participants/screening

Participants were recruited through online and print advertisements, in-person events, and word-of-mouth from residents of the greater Austin, Texas community. Main inclusion criteria were unmedicated, right-handed, non-nicotine using adults, ages 21 to 50 years, reporting a prior history of one or more Criterion A traumatic events on the Life Events Checklist [62] and eligible to undergo MRI. See Supplementary Methods for exclusion criteria and screening procedures. Two groups of trauma-exposed participants were targeted. The first was a PTP group, with the inclusion criteria of a Mood and Anxiety Symptom Questionnaire (MASQ) [63] Anhedonic Depression (MASQ-AD) subscale score ≥34 (at screening) with current chronic (≥3 months duration) and impairing/distressing symptom onset directly following (within 1 month) a Criterion A traumatic event (verified at clinical interview). The second was a TEHC group reporting exposure to 1 or more Criterion A events and, on structured clinical interview, to not currently nor ever have met criteria for PTSD or another mental health diagnosis. The MASQ-AD cutoff, derived from a sample of individuals with a PTSD diagnosis [64–67] vs. TEHC comparison participants [68], was found to provide good balance of sensitivity/specificity (AUC = 0.90) in distinguishing the PTSD and TEHC groups and also selected for a clinically significant level of diminished positive affect.

### Consent and clinical assessment

Participants provided written informed consent and completed a baseline clinical assessment administered by trained study personnel. The Clinician-Administered PTSD Scale for DSM-5 (CAPS-5) [69] was used to identify presence/absence of a PTSD diagnosis. The Structured Clinical Interview for DSM-5 Diagnosis (SCID-5) [70] was used to assess mood, anxiety, alcohol/substance use, and psychotic disorders. Those initially identified for inclusion in the PTP group were verified during clinical interview to have experienced onset of chronic (lasting ≥3 months), impairing/distressing diminished positive affect symptoms directly following (onset within 1 month) a Criterion A traumatic event. Those in the TEHC group had to have experienced 1 or more Criterion A events but could not currently nor ever have met diagnostic criteria for PTSD nor any other mental health condition. Individuals also completed the clinician-administered Snaith Hamilton Pleasure Scale (SHAPS-C) [71], a clinician-administered form of the widely-utilized self-report measure.

### Self-report measures

In addition to the Life Events Checklist and MASQ completed at screening and the CAPS-5, SCID-5, and SHAPS-C completed at baseline consent/interview, participants completed additional self-report measures after the clinical interview. These included the WHO Quality of Life Inventory BREF (WHOQOL) [72], Beck Depression Inventory II (BDI-II) [73], Positive and Negative Affect Schedule (PANAS) [74], Temporal Experiences of Pleasure Scale (TEPS) [75], and the short form of the Childhood Trauma Questionnaire [76].

### fMRI acquisition

On a separate day, individuals returned to undergo brain imaging at The University of Texas (UT) Biomedical Imaging Center (BIC) on a 3.0 T Siemens MAGNETOM Vida using a 64-channel head/neck coil. Individuals were instructed to not drink any liquids for 3 h prior to the visit to increase rewarding properties of juice delivery during scanning. Participants each provided their preferred juice type during the baseline clinical interview. See Supplementary Methods for details on MRI acquisition parameters and juice delivery procedure.

### Passive reward learning task

**Fig. 1:**
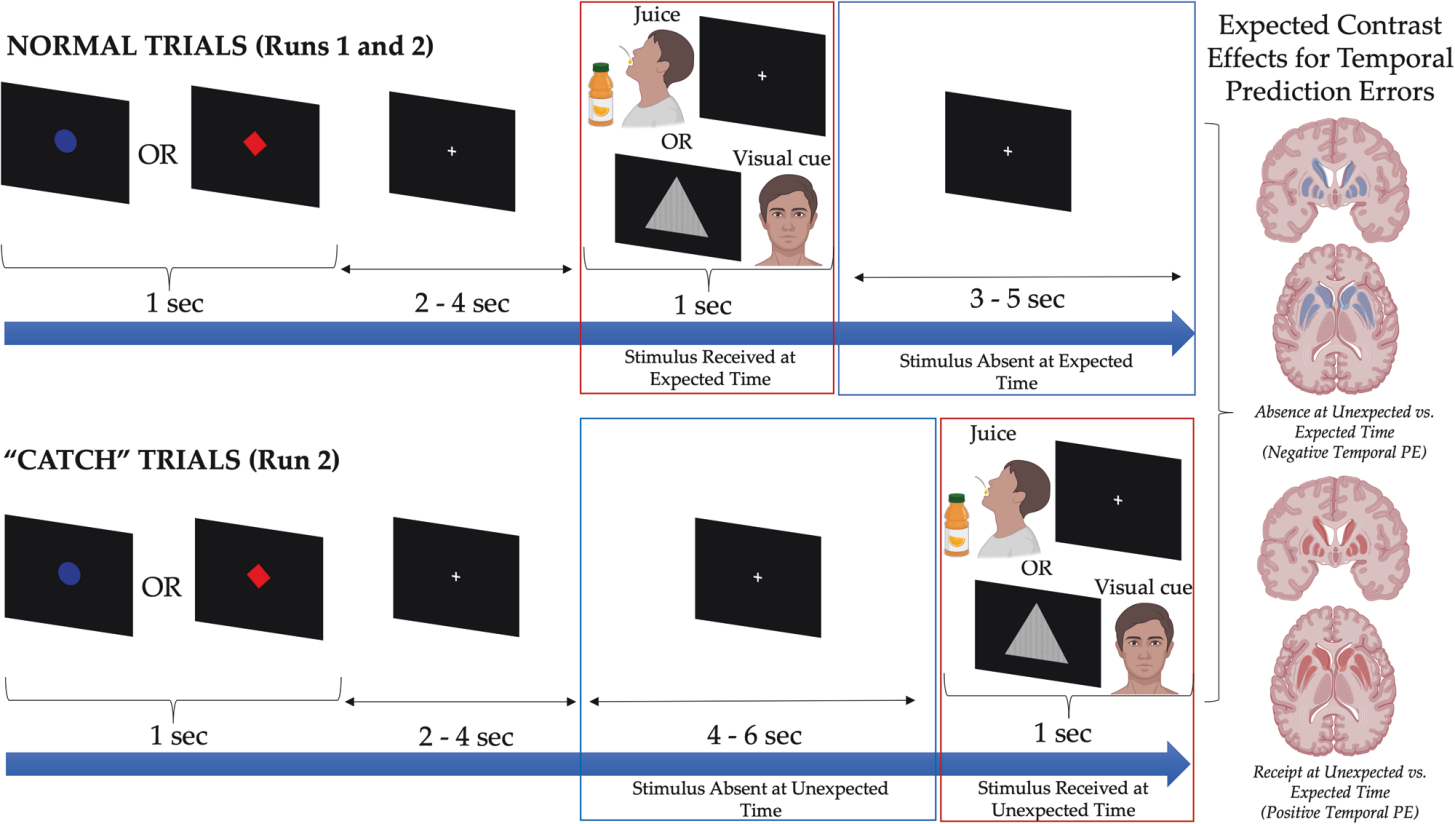
Passive reward learning task design and induction of temporal prediction errors. Figure depicts the two types of trials on the passive reward learning task. The top row depicts the timing/structure of normal trials, in which juice or visual cue delivery follows a reliable temporal delay after the predictive cue. The bottom row depicts the “catch” trials, in which the temporal delay between the cue and predictor is unexpectedly extended. This results in four distinct temporal windows across both trial types. The mapping of activation for positive temporal prediction errors (PEs) for each trial type (juice and visual cue) occurs through contrasting activation for stimulus receipt at unexpected vs. expected times (outlined in red boxes), controlling for receipt of the stimulus with the difference being the expectation for stimulus receipt at that time. The mapping of activation for negative temporal PEs for each trial type (juice and visual cue) occurs through contrasting activation for stimulus absence at unexpected vs. expected times (outlined in blue boxes), controlling for absence of the stimulus with the difference being the expectation for stimulus absence at that time. The column on the far right depicts a priori hypothesized effects of the temporal PEs on striatal activation, with positive temporal PEs expected to induce striatal activation (depicted as brains with red shading on striatal structures) and negative temporal PEs expected to induce striatal deactivation (depicted as brains with blue shading on striatal structures).

Task was programmed and presented in PsychoPy version 3.0.1 [77]. See Fig. 1 for a schematic and Supplement for complete details. The first task run encompassed 54 trials of cue-outcome learning, 27 of each condition, all temporally consistent with cues preceding outcomes by 2–4 s. The second task run encompassed 72 trials: 26 temporally consistent trials of each type (juice and visual cue outcome) and 10 “catch” trials of each type, where delay between cue and outcome was extended to 8–10 s. “Catch” trials were placed in the latter 75% of trials on the second run to maximize learning of the typical cue-outcome temporal delay in run 1 and the first part of run 2 prior to violating temporal relationships, which we theorized would maximize temporal PEs. A catch trial of each type (juice or visual cue) was always followed by one or more “normal” trials of the same type prior to the next catch trial to re-instate temporal expectation and maximize prediction errors. Stimulus perceptual modality (gustatory vs. visual) differed for the two task conditions, which is not ideal for isolating reward-specific effects. This design choice was necessitated by equipment and logistical constraints and was intended to provide a minimal perceptual control condition to establish specificity of oral juice bolus-related effects. Effects therefore reflect a mix of both reward-related and perceptual modality-related differences.

### Visual analog scales

Participants completed written visual analog scales after task completion rating the degree to which they were thirsty prior to the scan; how much they enjoyed juice delivery; and how well they could taste the juice (see Supplementary Methods for details).

### fMRI preprocessing and individual-level fMRI activation analysis

Preprocessing was implemented in FSL [78]. See Supplementary Methods for details. Individual-level analyses were conducted in AFNI [79]. Separate regressors for juice and non-juice trials modeling onset and duration of several events within each trial were included (all juice and non-juice events were modeled as separate regressors). First, we modeled predictive cues (1 s duration) for juice and non-juice trials. Second, we modeled temporally predicted outcomes for juice and non-juice trials (1 s duration, with juice bolus onset shifted an additional 0.5 s from trigger related to compliance in plastic tubing; delay/duration of flow determined through empirical testing). Third, we modeled expected absence of juice and non-juice outcomes (onset 2 s following outcome delivery with 1 s duration). Fourth, we modeled unexpected absence of juice and non-juice outcomes (4 s following offset of cue on catch trials, i.e. very end of typical temporal window for outcome delivery, with 1 s duration and an additional 0.5 s onset delay for juice trials). Fifth, we modeled unexpected receipt of juice and non-juice outcomes (time of outcome delivery on catch trials, with additional 0.5 s delay for juice bolus delivery and 1 s duration). From these events, we calculated several within-subject contrasts of interest. First, we contrasted juice vs. non-juice cues. Second, we contrasted juice vs. visual cue outcome for normal trials. Third, for both juice and non-juice trials, we calculated separate contrasts capturing positive temporal PEs, i.e., unexpected outcome receipt on catch trials vs. expected outcome receipt on normal trials. Fourth, for both juice and non-juice trials, we calculated separate contrasts capturing negative temporal PEs, i.e., unexpected outcome absence on catch trials vs. expected outcome absence on normal trials. Fifth, we calculated the contrast of juice vs. non-juice positive temporal PEs. Sixth, we calculated the contrast of juice vs. non-juice negative temporal PEs. As in prior work [56], unexpected vs. expected receipt or absence of juice delivery contrasts were used to estimate positive and negative temporal PEs, respectively. Here, we contrasted these between juice and non-juice trials to better isolate brain activity specific to reward-related temporal PEs. See Supplementary Methods for full details.

### TD learning model

The TD learning model was initially proposed by Schultz et al. [50] and utilized by O’Doherty et al. [48] to examine fMRI BOLD correlates of TD learning PEs. Following a cue, TD learning attempts to predict for each time, *t*, within the subsequent trial the total future reward to be gained in the trial from time *t* to the end of the trial. This is optimized through moment-by-moment TD learning PEs, i.e. difference between the sum of current (realized) rewards and expected future value at time *t* + 1 (weighted by a temporal discounting factor) from that expected at time *t*. TD learning PEs are used to update EV at each time *t* in subsequent trials (weighted by a learning rate), with perfectly predicted sequences generating no PEs. The model and underlying theory is explained in the Supplementary Methods. See Supplementary Fig. 1 for a visual depiction of TD learning. This model produced a TD learning PE value at each time step of the task, which was then used to examine fMRI BOLD signal encoding of TD learning PE signaling.

### Model-based fMRI individual analysis

TD learning PE values at specific time steps were examined as parametric modulators of BOLD signal for cue and outcome for juice and visual cue conditions separately (4 parametric regressors in total). For cues, we modeled TD learning PEs at each cue onset (which carry information about upcoming stimulus delivery and thus engender positive TD learning PEs). For outcomes, we modeled activity at times of expected and unexpected outcome delivery (juice or visual cue), each parametrically modulated by the TD learning PE value at that time step as predicted by the model (where TD learning PEs are maximally positive). To capture negative TD learning PE signaling in each outcome regressor, we also modeled activity for stimulus absence at unexpected times (where TD learning PEs are maximally negative due to absence of outcome delivery at points of high expected value). Although the model provides a TD learning PE estimate at every time step, for parametric modulation we sampled the TD learning PEs at these specific trial time points since they capture the largest magnitude and most informative TD learning PE signals [48]. Additional non-modulated main effect regressors were also included. Individual analysis was conducted as in the model-free analysis (i.e., motion regressors, etc.). Outcomes of interest were individual beta weight maps for parametric modulation of BOLD signal as a function of TD learning PEs for both cues and outcomes [48]. As constraining interpretation to differences in degree of parametric modulation of BOLD signal for one condition (e.g., juice trials) vs. another (non-juice trials) may limit detection power and is not a common practice in model-based fMRI, we examined TD learning PE encoding in juice and non-juice trials separately.

### fMRI group analysis

Task effects for contrasts of interest were compared against 0 using a one-sample t-test in the AFNI program *3dttest* + *+*. Group differences were examined using an independent t-test with *3dttest* + *+*. Contrasts of interest in the traditional analysis were reward vs. non-reward cues, juice vs. visual cue outcomes, juice vs. visual cue positive temporal PEs, and juice vs. visual cue negative temporal PEs. Effects of interest in the model-based analysis were BOLD signal encoding of TD learning PEs to: (a) juice cue presentation; and (b) expected and unexpected juice deliveries and absences. T-statistic maps were then converted to Z scores and subjected to probabilistic threshold free cluster enhancement [80] (pTFCE) for Type I error correction, which utilized residual maps to characterize smoothness of statistical maps for analysis. This was conducted within a region of interest (ROI) mask encompassing core and extended reward-responsive regions, including (all bilaterally) amygdala, caudate, putamen, globus pallidus, nucleus accumbens, midbrain structures (substantia nigra, red nuclei, subthalamic nuclei, ventral tegmental area (VTA), parabrachial pigmented nuclei, hypothalamus, mammillary nuclei), insula, and ventromedial prefrontal regions (see Supplementary Methods for details). A whole-brain analysis was also conducted with pTFCE correction for multiple comparisons. This study was designed to be sufficiently powered at *N* = 90 to detect group differences of a moderate effect size at $\alpha$ of 0.05. However, given the multiplicity of task contrasts examined here, we report the significance of group differences using two complementary criteria. Our a priori primary planned threshold, matching planned power, is a more lenient contrast-wise alpha (${\alpha }_{{CW}}$) of 0.05, in which Type I error was controlled at the level of a single contrast. This criterion was used for deriving task effect maps and for initial examination of group differences. This criterion maximizes statistical detection power but also results in a higher potential false positive rate across all contrasts examined. The second criterion is a more stringent experiment-wise alpha (${\alpha }_{{EW}}$) of 0.05, which provides more limited detection power but maintains the false positive tolerance level across all fMRI comparisons. For this latter correction, we adopted the Bonferroni correction method. We examined six contrasts from the fMRI task for group differences (across both traditional and model-based analyses), resulting in a corrected ${\alpha }_{{CW}}$ = 0.05/6 = 0.008333. Thus, group differences which survived a pTFCE-corrected p_CW_ < 0.008333 would be considered to meet the criterion to maintain the ${\alpha }_{{EW}}$ at 0.05, i.e. pTFCE-corrected p_EW_ < 0.05. We hereafter refer to these effects as pTFCE-corrected p_CW_ < 0.05 (the more lenient threshold maintaining ${\alpha }_{{CW}}$ at 0.05) and pTFCE-corrected p_EW_ < 0.05 (the more stringent threshold maintaining ${\alpha }_{{EW}}$ at 0.05).

### Brain-symptom relationships

To evaluate the nature of detected brain abnormalities, we examined correlations between juice trial activation metrics in the PTP group with symptom measures, including the SHAPS-C, CAPS emotional numbing/anhedonia items [9, 10], and TEPS anticipatory (TEPS-A) and consummatory (TEPS-C) pleasure facets. We corrected for multiple comparisons across separate measures using Bonferroni correction for the number of measures (4 in total) and number of abnormalities within a family of analyses (traditional univariate and model-based analyses considered separate families), yielding a corrected *p* < 0.0025. We also verified correlations remained when contrasting juice vs. non-juice trial metrics.

## Results

### Participants

PTP and TEHC groups did not significantly differ on age (PTP: 27.91 ± 6.05; TEHC: 26.18 ± 6.78), gender, years of education (PTP: 16.01 ± 2.63; TEHC: 16.71 ± 2.36), and IQ (PTP: 118.61 ± 12.71; TEHC: 118 ± 12.96)(Supplementary Table 1), though there were a greater proportion of females in the PTP (*N* = 32; 71% of sample) vs. the TEHC group (N = 25; 56% of sample). Ethnicity and race were evenly distributed across groups, and most participants were of non-Hispanic ethnicity (~70%) and identified as White (~60%) or Asian (~24%). The PTP group reported significantly greater personal experience of physical assault, sexual assault, and other unwanted sexual experiences (*p*’s ≤ 0.001) on the Life Events Checklist and significantly greater childhood maltreatment across all domains (*p’s* < 0.01) on the Childhood Trauma Questionnaire. The two groups also differed in chronicity of symptoms (*p* < 0.001). In the PTP group, the mean duration of symptoms was ~6 years. The TEHC group, consistent with the lower levels of symptomatology enforced by the inclusion criterion (never meeting criteria for PTSD), had much shorter duration of symptoms (mean of 1.44 months). In the PTP group, all 45 individuals met criteria for current PTSD, 21 for current MDD, 11 for current generalized anxiety disorder, and 2 for current panic disorder. PTSD severity in the PTP group was, on average, in the moderate-to-severe range on CAPS-5 (mean score of ~36), while depression symptoms on the BDI-II were in the moderate range (mean of ~22).

### Visual analog scales

See Supplementary Results for details. The PTP vs. TEHC group displayed less enjoyment of the juice (*F*_*1,85*_ = 4.07, *p* = 0.047) but did not differ on capacity for taste or degree of thirst (*p*’s > 0.36).

### Traditional fMRI activation

Here, we report on findings in the ROI-constrained analyses (all pTFCE-corrected *p*_*CW*_ < 0.05). See Supplementary Results for results of whole-brain exploratory analyses.

#### Juice vs. non-juice cue

This contrast produced lower juice vs. non-juice cue activation in the bilateral amygdala, midbrain, striatum, and mid/posterior insula, with no areas showing greater activation (Supplementary Fig. 2A and Supplementary Table 2). *T* test maps for juice (Supplementary Fig. 2B) and non-juice (Supplementary Fig. 2C) cues separately revealed prominent activation for both conditions, but activation magnitudes were larger for the non-juice cue. There were no significant group differences.

#### Juice vs. visual cue delivery at expected times

This contrast produced greater juice vs. visual cue receipt activation in bilateral midbrain, amygdala, ventral striatum, pallidum, putamen, and anterior/mid-insula (Supplementary Fig. 3A and Supplementary Table 3). Greater activation for visual cue vs. juice receipt was observed in bilateral caudate head/body/tail, left perigenual ACC (pgACC), and right posterior insula. There were no group differences.

#### Juice vs. visual cue positive temporal PEs

**Fig. 2:**
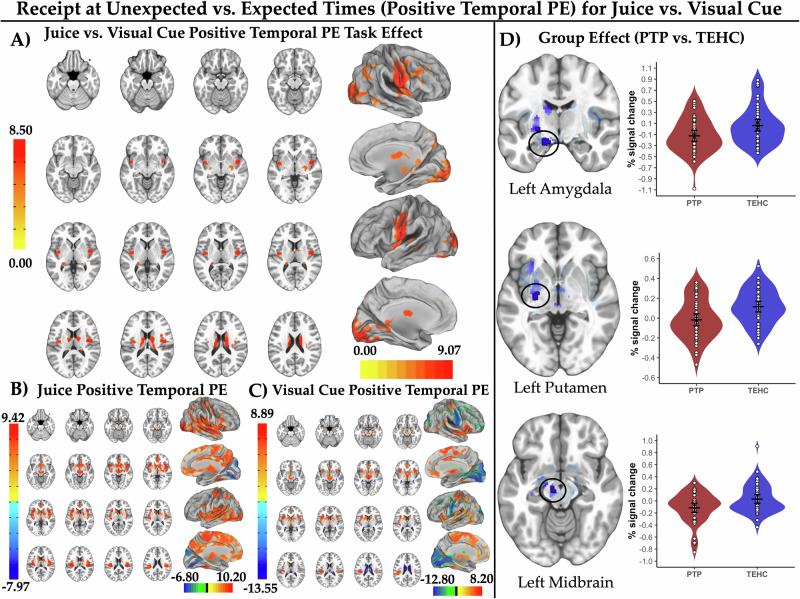
Activation and post-trauma psychopathology abnormalities for juice vs. visual cue positive temporal prediction errors. **A** depicts the reward region of interest (ROI)-constrained patterns of activation for juice vs. visual cue positive temporal prediction errors (PEs) (brain slice montage displayed on the MNI152 ICBM 2009c non-linear asymmetric average brain) as well as patterns of positive temporal PE activation in the whole brain exploratory analysis (projected onto an average brain surface for visual display). **B**, **C** depict the ROI-constrained patterns and whole brain activation patterns for each of the juice (2B) and visual cue conditions (2 C) separately, respectively. Color bars indicate the magnitude of the probabilistic threshold-free cluster enhancement (pTFCE)-corrected signed Z values (with positive indicating activation and negative indicating deactivation) displayed on the brain images. **D** depicts areas of attenuated juice vs. visual cue positive temporal PE activation identified in the ROI-constrained analysis at a pTFCE-corrected p_CW_ < 0.05 for the post-trauma psychopathology (PTP) vs. trauma-exposed healthy comparison group. Suprathreshold voxels are outlined in black with a linear fade applied to subthreshold voxels as a function of distance from significance threshold. Results are displayed on the MNI152 ICBM 2009c non-linear asymmetric average brain. Violin plots depict average fMRI % signal changes for the juice vs. visual cue positive temporal PE contrast for each participant averaged over suprathreshold voxels. White dots indicate observed mean values for each participant averaged over suprathreshold voxels. Widest black horizontal line within each group violin plot indicates the mean value, with the black lines above/below indicating the 95% confidence interval for the group mean.

As hypothesized, this contrast produced larger juice vs. visual cue positive temporal PE activation in the bilateral posterior putamen/pallidum, left midbrain, bilateral mid-insula, and bilateral caudate body/tail (Fig. 2A and Supplementary Table 4). Examination of juice (Fig. 2B) and visual cue (Fig. 2C) positive temporal PE task effect maps separately revealed prominent juice positive temporal PE activation, i.e., greater activation for juice receipt at unexpected vs. expected times. As hypothesized, the PTP vs. TEHC group displayed attenuated juice vs. visual cue positive temporal PE activation in the left midbrain, left amygdala, and left posterior putamen/pallidum (pTFCE-corrected p_CW_ < 0.05; Fig. 2D and Supplementary Table 5). At the more stringent pTFCE-corrected p_EW_ < 0.05, the effect in the left putamen survived, but effects in the left midbrain and left amygdala did not.

#### Juice vs. visual cue negative temporal PEs

**Fig. 3:**
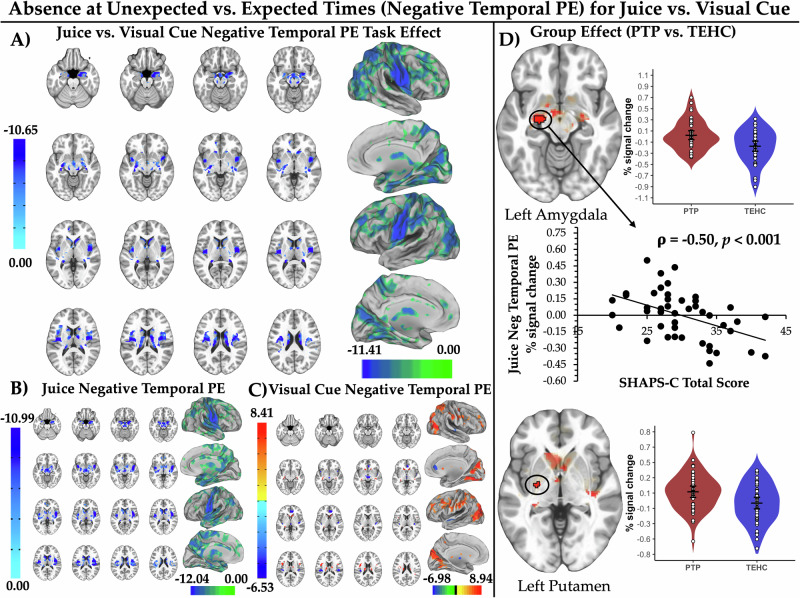
Deactivation and post-trauma psychopathology abnormalities for juice vs. visual cue negative temporal prediction errors. **A** depicts the reward region of interest (ROI)-constrained patterns of deactivation for juice vs. visual cue negative temporal prediction errors (PEs) (brain slice montage displayed on the MNI152 ICBM 2009c non-linear asymmetric average brain) as well as patterns of negative temporal PE activation in the whole brain exploratory analysis (projected onto an average brain surface for visual display). **B**, **C** depict the ROI-constrained patterns and whole brain activation patterns for each of the juice (3B) and visual cue conditions (3 C) separately, respectively. Color bars indicate the magnitude of the probabilistic threshold-free cluster enhancement (pTFCE)-corrected signed Z values (with positive indicating activation and negative indicating deactivation) displayed on the brain images. **D** depicts areas of attenuated juice vs. visual cue negative temporal PE deactivation identified in the ROI-constrained analysis at a pTFCE-corrected p_CW_ < 0.05 for the post-trauma psychopathology (PTP) vs. trauma-exposed healthy comparison (TEHC) group. Scatterplot depicts the significant negative relationship between left amygdala negative temporal PE signaling in the PTP group with scores on the clinician-administered Snaith Hamilton Pleasure Scale (SHAPS-C). Suprathreshold voxels are outlined in black with a linear fade applied to subthreshold voxels as a function of distance from significance threshold. Results are displayed on the MNI152 ICBM 2009c non-linear asymmetric average brain. Violin plots depict average fMRI % signal changes for the juice vs. visual cue negative temporal PE contrast for each participant averaged over suprathreshold voxels. White dots indicate observed mean values for each participant averaged over suprathreshold voxels. Widest black horizontal line within each group violin plot indicates the mean value, with the black lines above/below indicating the 95% confidence interval for the group mean.

This contrast produced, as hypothesized, greater deactivation for juice vs. visual cue negative temporal PEs (or greater activation for visual cue vs. juice negative temporal PEs) in bilateral midbrain, amygdala, putamen/pallidum, mid-insula, caudate head/body/tail, and left perigenual anterior cingulate cortex (pgACC) (Fig. 3A and Supplementary Table 6). Examination of juice (Fig. 3B) and visual cue (Fig. 3C) task effect maps separately revealed juice condition effects were all negative, i.e., deactivation for stimulus absence at unexpected vs. expected times (or activation for stimulus absence at expected vs. unexpected times). As predicted, the PTP vs. TEHC group displayed attenuated juice vs. visual cue negative temporal PE deactivation (or attenuated visual cue vs. juice negative temporal PE activation) of the left amygdala and left posterior putamen/pallidum (pTFCE-corrected p_CW_ < 0.05; Fig. 3D and Supplementary Table 7). Both effects also survived the more stringent statistical correction (pTFCE-corrected p_EW_ < 0.05).

### Abnormal brain activation-symptom relationships

Greater PTP failure to deactivate the left amygdala to negative temporal PEs in the juice condition was associated with lower scores on the SHAPS-C (ρ_43_ = –0.50, *p* < 0.001), i.e., less deactivation was associated with more intact hedonic capacity (Fig. 3D). This correlation remained significant when contrasting juice vs. visual cue negative temporal PEs (ρ_43_ = –0.30, *p* = 0.045). To better characterize this finding, we explored, in the PTP group, other areas of response to juice vs. visual cue negative temporal PE contrast associated with SHAPS-C total scores using a whole brain linear regression implemented in AFNI with 3dRegAna. The only region to survive statistical correction within the ROI-constrained mask (pTFCE *p* < 0.05) was part of the left midbrain (encompassing the VTA by CIT168 RL atlas [81] definitions), in which greater activation/less deactivation was associated with lower SHAPS-C total scores (more intact hedonic capacity) (Supplementary Fig. 4). This relationship arose from juice negative temporal PEs (*t*_*43*_ = –2.17, *p* = 0.036) but was not present in visual cue negative temporal PEs (*t*_*43*_ = 0.40, *p* = 0.69).

### Model-based fMRI encoding of TD learning PEs

**Fig. 4:**
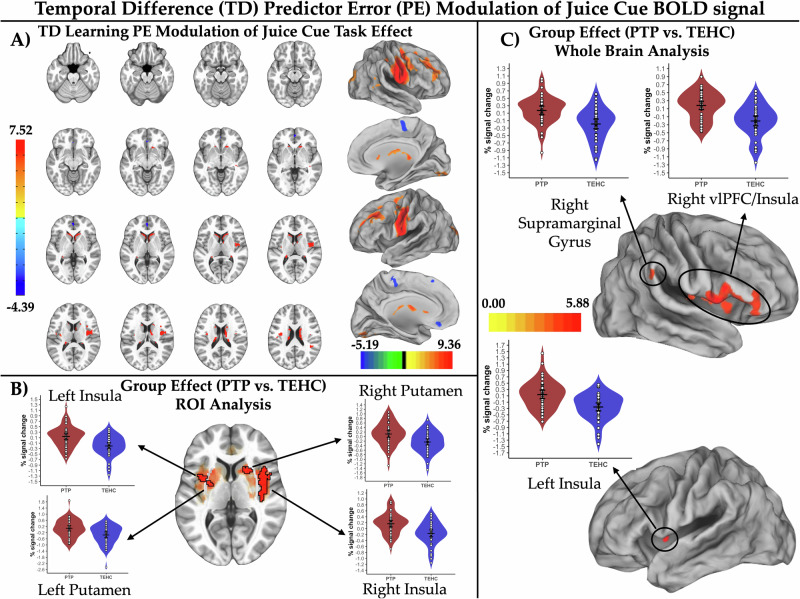
Temporal difference prediction error modulation of juice predictive cue activation and post-trauma psychopathology abnormalities. **A** depicts the reward region of interest (ROI)-constrained patterns of activation modulation by temporal difference (TD) learning model prediction errors (PEs) for blood oxygenation level dependent (BOLD) responses to the juice-predictive visual cue (brain slice montage displayed on the MNI152 ICBM 2009c non-linear asymmetric average brain) as well as patterns of TD learning PE modulation of BOLD response to the juice-predictive visual cue in the whole brain exploratory analysis (projected onto an average brain surface). **B** depicts abnormalities in TD learning PE modulation of juice predictive-cue BOLD signal for the post-trauma psychopathology (PTP) vs. trauma-exposed healthy comparison (TEHC) group identified in the ROI analysis at a pTFCE-corrected p_CW_ < 0.05. Results are displayed on the MNI152 ICBM 2009c non-linear asymmetric average brain. Suprathreshold voxels are outlined in black with a linear fade applied to subthreshold voxels as a function of distance from significance threshold. **C** depicts abnormalities in TD learning PE modulation of juice predictive-cue BOLD signal for the PTP vs. TEHC group identified in the whole brain analysis at a pTFCE-corrected p_CW_ < 0.05. Results are projected onto an average brain surface for visual display. For both 4B and 4 C, violin plots depict average fMRI % signal changes for TD learning PE modulation of activation to the juice-predictive cue for each participant averaged over suprathreshold voxels. White dots indicate observed mean values for each participant averaged over suprathreshold voxels. Widest black horizontal line within each group violin plot indicates the mean value, with the black lines above/below indicating the 95% confidence interval for the group mean.

We report, below, findings in the ROI-constrained analyses for TD learning PE modulation of BOLD signal during juice trials (all pTFCE-corrected *p*_*CW*_ < 0.05). See Supplementary Results for findings of whole-brain analyses (Fig. 4C) and for findings from ROI-constrained analyses of visual cue comparator trials (Supplementary Fig. 5 and 6, Supplementary Tables 12 and 13).

#### TD learning PE modulation of juice cue BOLD signal

We observed positive modulation of BOLD signal by TD learning PEs in the bilateral caudate head/body/tail and posterior insula (Fig. 4A and Supplementary Table 8). Negative modulation was observed in the left pgACC and ventral ACC. Contrary to expectations, the PTP vs. TEHC group showed exaggerated juice cue TD learning PE modulation in the bilateral anterior/mid-insula and putamen (pTFCE-corrected p_CW_ < 0.05; Fig. 4B), which was partially replicated in the whole brain analysis (pTFCE-corrected p_CW_ < 0.05; Fig. 4C). Effects in both the ROI and whole-brain analysis also survived the more stringent pTFCE-corrected p_EW_ < 0.05 criterion.

#### TD learning PE modulation of juice delivery BOLD signal

**Fig. 5:**
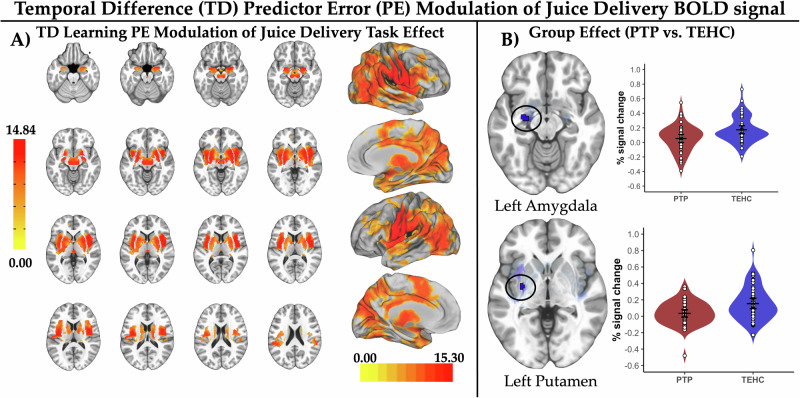
Temporal difference prediction error modulation of juice delivery activation and post-trauma psychopathology abnormalities. **A** Depicts the reward region of interest (ROI)-constrained patterns of activation modulation by temporal difference (TD) learning model prediction errors (PEs) for blood oxygenation level dependent (BOLD) responses to juice delivery (brain slice montage displayed on the MNI152 ICBM 2009c non-linear asymmetric average brain) as well as patterns of TD learning PE modulation of BOLD response to juice delivery in the whole brain exploratory analysis (projected onto an average brain surface). **B** Depicts abnormalities in TD learning PE modulation of juice delivery BOLD signal for the post-trauma psychopathology (PTP) vs. trauma-exposed healthy comparison (TEHC) group identified in the ROI analysis at a pTFCE-corrected p_CW_ < 0.05. Results are displayed on the MNI152 ICBM 2009c non-linear asymmetric average brain. Suprathreshold voxels are outlined in black with a linear fade applied to subthreshold voxels as a function of distance from significance threshold. Violin plots depict average fMRI % signal changes for TD learning PE modulation of activation to juice delivery for each participant averaged over suprathreshold voxels. White dots indicate observed mean values for each participant averaged over suprathreshold voxels. Widest black horizontal line within each group violin plot indicates the mean value, with the black lines above/below indicating the 95% confidence interval for the group mean.

We observed positive modulation of BOLD signal by TD learning PEs bilaterally in amygdala, midbrain, putamen, pallidum, insula, caudate body/tail, and unilaterally in the left pgACC (Fig. 5A and Supplementary Table 10). No areas showed negative TD learning PE modulation. Consistent with hypotheses and results from the activation analysis, the PTP vs TEHC group showed attenuated juice delivery TD learning PE modulation of BOLD signal in the left amygdala and left putamen (pTFCE-corrected p_CW_ < 0.05; Fig. 5B and Supplementary Table 11). The effect in the left putamen survived the more stringent pTFCE-corrected p_EW_ < 0.05 threshold, but the effect in the left amygdala did not.

### Abnormal TD learning PE fMRI encoding-symptom relationships

There were no relationships between abnormal TD learning PE BOLD signal encoding to juice cue or delivery and symptom measures surviving Type I error correction.

## Discussion

Here, we probed an understudied component of reward processing in human psychiatric neuroscience—temporal violations in cue-reward timing. Results replicate and extend prior findings [56] by identifying the left putamen and other reward circuit areas as producing a signed temporal PE, i.e., increasing activity for positive temporal PEs and decreasing activity for negative temporal PEs. Model-based fMRI assessed neural encoding of TD learning PEs and produced patterns of BOLD signal modulation broadly consistent with dopamine receptor distributions [82], the neurotransmitter with the strongest evidence for TD learning-like computations [83], and findings of prior studies [48, 60, 84, 85]. This provides additional evidence that TD learning models distinct computations in neural circuits. We report the following primary findings. First, PTP is associated with attenuated positive temporal PE activation in the left amygdala, putamen/pallidum, and midbrain. Second, PTP is associated with attenuated negative temporal PE deactivation in the left amygdala and putamen/pallidum. These findings were recapitulated in the model-based analysis as attenuated encoding of TD learning PEs in the left amygdala and putamen at times of juice receipt. Third, more profound failure to deactivate the left amygdala to negative temporal PEs was associated with more intact hedonic capacity in those with PTP. Fourth, we observed abnormally heightened PTP encoding of TD learning PEs to juice-predictive cues in the bilateral insula and putamen. In sum, these findings provide initial evidence of amygdala, striatal, and insular dysfunction in PTP during violation of cue-reward timing relationships. Surprisingly, they also illustrate the uncommon scenario wherein abnormal activity of a brain region (the amygdala) that may be indicative of pathology in some contexts (e.g., fear/emotional reactivity) [86] can also be associated with facets of health in other contexts (unrealized reward delivery at an expected time).

Hypotheses for PTP-related abnormalities were partially supported. We observed no differences in activation to reward vs. non-reward cues or expected outcomes but instead observed PTP-related abnormalities in activation to temporal PEs and in TD learning PE encoding at times of juice cue and outcome delivery. Though use of a primary reinforcer was a novel aspect of this study [7, 38], juice-predictive cues and delivery failed to elicit abnormal brain activity in PTP. This may indicate that overall neural response to temporally expected primary rewards (such as a juice bolus) and their predictive cues is not a major facet of PTP-related dysfunction. However, other aspects of cue-reward timing, i.e., neural responses to violations of learned temporal relationships and neural encoding of TD learning PE computations, did produce notable differences in the PTP group. Specifically, PTP was associated with blunted signaling of both temporal PEs and TD learning PEs in the left amygdala and left putamen/pallidum, which raises the possibility of underlying abnormal TD learning computations in PTP. Blunted TD learning PE and temporal PE signaling is also consistent with findings from operant decision-making tasks using traditional RL models that demonstrate attenuated PE signaling in PTSD [32]. However, a key difference here is that type/quality of outcome following a predictive cue was totally consistent. Only the timing of outcomes varied. Thus, attenuated signaling of reward cue/outcome timing deviations may be a previously unrecognized but clinically relevant facet of PTP neural pathophysiology.

Intriguingly, greater failure to deactivate the amygdala to negative temporal PEs was associated with greater PTP hedonic capacity. Regression mapping likewise identified less deactivation of a left midbrain region as associated with greater hedonic capacity, though overall level of activation in this area was not abnormal. Although amygdala hyperactivity to negative emotional stimuli is frequently observed in PTSD [87–89] and other psychiatric disorders [90], we speculate such hyperactivity may confer a paradoxical benefit in this context. Preclinical work indicates a critical role for the amygdala in signaling negative temporal PEs in a reinforcement context [91]. The amygdala is also known to mediate attention towards stimuli signaling reward or punishment [92, 93] and vigilance towards both outcomes [94]. VTA-to-amygdala dopamine projections are important mediators of salient somatosensory events [95] as well as rewards [96]. Taken together, we speculate these relationships may illustrate in PTP a distinct neurobiological correlate of sustained vigilance to an upcoming expected reward when its delivery is beyond the temporal window of expected receipt. Specifically, failure to deactivate this structure could reflect an adaptive manifestation of amygdala-mediated heightened vigilance processes (i.e. vigilance for expected reward, not only expected threat). Such sustained reward vigilance could promote greater emotional resilience to disappointing outcomes or unexpected reward omissions in daily life, which may bolster or sustain hedonic capacity. Future targeted studies are needed to test this hypothesis.

We unexpectedly found exaggerated TD learning PE encoding to juice-predictive cues in the insula and putamen bilaterally in PTP, which diverges from attenuated TD learning PE encoding observed during juice receipt. From the TD learning perspective, this is consistent with an accelerated rate of back-propagation of expected value from reward to predictive cue, which would manifest as exaggerated TD learning PE encoding at cue presentation but diminished encoding at reward receipt. Insula hyperactivation is frequently observed in PTSD during emotional reactivity or fear processing [87, 89]. Given the role of the insula in temporal prediction [97, 98] and physiological state forecasting [99] and the role of the putamen in time perception [100] and tracking both reward prediction and reward history [101], we speculate exaggerated insular/putamen TD learning PE signaling in PTP may reflect elevated neural responsivity to the cue-signaled upcoming deviation from the current interoceptive/reward state. Given absence of significant relationships between TD learning PE encoding abnormalities and clinical measures, the (mal)adaptiveness of these changes remain unclear. Regardless, findings highlight the relative value of employing both traditional and model-based fMRI approaches to reveal complementary information in suitable tasks.

There are several limitations to consider. First, as a cross-sectional study, we are limited in attribution of abnormalities to disorder development vs. premorbid characteristics. This is an important consideration given that the two groups, in addition to current trauma symptoms, also differed on symptom chronicity and trauma characteristics that tend to predict development of PTSD, i.e., personal experience of physical and sexual assault [102, 103] and severity of childhood maltreatment [104]. Thus, group differences could also reflect effects pertaining to these factors. Second, results may not be fully representative of the full spectrum of PTP, specifically trauma-related MDD without PTSD. Although a transdiagnostic study, our inclusion criteria proved to be highly specific for current PTSD. This may relate to the MASQ-AD inclusion criterion being derived from a diagnostic PTSD sample and/or sampling for chronic symptoms with a clear temporal onset following a Criterion A traumatic event. Third, due to equipment constraints, the comparator outcome (visual cue) was in a different sensory modality then that of the reward stimulus (juice bolus). A less-rewarding gustatory stimulus, such as artificial saliva, would have facilitated stronger inference on rewarding properties of the juice bolus, which cannot be separated from perceptual modality effects here. However, given that prior studies employing this paradigm have not used any comparator condition [56–58], the current design could still be considered an incremental advance. Fourth, positive temporal PEs only followed induction of negative temporal PEs (unexpected outcome absence) in catch trials. It is possible that induction of positive temporal PEs without preceding negative temporal PEs, i.e., delivery of outcome prior to expected temporal window, could induce different effects. We maintained this fixed order since it allows for elicitation of both types of temporal PEs in the same trial (not possible with positive temporal PE induction prior to expected outcome delivery period). This mitigates the need for substantially increasing task length to examine both types of catch trial sequences, i.e., a longer task would be necessary to preserve the overall catch to normal trial ratio, likely important for temporal PE induction. Fifth, since clinical and fMRI assessments were completed on separate visits, there is some temporal variability in the delay between the two visits across participants related to scanner scheduling/availability, which could impact the magnitude of brain-behavior associations. Sixth, our study was not adequately powered to maintain the fMRI ${\alpha }_{{EW}}$ at 0.05 when testing multiple fMRI contrasts, which would require a much larger sample size. We identify the effects which survive this more stringent correction, which increases confidence in their robustness. However, in addition to the possibility of the other findings reflecting false positives, it may also be the case that findings which did not survive this more stringent correction may be true differences but that our study was underpowered to reject the null hypothesis in these cases (Type II error). Therefore, findings should be considered preliminary until replicated in focused analyses or in larger, more well-powered samples.

In summary, we provide evidence for a previously unrecognized facet of positive valence dysfunction in PTP in a core reward processing domain—forming and updating expectations for timing of cued reward delivery. This dysfunction in neural responsivity and encoding of reward timing deviations was elicited by a simple Pavlovian reward learning task, demonstrating the pervasive nature of PTP reward-related neural circuit alterations and grounding understanding of these changes in the context of a core learning process shared across species. The potential clinical relevance of this dysfunction is illustrated by the intriguing relationship between amygdala negative temporal PE deactivation failure and preserved hedonic capacity. This counterintuitive finding underscores the necessity of assaying neural circuit function across various domains and behaviors to fully characterize adaptive and maladaptive abnormalities in the broadest context. Whereas amygdala dysfunction is classically viewed as a maladaptive facet of PTSD in the context of fear extinction and threat reactivity [86], the current findings bring additional attention to the multifaceted roles of brain regions/circuits across neurobehavioral processes—a functional characteristic maladaptive in one context/environment may be adaptive in another. For example, it has been proposed that amygdala hyperactivity in children reared in early adverse environments may be initially adaptive (to facilitate enhanced threat detection) but may then also predispose individuals to future development of psychopathology [105]. These and other findings exemplify the complex interplay between one’s biology and environment. Ongoing characterization of neural and computational processes underlying reward and positive affect in those suffering from post-trauma mental health difficulties is therefore of high importance and may lead to new opportunities for enhanced therapeutics.

## Supplementary information

Supplemental Materials

## Data Availability

Deidentified data are available upon reasonable request from the corresponding author.
