# Supplementary material for: Altered amygdala and striatal responsivity and prediction error encoding of temporal reward dynamics in post-trauma psychopathology
Source: Neuropsychopharmacology. 2025 Nov 17;51(5):904–15. doi: 10.1038/s41386-025-02276-z (PMC13013824; doi:10.1038/s41386-025-02276-z)
Supplement: Supplementary file 1 — Supplemental Materials [file 41386_2025_2276_MOESM1_ESM.docx]

**Supplemental Methods**

*Participants/Screening*

Exclusion criteria were history of neurological disorders, epilepsy, stroke, brain surgery, electroconvulsive or radiation treatment, brain hemorrhage/tumor, or thyroid disorder; current use of benzodiazepines or opiates; use of daily psychiatric medications (antidepressants, anticonvulsants, antipsychotics, stimulants), current evidence-based psychotherapy incorporating prominent cognitive-behavioral components, history of psychosis or bipolar disorder, regular nicotine/tobacco use (more than once per week, to rule out potential confounding effects of nicotine on brain function), active substance dependence (past 6 months), self-reported suicide attempt in the past year, and contraindications for MRI. Individuals first completed a brief screening on REDCap including the 30-item Mood and Anxiety Symptom Questionnaire (MASQ) [1], demographic questions, Life Events Checklist [2], and a subsequent self-report probe of PTSD symptoms based upon the Mini International Neuropsychiatric Interview (MINI) [3].

*Passive Reward Learning Task*

Participants were informed they would see a white fixation cross for most of the scan, which would periodically be replaced by one of two 1-second visual cues (a blue circle or a red diamond, both on a black background). When they saw the blue circle, they were instructed to press the first button on an MRI-compatible button box; when they saw the red diamond, they were instructed to press the second button. Button presses were used to keep participants engaged throughout the task. Participants were informed that one of those visual cues would reliably predict oral delivery of a small amount of juice, about 2-4 sec later, through a plastic tube that would be held in their mouth during the scan. The other visual cue would reliably predict delivery of another distinct neutral visual cue, also about 2-4 sec later. Participants were instructed to stay awake and press the appropriate buttons when they saw visual cues. They were told there would be two runs of this task, each requiring the same behavior and with the same stimuli.

*MRI Acquisition and Juice Delivery*

After changing into hospital scrubs and final safety checks, participants were deposited inside the scanner. Head motion was minimized through memory foam padding placed on the sides of the head with an additional pad placed between the forehead and top of the head coil. Scan session began with collection of a T1-weighted high resolution anatomical image (3D MPRAGE, sagittal, TR/TE = 2400/2.18 ms, slice thickness = 0.8 mm, flip angle = 8 deg, FOV = 256 mm, matrix size = 200 x 308 x 320, voxel size = 0.8 x 0.8 x 0.8 mm, duration = 6:38). The exam table was then slid out from the scanner bore, and the terminal end of about ~33 feet of food-grade plastic tubing filled with the participant’s juice of choice was then placed 1-2 cm into the supine participant’s left corner of mouth horizontally (i.e. parallel to the cheek bone, such that there was a downward arc to the tubing emerging from the head coil) and affixed with waterproof tape to the head coil. Horizontal placement in the participant’s mouth with a downward vertical arc was used to prevent air bubbles from forming, established through piloting. When the tubing was placed comfortably, the exam table was then slid back into the scanner bore, and the tubing was also taped to the side of the scanner bore. The other end of the juice-filled tubing was connected, through the scanner room penetration panel, to a juice-filled Monoject 140 ml oral syringe seated inside a Harvard Apparatus Remote Infuse/Withdraw PHD ULTRA™ 4400 Programmable Syringe Pump (Harvard Apparatus, Holliston, MA) on a wheel cart inside the scanner control room. The syringe pump was triggered through commands delivered via USB cable from a Dell Latitude laptop that was also used for task presentation. Syringe pump was set to infuse at 2ml/sec and triggered to run for 0.5 sec (delivering ~1 ml of juice per bolus). The passive reward learning task was delivered during acquisition of T2*-weighted images sensitive to blood oxygenation level-dependent (BOLD) response (TR/TE = 665 ms/30 ms, slice thickness = 2.5 mm, anterior-to-posterior phase encoding direction, 54 slices, flip angle = 52 deg, FOV = 215 mm, matrix size = 86 x 86 x 54, voxel size = 2.5 x 2.5 x 2.5 mm, duration = 8:41 for Run1 and 13:26 for Run 2, SMS factor = 6). T2*-weighted images with identical parameters but opposite phase encoding direction were acquired prior to the task run for susceptibility distortion correction.

*Passive Reward Learning Task*

The first task run encompassed 54 trials of cue-outcome contingency learning, 27 of each condition. Trials were pseudo-randomly ordered, which was held constant across participants. Each trial began with a 1-sec visual presentation of a blue circle or red diamond on a black background, which was then replaced by a white fixation cross. Two to four seconds (jittered by 1 sec, with a mean of 3 sec across trials) following offset of the cue, participants reliably received either a 1ml juice bolus (following blue circle, with white fixation cross remaining on screen) delivered over the course of ~1 sec or were presented with a third visual cue, a black/white patterned triangle presented on black background, also for 1 sec (following red diamond). Participants then viewed a white fixation cross for 3-5 sec (jittered by 1 sec with a mean of 4 sec across trials) prior to the start of the next trial. Each trial lasted ~9 sec in total, and temporal relationships between cue and outcome were entirely consistent (2-4 sec delay). A white fixation cross was also presented at the start and end of each run for 10 sec. The second task run encompassed 72 trials: 26 temporally consistent trials of each type (juice and visual cue outcome) and 10 “catch” trials of each type, where the delay between cue and outcome was extended to 8-10 sec (jittered by 1 sec, mean of 9.3 sec across trials of each type). “Catch” trials were placed in the latter 75% of trials on this run to maximize learning of the cue-outcome timing associations. Furthermore, a catch trial of each type (juice or visual cue) was always followed by one or more “normal” trials of the same type prior to the next catch trial presentation to re-instate the temporal expectation and maximize prediction errors. Catch trials lasted for ~15 sec in total, maintaining the typical 3-5 sec interval between outcome delivery and start of the next trial.

*Visual Analogue Scales*

Each scale was 10 inches long. For thirst, it ranged from “Not at all thirsty” to “Very thirsty”; for enjoyment, it ranged from “Very unenjoyable” to “Very enjoyable”; and for taste, it ranged from “Not well at all” to “Very well.” Measurements were made to the closest 1/16^th^ of an inch and were anchored to the center point, which was considered 0. Thus, each measurement ranged from -5 to 5, with positive values indicating greater thirst, enjoyment, and ability to taste.

*fMRI Preprocessing*

Preprocessing occurred in FSL [4]. Affine transformation of functional to structural images was added to non-linear normalization of each participant’s T1 image to the Montreal Neurological Institute (MNI) 152-person 1 mm^3^ T1 template using FNIRT from FSL 5.0 [5]. Functional images were re-aligned to the middle volume of the run using rigid-body motion correction (FSL’s mcflirt), and FSL’s topup program was used to construct a susceptibility-induced off-resonance field [6] which was then used to apply a correction to the data from the task functional run. Motion and susceptibility distortion-corrected functional data from each participant was then normalized to MNI atlas space and resampled to a 2 x 2 x 2 mm voxel size. Functional data was smoothed with an isotropic 6 mm full-width half max Gaussian kernel to account for individual anatomical variability. For quality control, participants were set to be excluded a priori if they had a root mean square absolute movement > 4 mm across the mean of the squared maximum displacements in each of the 6 translational and rotational motion parameters. However, all participants met this criterion (mean/max root mean square of absolute displacement = 0.72/3.78 mm).

*Traditional Individual-Level fMRI Activation Analysis*

Using the afni_proc.py function in AFNI [7], individual time series images for each run were scaled to percent signal changes. Boxcar regressors modeling onset and duration of events for juice and non-juice trials were included (as separate regressors) These included predictive cues, temporally predicted outcomes (modeled as 1 sec durations, with juice bolus onset shifted an additional 0.5 sec from trigger related to compliance in plastic tubing; delay and duration of flow determined through empirical testing), absence of outcome at expected trial times (onset 2 sec following outcome delivery with 1 sec duration, where there could be no expectation for stimulus delivery), absence of outcomes at unexpected times (4 sec following offset of cue on catch trials, i.e. very end of typical temporal window for outcome delivery, with 1 sec durations and an additional 0.5 sec onset delay for juice trials), and receipt of outcomes at unexpected trial times (time of outcome delivery on catch trials, which was 8-10 sec following cue with an additional 0.5 sec delay for juice bolus delivery and 1 sec durations). All were convolved with the HRF. Additional regressors of no interest for both runs included 6 motion regressors (translations and rotations in x, y, and z dimensions) and second-order Legendre polynomials to account for scanner intensity drift over time. Volumes with motion exceeding 0.3 of the Euclidean norm of motion parameter derivatives (and preceding volumes) were censored from analysis. The program 3dDeconvolve was used to set up the deconvolution matrix, which was then executed by 3dREMLfit to estimate effects using generalized least squares time series fit with restricted maximum likelihood (REML) estimation of temporal autocorrelation.

From these events, we calculated several within-subject contrasts of interest. First, we contrasted juice vs. non-juice cues. Second, we contrasted juice vs. visual cue outcome for normal trials. Third, for both juice and non-juice trials, we calculated separate contrasts capturing positive temporal PEs, i.e. unexpected outcome receipt on catch trials vs. expected outcome receipt on normal trials. This isolates better-than-expected momentary reward value controlling for stimulus delivery (outcome receipt on catch trials at the unexpected trial time vs. outcome receipt on normal trials at the expected trial time). Fourth, for both juice and non-juice trials, we calculated separate contrasts capturing negative temporal PEs, i.e. unexpected outcome absence on catch trials vs. expected outcome absence on normal trials. This captures worse-than-expected momentary reward value controlling for stimulus absence (absence of outcomes on catch trials at unexpected trial times, i.e. the time window when the outcome is normally present vs. outcome absence on normal trials at times when the outcome is normally absent, i.e. at the very end of the trial following outcome receipt). Fifth, we calculated the contrast of juice vs. non-juice positive temporal PEs. Sixth, we calculated the contrast of juice vs. non-juice negative temporal PEs. As in prior work [8], unexpected vs. expected receipt or absence of juice delivery contrasts were used to estimate positive and negative temporal PE signaling in the brain, respectively. Here, we further contrasted these between juice and non-juice trials to better isolate brain activity ostensibly specific to juice/reward-related temporal PEs.

*Temporal Difference Learning Model*

The temporal difference (TD) learning model used in this study was initially proposed by Schultz et al. [9] and utilized by O’Doherty et al. [10] to examine fMRI BOLD correlates of TD learning PEs. The model and underlying theory is explained in detail in those publications. On each trial, the predicted value at any time *t* (*V_t_* ) is used to generate a PE at time *t* (δ). PEs are calculated as the difference between: a) the sum of any reward delivered at that time (*R_t_* ; coded as 1 for delivered and 0 for not delivered) plus the predicted value at the next time step (*V_t + 1_*) weighted by a temporal discounting factor (λ, ranging from 0 to 1 with higher values placing greater emphasis on proximal rewards); and b) the predicted value at time *t* (*V_t_* ). This is consistent with standard reinforcement learning (RL) models (e.g., Rescorla-Wagner), except for the addition of the temporally discounted predicted value at the next time step. A key difference, however, between standard RL and TD learning is that the expected value signaled by each predictive cue is represented mathematically as a vector, with each vector value corresponding to a particular time point following each cue up until receipt of the reward/outcome. Let *X*(t) = [X_1_(t), X_2_(t), …X_n_(t)], such that X_i_(t) corresponds to exactly *i* time steps into the future following a cue, up until n (total number of time steps in a trial until stimulus receipt). X_i_(t) will be 1 for each instance of that time step following a cue presentation and 0 otherwise. Each time step following a cue, X_i_(t), has a weight associated with it, W_i_(t). When one or more predictive cues are perceived, the predicted value at each trial time step *t* is represented as the summed product of weights across predictive cues at that time step and the stimulus representation vector, such that *V_t_ =* $\sum W_{i}X_{i}(t)$. In this study, since there was only a single outcome-predictive sensory cue (a visual stimulus) signaling each outcome type, the predicted value estimate at each time t only incorporated the weights of a single visual stimulus. On the first trial, *V_t_ =* 0 since all weights are initially set at 0. When the first reward/outcome is delivered, PEs are generated as described above, and then each time step weight for predictive cues present on that trial are updated proportional to a learning rate (α, ranging from 0 to 1) and the PE at each instance of that time step: $\Delta W_{i}= \alpha\sum X_{i}(t)$δ(t). Updated weights are then available on the next predictive cue presentation for updated estimates of *V_t_*. See Supplemental Figure 1 for a visual depiction of the TD learning process over time.

The time step for the model was set to the temporal resolution of the fMRI data (1 TR = 0.665 ms) and initial predicted values for each cue were 0 as were initial timestep weights. Delivery of an outcome at time *t* (juice bolus or visual cue) was coded as 1 and 0 otherwise, Since the values of α and λ are arbitrary weights that need to be set *a priori*, we employed the values of α = 0.2 and λ = 0.9 since: a) these values provided a conceptually plausible estimate of model-estimated PEs such that learning and backward propagation of PEs from outcomes to predictive cues was gradual; b) similar values were found in prior work [10,11] to provide the best representation of fMRI TD learning PE encoding in reward circuit regions (ventral striatum, midbrain) with a strong empirical evidence base for TD learning computations; and c) in the current study, these values provided good convergence of model-free (positive vs. negative temporal PEs) and model-based fMRI maps of TD learning PE signal encoding. Each predictive cue (one predicting juice, the other predicting another visual cue) was each assigned its own vector of weights. The vector of weights for that cue type was successively updated after each trial of that cue type based upon the PEs generated at each time step in that trial. This model resulted in a model-predicted PE value at each time step of the task, which was then used to examine fMRI BOLD signal encoding of TD learning PE signaling.

*Core/Extended Reward-Responsive Regions of Interest*

We conducted group-level analyses within a region of interest (ROI) mask encompassing core and extended reward-responsive regions. These encompassed: a) bilateral amygdala (from FSL’s subcortical segmentation program)[12]; b) striatum and midbrain (derived from the California Institute of Technology 168-subject Reinforcement Learning (CIT168-RL) atlas including (all bilaterally) caudate, putamen, nucleus accumbens, globus pallidus internal and external, ventral pallidum, substantia nigra pars compacta and pars reticulata, subthalamic nuclei, hypothalamus, parabrachial pigmented nuclei, ventral tegmental area, red nuclei, habenular nuclei, and mammillary nuclei)[13]; c) insula (defined by Automatic Anatomical Labeling (AAL) atlas)[14]; and d) medial prefrontal regions (anterior cingulate cortex, mid-cingulate cortex, and olfactory cortex sites of AAL atlas with y > 0, -14 < x < 14, and -12 < z < 44).

**Supplemental Results**

*Visual Analog Scales*

Greater thirst was associated with greater enjoyment (ρ_87_ = 0.37, *p* < 0.001), and greater enjoyment was associated with better ability to taste (ρ_87_ = 0.27, *p* = 0.01). In examining group differences on each dimension, we controlled for relationships with the other two. The PTP group displayed less enjoyment of the juice vs. the TEHC group (*F_1,85_* = 4.07, *p* = 0.047) but did not differ on capacity for taste or degree of thirst (*p*’s > 0.36).

*fMRI Activation (Whole-Brain Findings)*

Juice vs. Non-Juice Cue: The whole brain (WB) analysis showed task effects of lower activation to the juice vs. non-juice cue in the cerebellum, occipital cortex, posterior temporal cortex, and parietal cortex (Supplemental Figure 2 and Supplemental Table 2). There were no significant PTSD vs. TEHC group activation differences in the WB analysis.

Juice vs. Visual Cue Delivery at Expected Times: The WB analysis showed task effects of greater activation to juice vs. visual cue delivery at expected times in bilateral motor/sensory cortex and inferior parietal cortex and deactivation (greater activation to visual cue vs. juice delivery at expected times) in bilateral cerebellum, visual cortex, superior parietal cortices, anterior medial PFC (mPFC), and dorsolateral PFC (dlPFC) (Supplemental Figure 3 and Supplemental Table 3). There were no significant PTSD vs. TEHC group activation differences in the WB analysis.

Juice vs. Visual Cue Positive Temporal PEs: In the WB analysis, greater task-related activation for juice vs. visual cue positive temporal PEs was observed bilaterally in the cerebellum, occipital cortex, posterior temporal cortex, thalamus, and motor/sensory cortices, with unilateral effects in right OFC, left dorsal ACC (dACC), and right dlPFC (Figure 2 and Supplemental Table 4). No areas demonstrated greater activation to visual cue vs. juice positive temporal PEs. There were no significant PTSD vs. TEHC group activation differences in the WB analysis.

Juice vs. Visual Cue Negative Temporal PEs: The WB analysis revealed greater task-related juice vs. visual cue negative temporal PE deactivation (or greater visual cue vs. juice negative temporal PE activation) in bilateral cerebellum, visual cortex, sensory/motor cortices, OFC, vmPFC, thalamus, dACC, mid-cingulate, dorsomedial PFC (dmPFC), dlPFC, and inferior/superior parietal cortices (Figure 3 and Supplemental Table 6). There were no significant PTSD vs. TEHC group activation differences in the WB analysis.

*Model-Based fMRI encoding of Juice TD Learning PEs (Whole Brain Findings)*

TD Learning PE Modulation of Juice Cue BOLD signal: The WB analysis revealed additional task-related positive BOLD signal modulation by TD learning PEs in the cerebellum, bilateral hippocampus, right OFC, bilateral temporooccipital cortex, left lateral frontopolar cortex, thalamus, bilateral dlPFC, bilateral motor/sensory cortices, and superior parietal cortex. Additional task-related negative modulation by TD learning PEs was observed in the vmPFC, dmPFC, and supplementary motor area (SMA) (Figure 4 and Supplemental Table 8). The WB analysis revealed additional exaggerated modulation for the PTSD vs. TEHC group in the right vlPFC and supramarginal gyrus and replicated ROI effects in the bilateral insula and putamen (Figure 4 and Supplemental Table 9). These effects also survived the more stringent correction of pTFCE-corrected p_EW_ < 0.05.

TD Learning PE Modulation of Juice Delivery BOLD signal: In the WB analysis, task-related positive modulation of BOLD signal was additionally noted bilaterally in the cerebellum, brainstem, occipital cortex, inferior/superior temporal cortex, OFC, thalamus, dACC/mid-cingulate, dmPFC, dlPFC, posterior cingulate/precuneus, and inferior/superior parietal cortex (Figure 5 and Supplemental Table 10). No areas showed significant task-related negative modulation. There were no significant PTSD vs. TEHC group differences for TD learning PE modulation of BOLD signal to juice delivery in the whole-brain analysis.

*Model-Based fMRI encoding of Non-Juice (Visual Cue) TD Learning PEs*

TD Learning PE Modulation of Non-Juice Cue BOLD signal: The ROI-constrained analysis showed small areas of significant task-related positive modulation in the posterior insula and right caudate; negative task-related modulation was observed in the right vACC and left pgACC. The WB analysis revealed positive modulation in the right temporooccipital cortex and bilateral motor/sensory cortices and negative modulation in the right vACC (Supplemental Figure 5 and Supplemental Table 12). There were no significant PTSD vs. TEHC group differences for modulation of BOLD signal to the non-juice predictive cue in the ROI or whole-brain analyses.

TD Learning PE Modulation of Visual Cue Delivery BOLD signal: The ROI-constrained analysis revealed task-related positive modulation of bilateral lateral amygdala, midbrain, putamen, pallidum, dorsal anterior/ventral middle/posterior insula, and caudate body. No regions showed negative task-related modulation in the ROI analysis. In the WB analysis, positive modulation was observed bilaterally in the cerebellum, visual cortex, temporooccipital cortex, superior/inferior temporal cortex, thalamus, dACC/mid-cingulate, dmPFC, dlPFC, posterior cingulate/precuneus, and inferior/superior parietal cortex, with no areas demonstrating task-related negative modulation (Supplemental Figure 6 and Supplemental Table 13). The PTSD and TEHC groups did not differ on TD learning PE modulation of visual cue delivery BOLD signal in the ROI or whole-brain analyses.

**Supplemental References**

1 Wardenaar KJ, van Veen T, Giltay EJ, de Beurs E, Penninx BW, Zitman FG. Development and validation of a 30-item short adaptation of the Mood and Anxiety Symptoms Questionnaire (MASQ). Psychiatry Res. 2010;179(1):101-6.

2 Gray MJ, Litz BT, Hsu JL, Lombardo TW. Psychometric properties of the life events checklist. Assessment. 2004;11(4):330-41.

3 Sheehan DV, Lecrubier Y, Sheehan KH, Amorim P, Janavs J, Weiller E, et al. The Mini-International Neuropsychiatric Interview (M.I.N.I.): the development and validation of a structured diagnostic psychiatric interview for DSM-IV and ICD-10. The Journal of clinical psychiatry. 1998;59 Suppl 20:22-33;quiz 34-57.

4 Smith SM, Jenkinson M, Woolrich MW, Beckmann CF, Behrens TE, Johansen-Berg H, et al. Advances in functional and structural MR image analysis and implementation as FSL. Neuroimage. 2004;23 Suppl 1:S208-19.

5 Andersson JL, Jenkinson M, Smith S. (2010).

6 Andersson JL, Skare S, Ashburner J. How to correct susceptibility distortions in spin-echo echo-planar images: application to diffusion tensor imaging. Neuroimage. 2003;20(2):870-88.

7 Cox RW. AFNI: software for analysis and visualization of functional magnetic resonance neuroimages. Computers and biomedical research, an international journal. 1996;29(3):162-73.

8 McClure SM, Berns GS, Montague PR. Temporal prediction errors in a passive learning task activate human striatum. Neuron. 2003;38(2):339-46.

9 Schultz W, Dayan P, Montague PR. A Neural Substrate of Prediction and Reward. Science. 1997;275(5306):1593-99.

10 O'Doherty JP, Dayan P, Friston K, Critchley H, Dolan RJ. Temporal Difference Models and Reward-Related Learning in the Human Brain. Neuron. 2003;38(2):329-37.

11 O'Doherty J, Dayan P, Schultz J, Deichmann R, Friston K, Dolan RJ. Dissociable roles of ventral and dorsal striatum in instrumental conditioning. Science. 2004;304(5669):452-4.

12 Patenaude B, Smith SM, Kennedy DN, Jenkinson M. A Bayesian model of shape and appearance for subcortical brain segmentation. Neuroimage. 2011;56(3):907-22.

13 Pauli WM, Nili AN, Tyszka JM. A high-resolution probabilistic in vivo atlas of human subcortical brain nuclei. Scientific Data. 2018;5(1):180063.

14 Tzourio-Mazoyer N, Landeau B, Papathanassiou D, Crivello F, Etard O, Delcroix N, et al. Automated anatomical labeling of activations in SPM using a macroscopic anatomical parcellation of the MNI MRI single-subject brain. Neuroimage. 2002;15(1):273-89.

**Supplemental Figure 1. Temporal Difference Learning and Temporal Difference Learning Prediction Errors**

*This figure presents numerical data generated by the TD learning model in this study based upon the trials and temporal ordering of stimuli. The first column depicts the prediction errors (top row) and stimulus timestep weights on the first trial of learning, after a cue is presented and a stimulus is delivered. Since the cue is not known to predict anything, no prediction errors are generated except at the time of delivery of the stimulus. All timestep weights for the stimulus are furthermore set to 0 given no prior expectation associated with this stimulus. As the model learns the cue-outcome timing contingency, expected value (reflected in the timestep weights) and prediction errors begins to gradually propagate backwards towards the cue. This is reflected in the middle column, where prediction errors (top row) are generated at the time of cue presentation (since the cue is now known to carry information about upcoming future reward) as well as at the expected time of stimulus delivery (which now engenders a smaller prediction error signal since it is expected to some extent). Note how the stimulus weights (center column, bottom row) are now positive starting after cue onset and up until the time of stimulus delivery, which illustrates the backwards propagation of expected value from stimulus to cue. The rightmost column depicts stimulus weights and prediction error signaling on a later “catch” trial, after prior cue-outcome timing violations on prior catch trials have already been experienced. Once cue-outcome timing violations are introduced, the model still generates prediction errors at cue presentation (signaling upcoming future expected reward), but it also now generates negative prediction errors at the time of typical stimulus delivery when that stimulus is not delivered as expected (negative TD learning PEs). This is because the expected value at that time point (stimulus weight) is high given prior learning, and the violation of that expectation (current reward value worse than expected) generates a large negative TD learning PE. When the delayed/omitted outcome is delivered later in the same trial, the model generates a positive TD learning PE at those later timepoints, since the expectation for stimulus delivery at those later timepoints is much lower. This can be seen in the stimulus weights (lower right corner) at the time of the start of this catch trial, where expected value is still high at the time of typical reward delivery (though now a bit lower due to prior negative TD learning PEs induced through reward absence at this time in prior catch trials; compare stimulus weight at timepoint 9 in bottom right to stimulus weight at timepoint 9 in the center bottom panel) but there is now also a slight increase in expected value (stimulus weights) at later points in the trial since the stimulus was delivered at these unexpected later points in prior infrequent catch trials.*

**Supplemental Figure 2. Activation for Juice vs. Non-Juice Predictive Cue**

**
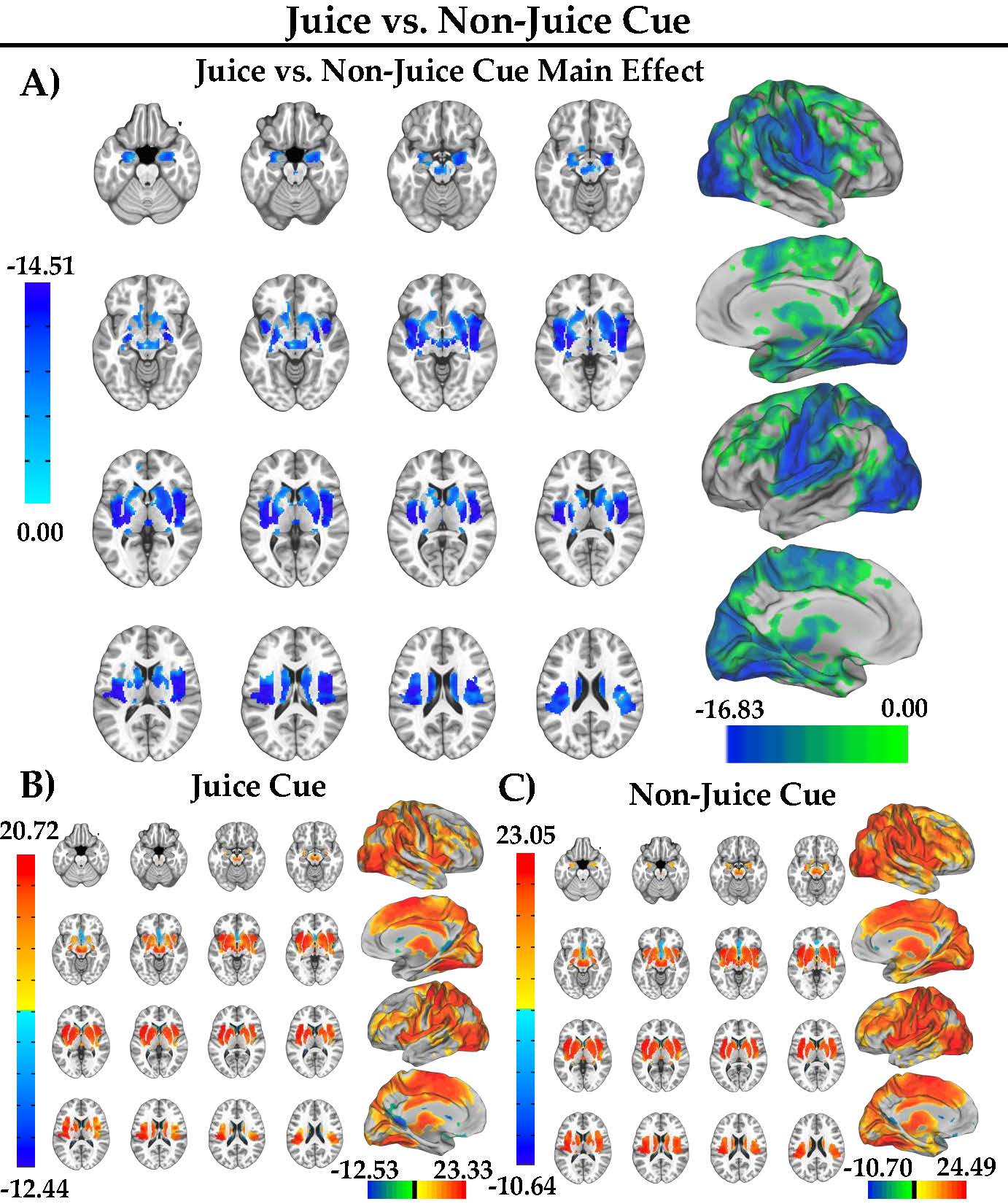
**

*Supplemental Figure 2A depicts the reward region of interest (ROI)-constrained patterns of deactivation for juice vs. non-juice predictive cues (brain slice montage displayed on the MNI152 ICBM 2009c non-linear asymmetric average brain) as well as patterns of deactivation in the whole brain exploratory analysis (projected onto an average brain surface for visual display). Supplemental Figure 2B and 2C depict the ROI-constrained patterns and whole brain activation patterns for each of the juice (2B) and non-juice predictive cue conditions (2C) separately, respectively. Color bars indicate the magnitude of the probabilistic threshold-free cluster enhancement (pTFCE)-corrected signed Z values (with positive indicating activation and negative indicating deactivation) displayed on the brain images.*

**Supplemental Figure 3. Activation for Juice vs. Visual Cue Delivery**


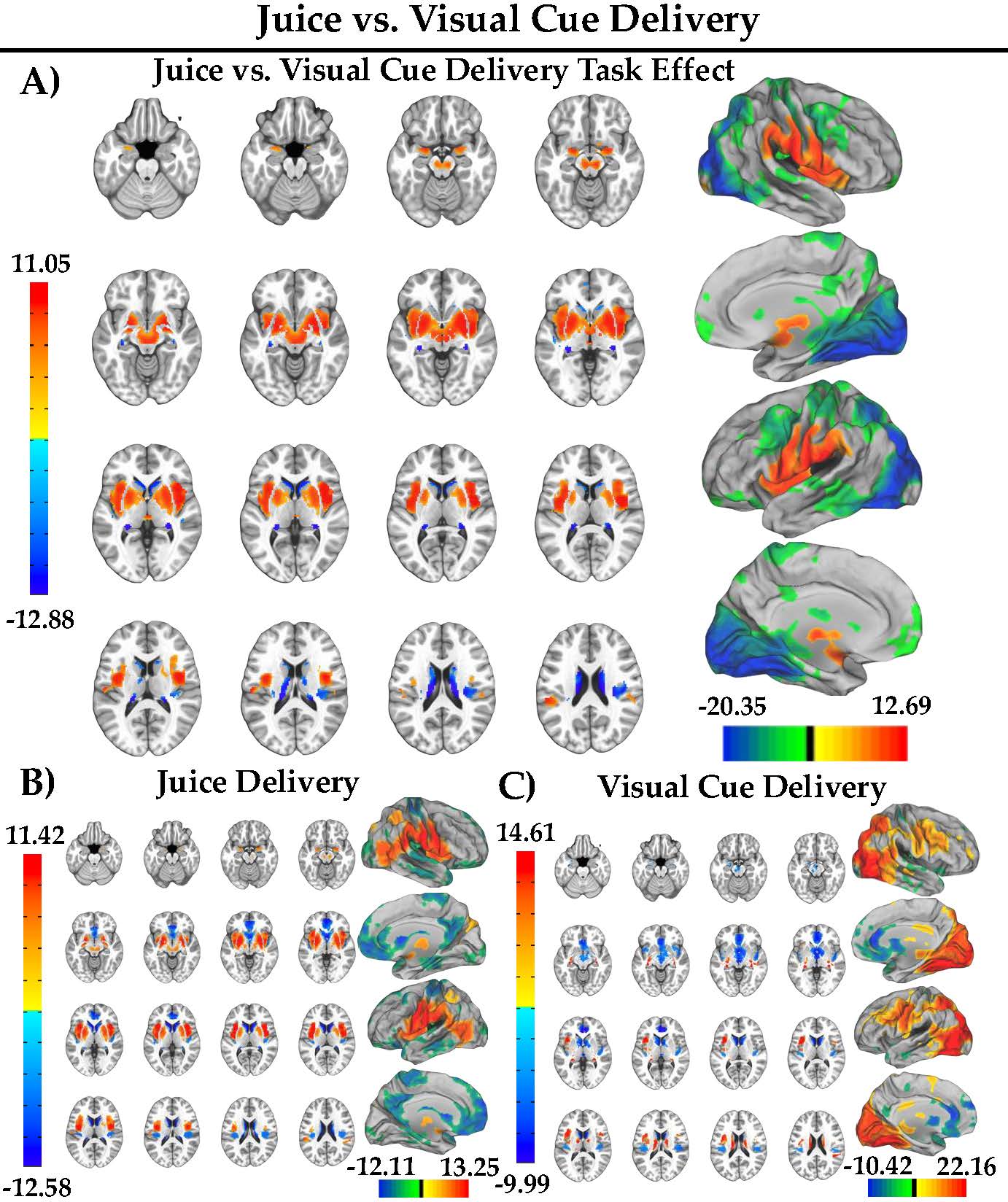


*Supplemental Figure 3A depicts the reward region of interest (ROI)-constrained patterns of activation for juice vs. visual cue delivery (brain slice montage displayed on the MNI152 ICBM 2009c non-linear asymmetric average brain) as well as patterns of activation in the whole brain exploratory analysis (projected onto an average brain surface for visual display). Supplemental Figure 3B and 3C depict the ROI-constrained patterns and whole brain activation patterns for each of the juice (3B) and visual cue delivery conditions (3C) separately, respectively. Color bars indicate the magnitude of the probabilistic threshold-free cluster enhancement (pTFCE)-corrected signed Z values (with positive indicating activation and negative indicating deactivation) displayed on the brain images.*

**Supplemental Figure 4. Post-Trauma Psychopathology Left Midbrain Negative Temporal Prediction Error Signaling Association with Anhedonia**

**
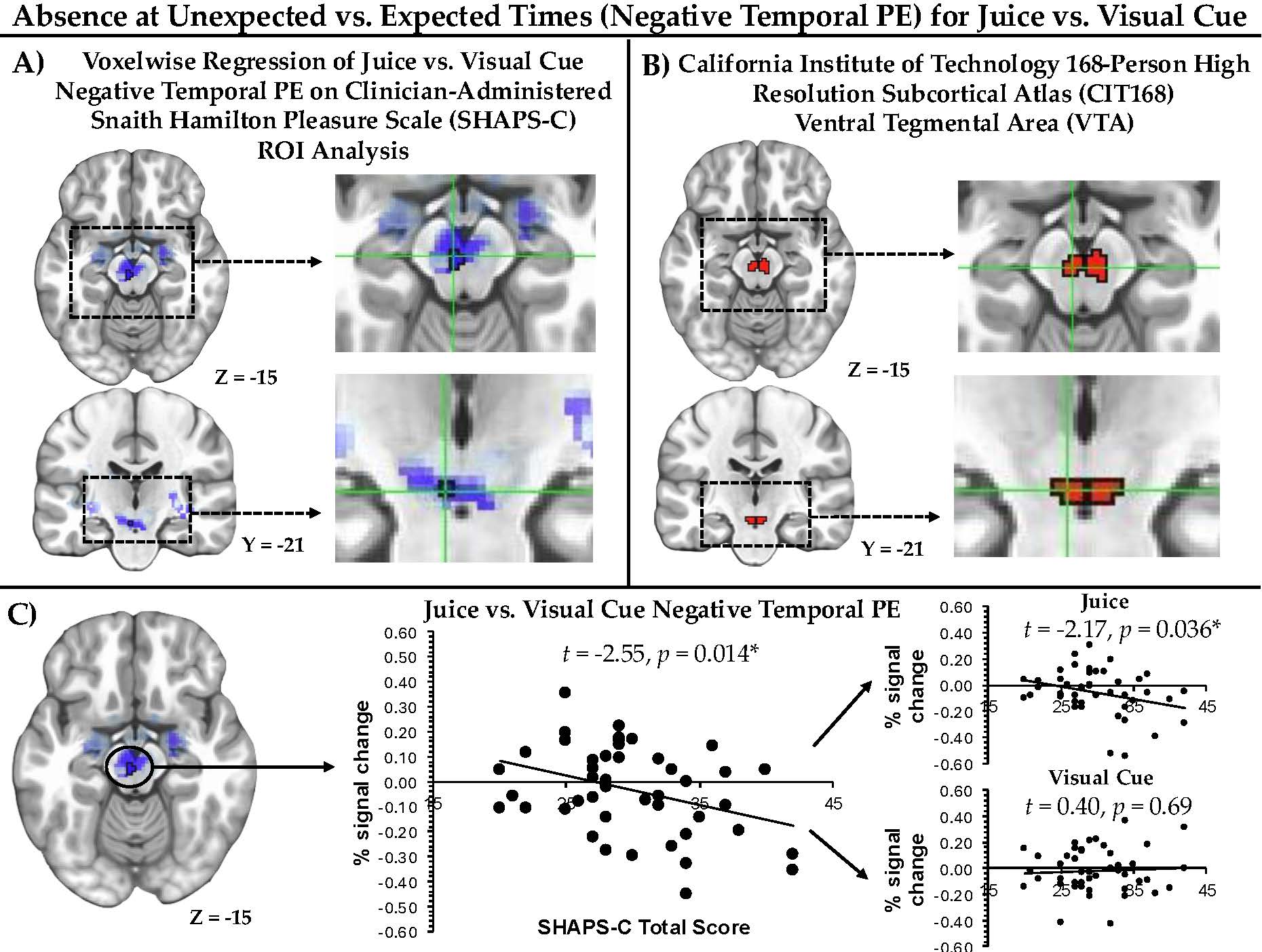
**

*Supplemental Figure 4A depicts the sole region identified by voxelwise regression of juice vs. visual cue negative temporal prediction error (PE) signaling on clinician-administered Snaith Hamilton Pleasure Scale (SHAPS-C) as displaying a statistically significant relationship (displayed on the MNI152 ICBM 2009c non-linear asymmetric average brain). Suprathreshold voxels are outlined in black with a linear fade applied to subthreshold voxels as a function of distance from significance threshold. Supplemental Figure 4B depicts the ventral tegmental area (VTA) regions of interest derived from the California Institute of Technology 168-person High Resolution Subcortical Atlas, with crosshairs in 3A and 3B centered on the same MNI coordinates (-4, -21, -15) to display the overlap of the detected effect with the left VTA region of interest (also displayed on the MNI152 ICBM 2009c non-linear asymmetric average brain). Supplemental Figure 4C depicts the scatterplot of the inverse relationship between juice vs. visual cue negative temporal PE deactivation and SHAPS-C total scores in this region, where greater activation/less deactivation was associated with less anhedonia. Far bottom right depicts the scatterplot of SHAPS-C total scores against juice and visual cue negative temporal PE deactivation separately to demonstrate the juice vs. visual negative temporal PE relationship with SHAPS-C total scores arises from the juice negative temporal PE contrast.*

**Supplemental Figure 5. Temporal Difference Prediction Error Modulation of Juice Non-Predictive Cue Activation.**

**
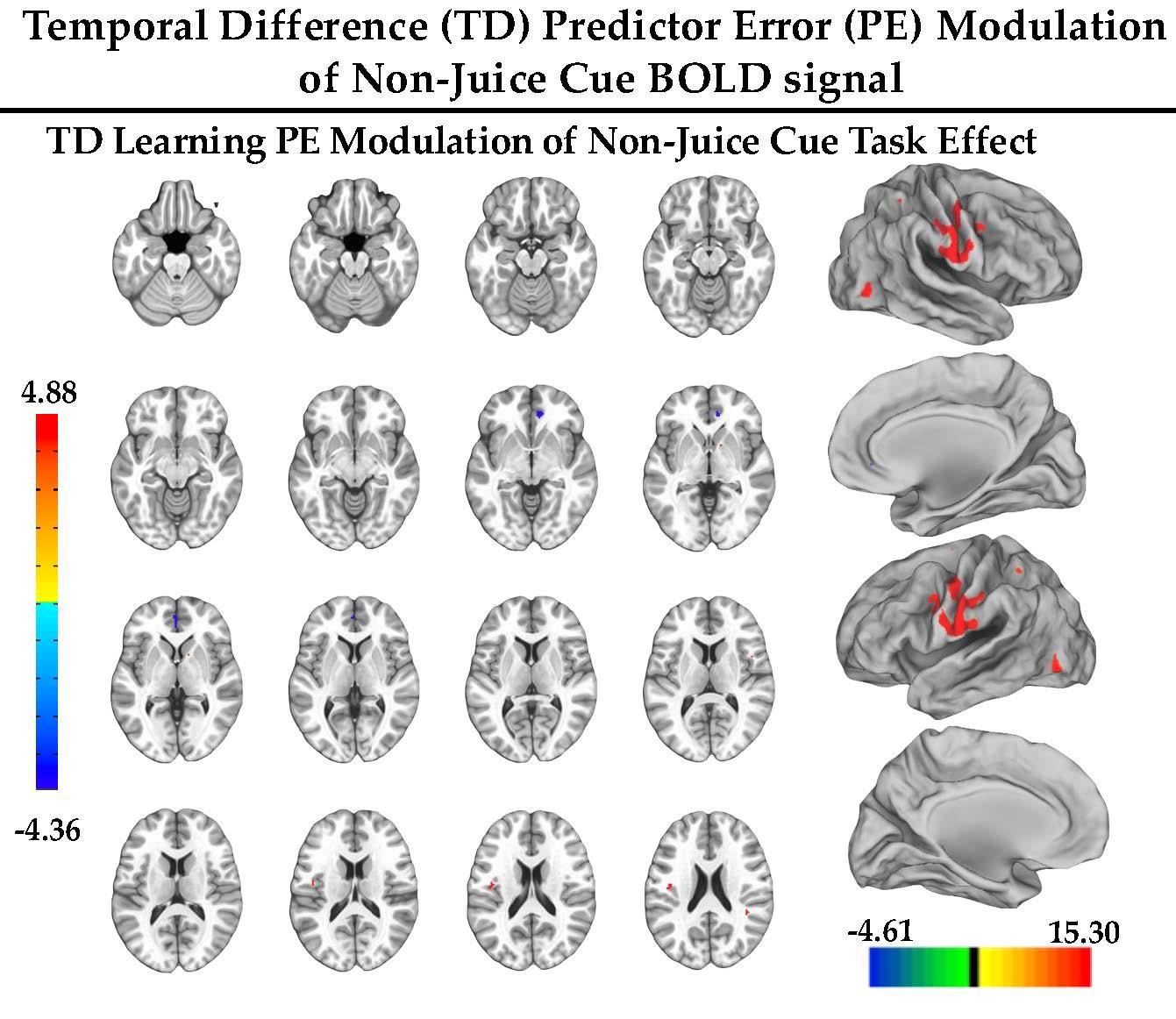
**

*Supplemental Figure 5 depicts the reward region of interest (ROI)-constrained patterns of activation modulation by temporal difference (TD) learning prediction errors (PEs) for blood oxygenation level dependent (BOLD) responses to the juice non-predictive visual cue (brain slice montage displayed on the MNI152 ICBM 2009c non-linear asymmetric average brain) as well as patterns of TD learning PE modulation of BOLD response to the juice non-predictive visual cue in the whole brain exploratory analysis (projected onto an average brain surface). Color bars indicate the magnitude of the probabilistic threshold-free cluster enhancement (pTFCE)-corrected signed Z values (with positive indicating activation and negative indicating deactivation) displayed on the brain images.*

**Supplemental Figure 6. Temporal Difference Prediction Error Modulation of Visual Cue Delivery Activation.**


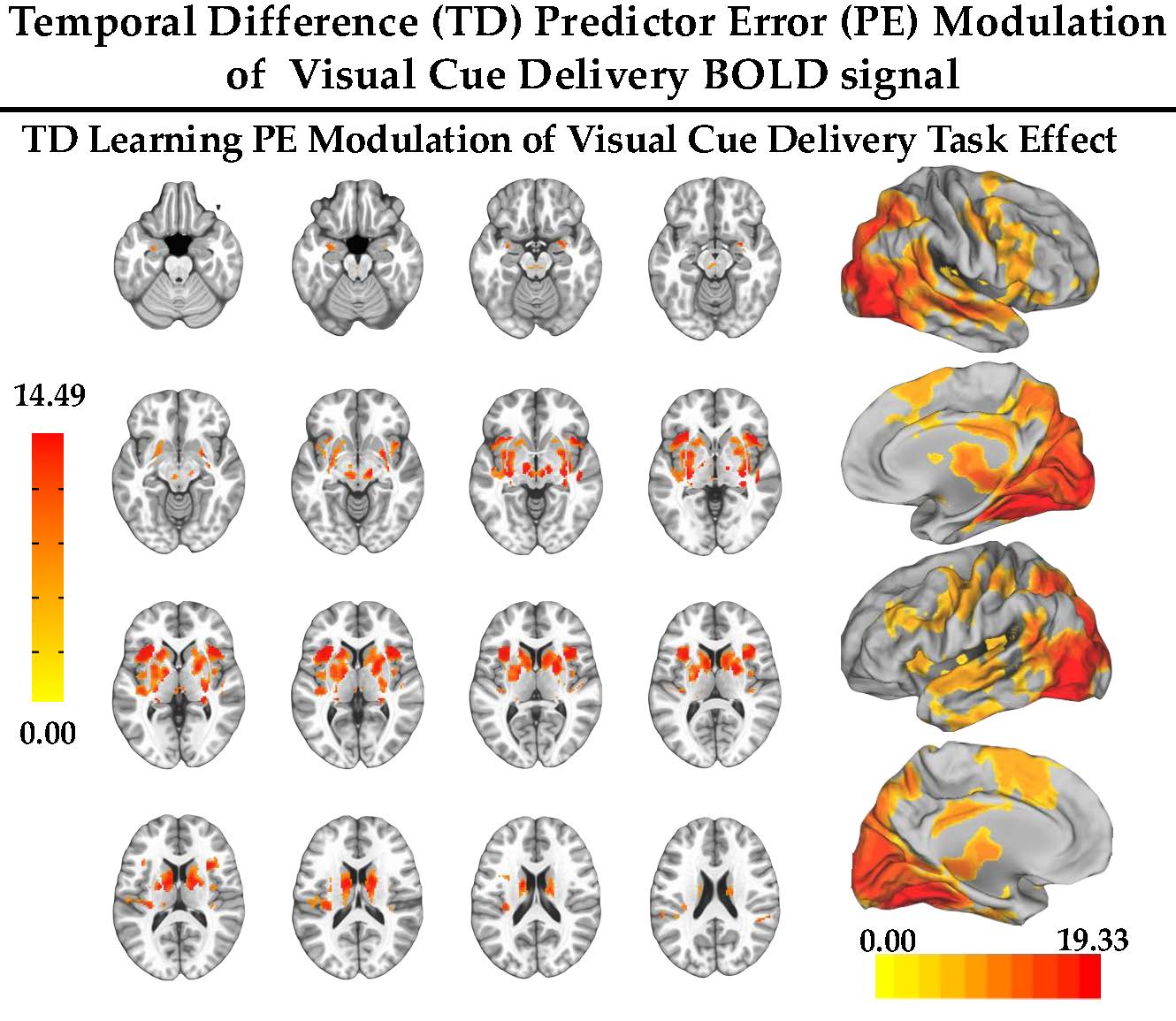


*Supplemental Figure 6 depicts the reward region of interest (ROI)-constrained patterns of activation modulation by temporal difference (TD) learning model prediction errors (PEs) for blood oxygenation level dependent (BOLD) responses to visual cue delivery (brain slice montage displayed on the MNI152 ICBM 2009c non-linear asymmetric average brain) as well as patterns of TD learning PE modulation of BOLD response to visual cue delivery in the whole brain exploratory analysis (projected onto an average brain surface). Color bars indicate the magnitude of the probabilistic threshold-free cluster enhancement (pTFCE)-corrected signed Z values (with positive indicating activation and negative indicating deactivation) displayed on the brain images.*

**Supplemental Table 1. Sample Demographic and Clinical Characteristics**

| Measure/  Characteristic | PTP Sample:  Mean (SD) or N | | | | | | TEHC Sample:  Mean (SD) or N | | | | | | PTP vs. TEHC  Statistic (pval) |
| --- | --- | --- | --- | --- | --- | --- | --- | --- | --- | --- | --- | --- | --- |
| N | 45 | | | | | | 45 | | | | | | -- |
| Age | 27.91 (6.05) | | | | | | 26.18 (6.78) | | | | | | *t* = 1.28 (0.20) |
| Sex Assigned at Birth | N=32 female  N=13 male | | | | | | N=25 female  N=20 male | | | | | | $\mathcal{X}^{2}$=2.34 (0.13) |
| Gender Identity | N=32 female  N=13 male | | | | | | N=25 female  N=20 male | | | | | | $\mathcal{X}^{2}$=2.34 (0.13) |
| Ethnicity | N=14 Hispanic  N=31 non-Hispanic | | | | | | N=12 Hispanic  N=33 non-Hispanic | | | | | | $\mathcal{X}^{2}$=0.22 (0.65) |
| Race | N=27 White  N=5 Black or African American  N=9 Asian  N=4 Native American or Alaskan Native  N=3 Other | | | | | | N=26 White  N=3 Black or African American  N=13 Asian  N=1 Native American or Alaskan Native  N=4 Other | | | | | | $\mathcal{X}^{2}$=3.18 (0.53) |
| Work/Career | N=16 Full-time employment  N=6 Part-time employment  N=8 Unemployed  N=21 Full-time student  N=4 Part-time student | | | | | | N=14 Full-time employment  N=13 Part-time employment  N=3 Unemployed  N=24 Full-time student  N=2 Part-time student | | | | | | $\mathcal{X}^{2}$=5.84 (0.21) |
| WASI Full Scale IQ | 118.16 (12.71) | | | | | | 118.00 (12.96) | | | | | | *t* = 0.06 (0.95) |
| Years of Education | 16.01 (2.63) | | | | | | 16.71 (2.36) | | | | | | *t* = -1.33 (0.19) |
| Diagnoses | N=45 PTSD  N=21 MDD  N=2 PD  N=8 SAD  N=11 GAD | | | | | | -- | | | | | | -- |
| MASQ-GD | 29.54 (9.21) | | | | | | 16.62 (5.30) | | | | | | *t* = 8.01 (<0.001) |
| MASQ-AD | 41.79 (4.65) | | | | | | 28.67 (5.31) | | | | | | *t* = 12.34 (<0.001) |
| MASQ-AA | 19.51 (6.64) | | | | | | 11.87 (2.10) | | | | | | *t* = 7.21 (<0.001) |
| LEC |  | E | W | L | NS | NA |  | E | W | L | NS | NA |  |
|  | Nature | 17 | 11 | 4 | 1 | 12 | Nature | 18 | 4 | 10 | 1 | 12 | $\mathcal{X}^{2}$=5.87 (0.21) |
|  | Fire | 4 | 5 | 12 | 0 | 24 | Fire | 3 | 10 | 9 | 1 | 22 | $\mathcal{X}^{2}$=2.31 (0.68) |
|  | Transport | 24 | 5 | 2 | 0 | 14 | Transport | 21 | 6 | 9 | 0 | 9 | $\mathcal{X}^{2}$=5.83 (0.21) |
|  | Accident | 9 | 9 | 5 | 2 | 20 | Accident | 5 | 5 | 8 | 2 | 25 | $\mathcal{X}^{2}$=3.53 (0.47) |
|  | Toxic | 2 | 0 | 5 | 4 | 34 | Toxic | 1 | 0 | 8 | 2 | 34 | $\mathcal{X}^{2}$=1.69 (0.79) |
|  | Phys Ass | 28 | 5 | 4 | 1 | 7 | Phys Ass | 9 | 7 | 8 | 1 | 20 | $\mathcal{X}^{2}$=17.68 (0.001) |
|  | Weap Ass | 9 | 4 | 15 | 2 | 15 | Weap Ass | 3 | 3 | 12 | 1 | 26 | $\mathcal{X}^{2}$=6.76 (0.15) |
|  | Rape | 22 | 3 | 12 | 1 | 7 | Rape | 5 | 1 | 12 | 1 | 26 | $\mathcal{X}^{2}$=22.64 (<0.001) |
|  | Unw Sex | 32 | 4 | 4 | 0 | 5 | Unw Sex | 9 | 2 | 13 | 1 | 20 | $\mathcal{X}^{2}$=27.33 (<0.001) |
|  | War | 1 | 0 | 8 | 2 | 34 | War | 1 | 0 | 10 | 0 | 34 | $\mathcal{X}^{2}$=0.54 (0.97) |
|  | Captivity | 3 | 0 | 5 | 3 | 34 | Captivity | 1 | 1 | 2 | 2 | 39 | $\mathcal{X}^{2}$=2.82 (0.59) |
|  | Illness/Inj | 5 | 14 | 5 | 2 | 19 | Illness/Inj | 2 | 17 | 7 | 2 | 17 | $\mathcal{X}^{2}$=2.02 (0.73) |
|  | Sev Suff | 5 | 7 | 9 | 7 | 17 | Sev Suff | 1 | 7 | 4 | 4 | 29 | $\mathcal{X}^{2}$=8.54 (0.07) |
|  | Sud Viol D | 2 | 9 | 18 | 1 | 15 | Sud Viol D | 0 | 4 | 14 | 1 | 26 | $\mathcal{X}^{2}$=5.26 (0.26) |
|  | Sud Un D | 16 | 4 | 7 | 2 | 16 | Sud Un D | 14 | 3 | 11 | 0 | 17 | $\mathcal{X}^{2}$=1.50 (0.83) |
|  | Perp Harm | 1 | 2 | 1 | 2 | 39 | Perp Harm | 0 | 1 | 1 | 1 | 42 | $\mathcal{X}^{2}$=0.72 (0.95) |
|  | Other | 20 | 2 | 0 | 6 | 17 | Other | 15 | 0 | 1 | 11 | 18 | $\mathcal{X}^{2}$=2.58 (0.64) |
| CAPS-5 Total | 35.53 (11.04) | | | | | | 3.20 (4.02) | | | | | | *t* = 18.43 (<0.001) |
| CAPS-5 ReExp | 8.02 (4.23) | | | | | | 0.88 (1.35) | | | | | | *t* = 10.76 (<0.001) |
| CAPS-5 Act Avoid | 3.71 (2.03) | | | | | | 0.36 (0.75) | | | | | | *t* = 10.36 (<0.001) |
| CAPS-5 NA | 7.73 (2.78) | | | | | | 0.48 (1.09) | | | | | | *t* = 16.26 (<0.001) |
| CAPS-5 Anhedonia | 6.13 (3.05) | | | | | | 0.36 (0.84) | | | | | | *t* = 12.23 (<0.001) |
| CAPS-5 Ext Behav | 2.07 (1.51) | | | | | | 0.23 (0.60) | | | | | | *t* = 7.56 (<0.001) |
| CAPS-5 Anx Arousal | 3.18 (2.39) | | | | | | 0.41 (0.87) | | | | | | *t* = 7.30 (<0.001) |
| CAPS-5 Dys Arousal | 4.69 (1.46) | | | | | | 0.48 (0.90) | | | | | | *t* = 16.42 (<0.001) |
| CAPS-5 Duration of symptoms (months) | 71.8 (68.56) | | | | | | 1.44 (2.26) | | | | | | *t* = 6.88 (<0.001) |
| CAPS-5 Age of Criterion A event | 21.46 (7.71) | | | | | | 19.78 (5.34) | | | | | | *t =* 1.21 (0.23) |
| SHAPS-C Total | 29.93 (5.44) | | | | | | 26.68 (2.70) | | | | | | *t* = 3.59 (<0.001) |
| WHO-QOL Global | 6.45 (1.37) | | | | | | 8.44 (0.81) | | | | | | *t* = -8.30 (<0.001) |
| WHO-QOL Physical | 12.90 (2.68) | | | | | | 17.30 (1.62) | | | | | | *t* = -9.35 (<0.001) |
| WHO-QOL Psych | 10.53 (2.74) | | | | | | 15.76 (1.51) | | | | | | *t* = -11.13 (<0.001) |
| WHO-QOL Social | 11.88 (3.41) | | | | | | 16.36 (2.00) | | | | | | *t = -*7.53 (<0.001) |
| WHO-QOL Envir | 12.68 (2.69) | | | | | | 16.56 (1.41) | | | | | | *t* = -8.48(<0.001) |
| BDI-II | 21.50 (11.39) | | | | | | 2.58 (3.42) | | | | | | *t* = 10.57 (<0.001) |
| PANAS PA | 22.32 (6.99) | | | | | | 32.80 (6.87) | | | | | | *t* = -7.13 (<0.001) |
| PANAS NA | 22.50 (7.32) | | | | | | 13.27 (2.86) | | | | | | *t* = 7.81 (<0.001) |
| TEPS AP | 3.75 (0.78) | | | | | | 4.51 (0.67) | | | | | | *t* = -4.96 (<0.001) |
| TEPS CP | 4.25 (0.89) | | | | | | 4.80 (0.69) | | | | | | *t* = -3.29 (0.002) |
| CTQ EmAb | 14.30 (5.08) | | | | | | 7.82 (3.60) | | | | | | *t =* 6.92 (<0.001) |
| CTQ PhysAb | 8.32 (3.65) | | | | | | 6.31 (2.10) | | | | | | *t =* 3.17 (0.002) |
| CTQ SexAb | 9.11 (6.02) | | | | | | 6.00 (3.92) | | | | | | *t =* 2.89 (0.005) |
| CTQ EmNeg | 13.95 (4.49) | | | | | | 8.80 (3.51) | | | | | | *t =* 6.03 (<0.001) |
| CTQ PhysNeg | 8.89 (3.19) | | | | | | 5.98 (1.95) | | | | | | *t =* 5.17 (<0.001) |
| CTQ Total | 54.57 (17.11) | | | | | | 34.91 (12.25) | | | | | | *t =* 6.22 (<0.001) |

*AA=anxious arousal; Act Avoid = Active Avoidance; Accident=Serious accident; AD=anhedonic depression; Anx Arousal = anxious arousal; AP=Anticipatory Pleasure; BDI-II=Beck Depression Inventory II; Captivity=Exposure to being held captive; CAPS-5 = Clinician-Administered PTSD Scale for DSM-5; CP=Consummatory Pleasure; CTQ = Childhood Trauma Questionnaire; Dys Arousal = dysphoric arousal; E=Experienced; Envir=Environmental Health; EmAb=emotional abuse; EmNeg=emotional neglect; Ext Behav = externalizing behaviors; GD=General Distress; Illness/Inj=Life-threatening illness or injury; L=Learned about; LEC=Life Events Checklist; MASQ=Mood and Anxiety Symptom Questionnaire; NA=Negative Affect; NA=Not Applicable; Nature = Natural disaster; NS=Not sure; Other=Any other very stressful event or experience; PA=Positive Affect; PANAS=Positive and Negative Affect Scale; Perp Harm=Serious injury, harm, or death perpetrated on another; PhysAb=physical abuse; Phys Ass=Physical assault; PhysNeg=physical neglect; Psych=Psychological Health; ReExp = reexperiencing; Sev Suff=Exposure to severe human suffering; SexAbs=sexual abuse; Social=Social Relationships; SHAPS-C=Clinician-Administered Snaith Hamilton Pleasure Scale; Sud Viol D=Sudden violent death; Sud Un D=Sudden unexpected death of a loved one; TEPS=Temporal Experiences of Pleasure Scale; Toxic=Exposure to toxic substances; Transport=Transportation accident; Unw Sex = Unwanted sexual experience; W=Witnessed; War=Exposure to war zone or combat; Weap Ass=Assault with a weapon; WHO-QOL=World Health Organization Quality of Life Brief Version.*

**Supplemental Table 2. Task Dependent Activation for Juice vs. Non-Juice Predictive Cue**

| **Hem.** | | **Region(s)** | **Voxels** | | **X** | | **Y** | | **Z** | | **Voxel Stats** | | | | |
| --- | --- | --- | --- | --- | --- | --- | --- | --- | --- | --- | --- | --- | --- | --- | --- |
|  |  |  |  |  |  |  |  |  |  |  | ***pTFCE Z*** | | | | |
|  |  |  |  |  |  |  |  |  |  |  | ***Mean*** | | ***SD*** | | |
| **ROI Constrained Analysis** | | | | | | | | | | | | | | |  |
| L/R | Right Insula Lobe/Right Putamen/Right Caudate Nucleus/Right Rolandic Operculum/Right Pallidum/ Right Amygdala/Right Inferior Frontal Gyrus (p. Opercularis)/Right Olfactory cortex/Right Rectal Gyrus/Right Inferior Frontal Gyrus (p. Triangularis)/Left Olfactory cortex/Left Anterior Cingulate Cortex (-) | | 6164 | 29 | | -5 | | 4 | | 10.12 | | 2.613 | |  |  |
| L | Left Insula Lobe/Left Putamen/Left Caudate Nucleus/Left Rolandic Operculum/Left Amygdala/Left Pallidum/Left Inferior Frontal Gyrus (p. Opercularis)/Left Anterior Cingulate Cortex/Left Olfactory cortex/Left Rectal Gyrus/Left Mid Orbital Gyrus//Left Inferior Frontal Gyrus (p. Orbitalis) (-) | | 4923 | -32 | | -9 | | 7 | | 9.96 | | 2.933 | |  |  |
| L | Left Anterior Cingulate Cortex/Left Superior Medial Gyrus (-) | | 9 | -12 | | 50 | | 2 | | 5.62 | | 1.182 | |  |  |
| L | Left Anterior Cingulate Cortex (-) | | 4 | -14 | | 45 | | -5 | | 5.02 | | 0.906 | |  |  |
| L | Left Insula Lobe (-) | | 2 | -31 | | 20 | | 14 | | 4.29 | | 0.000 | |  |  |
| **Whole Brain Analysis** | | | | | | | | | | | | | | |  |
| L/R | Right Middle Frontal Gyrus/Left Postcentral Gyrus/Right Postcentral Gyrus/Left Middle Occipital Gyrus/Left Precentral Gyrus/Left Middle Frontal Gyrus/Right Precentral Gyrus/Left Precuneus/Right Superior Temporal Gyrus/Right Fusiform Gyrus/Left Superior Temporal Gyrus/Right Lingual Gyrus/Left Cerebellum (Crus 1)/Right Middle Temporal Gyrus/Left Calcarine Gyrus/Right Superior Frontal Gyrus/Left Lingual Gyrus/Left Fusiform Gyrus/Right SMA/Right Middle Occipital Gyrus/Right SupraMarginal Gyrus/Left SMA/Left Middle Temporal Gyrus/Right Inferior Temporal Gyrus/Left Superior Parietal Lobule/Left Inferior Parietal Lobule/Left Cerebellum (VI)/Right Calcarine Gyrus/Left Cerebellum (Crus 2)/Right Superior Parietal Lobule/Right Precuneus/Right Insula Lobe/Left Cuneus/Right Cerebellum (VI)/Left Inferior Temporal Gyrus/Left Superior Frontal Gyrus/Left Middle Cingulate Cortex/Right Middle Cingulate Cortex/Right Superior Occipital Gyrus/Left Superior Occipital Gyrus/Right Rolandic Operculum/Right Cuneus/Right Inferior Frontal Gyrus (p. Triangularis)/Left SupraMarginal Gyrus/Right Inferior Parietal Lobule/Right Inferior Frontal Gyrus (p. Opercularis)/Left Insula Lobe/Left Paracentral Lobule/Left Cerebellum (IV-V)/Right Cerebellum (VIII)/Right Putamen/Left Rolandic Operculum/Right ParaHippocampal Gyrus/Right Inferior Occipital Gyrus/Right Cerebellum (Crus 1)/Right Thalamus/Left Thalamus/Left Cerebellum (VIII)/Left Inferior Occipital Gyrus/Right Angular Gyrus/Right Caudate Nucleus/Left ParaHippocampal Gyrus/Left Inferior Frontal Gyrus (p. Triangularis)/Left Putamen/Right Cerebellum (IV-V)/Left Hippocampus/Left Caudate Nucleus/Left Superior Medial Gyrus/Right Temporal Pole/Cerebellar Vermis (4/5)/Right Paracentral Lobule/Left Inferior Frontal Gyrus (p. Opercularis)/Right Cerebellum (Crus 2)/Right Superior Medial Gyrus/Right Hippocampus/Left Cerebellum (VII)/Right Inferior Frontal Gyrus (p. Orbitalis)/Right Medial Temporal Pole/Left Angular Gyrus/Cerebellar Vermis (6)/Left Temporal Pole/Left Inferior Frontal Gyrus (p. Orbitalis)/Right Pallidum/Right Amygdala/Right Cerebellum (IX)/Right Heschls Gyrus/Right Superior Orbital Gyrus/Left Heschls Gyrus/Cerebellar Vermis (3)/Left Anterior Cingulate Cortex/Right Anterior Cingulate Cortex/Left Amygdala/Left Rectal Gyrus/Cerebellar Vermis (8)/Right Middle Orbital Gyrus/Right Rectal Gyrus/Right Posterior Cingulate Cortex/Right Cerebellum (VII)/Left Posterior Cingulate Cortex/Right Cerebellum (III)/Cerebellar Vermis (7)/Left Medial Temporal Pole/Right Olfactory cortex/Left Olfactory cortex/Left Cerebellum (III)/Left Cerebellum (IX)/Left Superior Orbital Gyrus/Cerebellar Vermis (10)/Left Pallidum/Left Middle Orbital Gyrus/Cerebellar Vermis (9)/Cerebellar Vermis (1/2)/Left Mid Orbital Gyrus/Left Cerebellum (X)/Right Cerebellum (X)/Right Mid Orbital Gyrus (-) | | 128347 | 1 | | -36 | | 13 | | 10.22 | | 3.258 | |  |  |
| L | Left Superior Medial Gyrus/Left Anterior Cingulate Cortex/Left Superior Frontal Gyrus/Left Mid Orbital Gyrus (-) | | 48 | -13 | | 50 | | 5 | | 6.07 | | 0.971 | |  |  |
| L | Left Middle Frontal Gyrus/Left Superior Frontal Gyrus/Left Middle Orbital Gyrus/Left Superior Orbital Gyrus (-) | | 30 | -30 | | 48 | | 2 | | 5.41 | | 0.760 | |  |  |
| L | Left Anterior Cingulate Cortex (-) | | 12 | -7 | | 31 | | 13 | | 5.25 | | 0.622 | |  |  |
| L | Left Superior Orbital Gyrus/Left Superior Frontal Gyrus (-) | | 8 | -23 | | 52 | | -1 | | 5.13 | | 0.413 | |  |  |
| R | Right Superior Orbital Gyrus/Right Middle Orbital Gyrus (-) | | 7 | 24 | | 68 | | -9 | | 4.70 | | 0.000 | |  |  |
| L | Left Superior Orbital Gyrus/Left Middle Orbital Gyrus (-) | | 6 | -21 | | 44 | | -11 | | 5.89 | | 0.907 | |  |  |
| R | Right Hippocampus (-) | | 4 | 40 | | -27 | | -10 | | 4.99 | | 0.565 | |  |  |
| R | Right Middle Cingulate Cortex (-) | | 3 | 10 | | -32 | | 30 | | 4.70 | | 0.000 | |  |  |
| R | Right Middle Orbital Gyrus (-) | | 2 | 44 | | 56 | | -11 | | 4.71 | | 0.005 | |  |  |
| L | Left Middle Temporal Gyrus (-) | | 2 | -48 | | -29 | | -10 | | 4.70 | | 0.000 | |  |  |
| R | Right Superior Frontal Gyrus/Right Superior Medial Gyrus (-) | | 2 | 17 | | 46 | | 28 | | 4.70 | | 0.000 | |  |  |

*X, Y, and Z values are cluster center of mass coordinates in MNI stereotactic space; Voxel stats column depicts the mean and standard deviation of the voxelwise statistics for each effect; (-) = deactivation effect.*

**Supplemental Table 3. Task Dependent Activation for Juice vs. Visual Cue Delivery at Expected Times**

| **Hem.** | | **Region(s)** | **Voxels** | | | **X** | | **Y** | | **Z** | | **Voxel Stats** | | | |  |
| --- | --- | --- | --- | --- | --- | --- | --- | --- | --- | --- | --- | --- | --- | --- | --- | --- |
|  |  |  |  |  |  |  |  |  |  |  |  | ***pTFCE Z*** | | | |  |
|  |  |  |  |  |  |  |  |  |  |  |  | ***Mean*** | | ***SD*** | |  |
| **ROI Constrained Analysis** | | | | | | | | | | | | | | | | |
| L/R | Right Insula Lobe/Right Putamen/Left Putamen/Right Pallidum/Left Pallidum/Right Rolandic Operculum/Left Amygdala/Right Inferior Frontal Gyrus (p. Opercularis)/Right Caudate Nucleus/Right Amygdala/Right Inferior Frontal Gyrus (p. Triangularis)/Left Caudate Nucleus/Right ParaHippocampal Gyrus/Left Olfactory cortex/Right Olfactory cortex/Left ParaHippocampal Gyrus/Right Heschls Gyrus/Left Temporal Pole/Right Rectal Gyrus/Left Rectal Gyrus | | | 5067 | 15 | | 0 | | -1 | | 7.94 | | 1.981 | |  |  |
| L | Left Insula Lobe/Left Rolandic Operculum/Left Inferior Frontal Gyrus (p. Opercularis)/Left Inferior Frontal Gyrus (p. Triangularis)/Left Postcentral Gyrus/Left Heschls Gyrus/Left Temporal Pole/Left Putamen/Left Inferior Frontal Gyrus (p. Orbitalis) | | | 1579 | -38 | | 2 | | 5 | | 8.08 | | 1.771 | |  |  |
| R | Right Caudate Nucleus | | | 357 | 20 | | -20 | | 13 | | -9.70 | | 1.803 | |  |  |
| L/R | Left Caudate Nucleus/Right Caudate Nucleus/Right Olfactory cortex/Left Olfactory cortex | | | 288 | 1 | | 19 | | 5 | | -7.40 | | 2.099 | |  |  |
| R | Right Rolandic Operculum/Right Insula Lobe/Right Heschls Gyrus | | | 283 | 37 | | -22 | | 21 | | -7.91 | | 1.499 | |  |  |
| L | Left Superior Temporal Gyrus/Left Rolandic Operculum | | | 128 | -52 | | -34 | | 24 | | 7.57 | | 1.744 | |  |  |
| R | Right Rolandic Operculum/Right Superior Temporal Gyrus | | | 83 | 53 | | -30 | | 23 | | 5.93 | | 1.854 | |  |  |
| L | Left Insula Lobe | | | 20 | -34 | | -31 | | 26 | | -6.22 | | 0.787 | |  |  |
| L | Left Insula Lobe | | | 15 | -29 | | -23 | | 17 | | -5.78 | | 0.954 | |  |  |
| L | Left Anterior Cingulate Cortex/Left Mid Orbital Gyrus/Left Superior Medial Gyrus | | | 10 | -6 | | 53 | | -1 | | -5.16 | | 0.885 | |  |  |
| L | Left Caudate Nucleus | | | 2 | -15 | | 12 | | 18 | | -5.71 | | 0.531 | |  |  |
| **Whole Brain Analysis** | | | | | | | | | | | | | | | | |
| L/R | Left Middle Occipital Gyrus/Right Lingual Gyrus/Left Precentral Gyrus/Left Calcarine Gyrus/Left Lingual Gyrus/Right Fusiform Gyrus/Left Middle Temporal Gyrus/Right Middle Occipital Gyrus/Left Fusiform Gyrus/Right Precentral Gyrus/Right Middle Frontal Gyrus/Right Calcarine Gyrus/Left Middle Frontal Gyrus/Left Inferior Temporal Gyrus/Left Superior Parietal Lobule/Right Cerebellum (Crus 1)/Right Inferior Temporal Gyrus/Left Inferior Parietal Lobule/Left Postcentral Gyrus/Left Cerebellum (Crus 1)/Right Superior Occipital Gyrus/Right Superior Parietal Lobule/Left Superior Occipital Gyrus/Left Cuneus/Right Precuneus/Right Cuneus/Left Precuneus/Right Cerebellum (VI)/Right Middle Temporal Gyrus/Left Cerebellum (IV-V)/Left Cerebellum (VI)/Right Angular Gyrus/Left Inferior Occipital Gyrus/Right Cerebellum (IV-V)/Left Paracentral Lobule/Right Superior Frontal Gyrus/Left Inferior Frontal Gyrus (p. Triangularis)/Right Inferior Occipital Gyrus/Right Postcentral Gyrus/Cerebellar Vermis (4/5)/Left Inferior Frontal Gyrus (p. Orbitalis)/Right Hippocampus/Right ParaHippocampal Gyrus/Right Paracentral Lobule/Left Hippocampus/Left Superior Temporal Gyrus/Right Cerebellum (Crus 2)/Left ParaHippocampal Gyrus/Right Inferior Frontal Gyrus (p. Triangularis)/Left Caudate Nucleus/Right Superior Temporal Gyrus/Left Mid Orbital Gyrus/Left Cerebellum (Crus 2)/Left Superior Frontal Gyrus/Right Inferior Parietal Lobule/Left Angular Gyrus/Right Superior Medial Gyrus/Right Inferior Frontal Gyrus (p. Opercularis)/Left Superior Medial Gyrus/Right Middle Cingulate Cortex/Cerebellar Vermis (6)/Left Temporal Pole/Right Caudate Nucleus/Left Middle Cingulate Cortex/Left Thalamus/Left Rectal Gyrus/Left Middle Orbital Gyrus/Left Inferior Frontal Gyrus (p. Opercularis)/Right Mid Orbital Gyrus/Right SMA/Right Thalamus/Cerebellar Vermis (8)/Left Superior Orbital Gyrus/Right Superior Orbital Gyrus/Cerebellar Vermis (3)/Cerebellar Vermis (7)/Left SMA/Right Middle Orbital Gyrus/Right Rectal Gyrus/Right SupraMarginal Gyrus/Right Rolandic Operculum/Left Medial Temporal Pole/Right Posterior Cingulate Cortex/Left Cerebellum (III)/Left Cerebellum (VIII)/Right Cerebellum (III)/Left Posterior Cingulate Cortex/Right Insula Lobe/Right Heschls Gyrus/Left Cerebellum (VII)/Right Cerebellum (VIII)/Right Cerebellum (VII)/Left Cerebellum (IX)/Left Anterior Cingulate Cortex/Left SupraMarginal Gyrus/Cerebellar Vermis (9)/Cerebellar Vermis (10)/Cerebellar Vermis (1/2)/Right Cerebellum (IX)/Right Olfactory cortex/Left Insula Lobe/Left Olfactory cortex | | | 66042 | 0 | | -53 | | 11 | | -11.31 | | 4.429 | |  |  |
| L/R | Left Insula Lobe/Right Insula Lobe/Right SupraMarginal Gyrus/Left Postcentral Gyrus/Right Putamen/Right Postcentral Gyrus/Right Rolandic Operculum/Left SupraMarginal Gyrus/Left Putamen/Left Rolandic Operculum/Right Inferior Frontal Gyrus (p. Opercularis)/Right Precentral Gyrus/Left Inferior Frontal Gyrus (p. Opercularis)/Left Superior Temporal Gyrus/Left Precentral Gyrus/Right Thalamus/Right Pallidum/Right Inferior Frontal Gyrus (p. Triangularis)/Left Thalamus/Left Pallidum/Right Superior Temporal Gyrus/Left Amygdala/Left Inferior Parietal Lobule/Left Inferior Frontal Gyrus (p. Triangularis)/Right Amygdala/Left Temporal Pole/Right Caudate Nucleus/Right Inferior Frontal Gyrus (p. Orbitalis)/Right Olfactory cortex/Left Heschls Gyrus/Right Inferior Parietal Lobule/Left Olfactory cortex/Left Inferior Frontal Gyrus (p. Orbitalis)/Right Hippocampus/Left Hippocampus/Right Temporal Pole/Right Heschls Gyrus/Left ParaHippocampal Gyrus/Right ParaHippocampal Gyrus/Right Rectal Gyrus/Left Caudate Nucleus/Right Middle Frontal Gyrus/Left Superior Orbital Gyrus/Left Middle Temporal Gyrus/Right Superior Orbital Gyrus/Left Rectal Gyrus/Right Angular Gyrus | | | 15761 | 3 | | -5 | | 11 | | 8.17 | | 1.913 | |  |  |
| R | Right Middle Frontal Gyrus/Right Middle Orbital Gyrus/Right Superior Frontal Gyrus/Right Superior Orbital Gyrus | | | 173 | 29 | | 51 | | 1 | | -5.91 | | 0.822 | |  |  |
| L | Left Middle Frontal Gyrus/Left Superior Frontal Gyrus/Left Superior Orbital Gyrus | | | 93 | -25 | | 51 | | 10 | | -6.29 | | 0.728 | |  |  |
| R | Right Inferior Occipital Gyrus/Right Lingual Gyrus | | | 68 | 30 | | -95 | | -8 | | 5.45 | | 0.534 | |  |  |
| L | Left Middle Temporal Gyrus | | | 54 | -58 | | -63 | | 8 | | 4.63 | | 0.071 | |  |  |
| R | Right Middle Temporal Gyrus/Right Inferior Temporal Gyrus/Right Fusiform Gyrus | | | 53 | 51 | | -10 | | -19 | | -5.94 | | 0.510 | |  |  |
| R | Right Inferior Frontal Gyrus (p. Orbitalis)/Right Temporal Pole | | | 47 | 42 | | 28 | | -19 | | -4.57 | | 0.048 | |  |  |
| L | Left Middle Cingulate Cortex | | | 25 | -12 | | -9 | | 43 | | -6.33 | | 1.090 | |  |  |
| R | Right Middle Cingulate Cortex/Right Anterior Cingulate Cortex | | | 20 | 14 | | 31 | | 32 | | -5.81 | | 0.556 | |  |  |
| L | Left Middle Cingulate Cortex | | | 19 | -12 | | 11 | | 36 | | -6.81 | | 1.499 | |  |  |
| R | Right Postcentral Gyrus/Right Inferior Parietal Lobule | | | 17 | 40 | | -37 | | 59 | | 4.66 | | 0.072 | |  |  |
| R | Right Middle Cingulate Cortex/Right SMA | | | 16 | 14 | | -5 | | 41 | | -6.03 | | 0.870 | |  |  |
| R | Right Inferior Frontal Gyrus (p. Orbitalis)/Right Temporal Pole | | | 11 | 48 | | 27 | | -16 | | -4.55 | | 0.035 | |  |  |
| L | Left SupraMarginal Gyrus | | | 11 | -49 | | -30 | | 32 | | -5.87 | | 0.505 | |  |  |
| R | Right Medial Temporal Pole/Right Temporal Pole | | | 8 | 48 | | 20 | | -31 | | -4.53 | | 0.024 | |  |  |
| L | Left Superior Frontal Gyrus | | | 6 | -19 | | 44 | | 49 | | -4.52 | | 0.000 | |  |  |
| L | Left Middle Temporal Gyrus/Left Inferior Temporal Gyrus | | | 5 | -48 | | -20 | | -16 | | -5.50 | | 0.427 | |  |  |
| L | Left Insula Lobe | | | 4 | -30 | | -22 | | 17 | | -5.29 | | 0.001 | |  |  |
| L | Left Anterior Cingulate Cortex | | | 4 | -12 | | 29 | | 24 | | -5.29 | | 0.001 | |  |  |
| R | Right Middle Orbital Gyrus | | | 3 | 47 | | 51 | | -14 | | -4.52 | | 0.001 | |  |  |
| R | Right Posterior Cingulate Cortex | | | 3 | 10 | | -43 | | 23 | | -5.29 | | 0.001 | |  |  |
| R | Right Middle Cingulate Cortex | | | 3 | 12 | | 27 | | 39 | | -5.29 | | 0.001 | |  |  |
| R | Right Temporal Pole | | | 2 | 48 | | 22 | | -27 | | -4.59 | | 0.001 | |  |  |
| L | Left Middle Temporal Gyrus | | | 2 | -46 | | -17 | | -14 | | -5.82 | | 0.534 | |  |  |
| L | Left Middle Temporal Gyrus | | | 2 | -48 | | -25 | | -12 | | -5.29 | | 0.001 | |  |  |
| R | Right Angular Gyrus | | | 2 | 42 | | -47 | | 34 | | -5.29 | | 0.001 | |  |  |
| R | Right Superior Frontal Gyrus | | | 2 | 18 | | 35 | | 36 | | -5.29 | | 0.001 | |  |  |
| R | Right Superior Frontal Gyrus | | | 2 | 18 | | 29 | | 40 | | -5.29 | | 0.001 | |  |  |
| L | Left Superior Frontal Gyrus | | | 2 | -30 | | -7 | | 70 | | -6.79 | | 0.432 | |  |  |

*X, Y, and Z values are cluster center of mass coordinates in MNI stereotactic space; Voxel stats column depicts the mean and standard deviation of the voxelwise statistics for each effect.*

**Supplemental Table 4. Task Dependent Activation for Juice vs. Visual Cue Positive Temporal Prediction Errors (PEs)**

| **Hem.** | | **Region(s)** | **Voxels** | | | **X** | | **Y** | | **Z** | | **Voxel Stats** | | | |  |
| --- | --- | --- | --- | --- | --- | --- | --- | --- | --- | --- | --- | --- | --- | --- | --- | --- |
|  |  |  |  |  |  |  |  |  |  |  |  | ***pTFCE Z*** | | | |  |
|  |  |  |  |  |  |  |  |  |  |  |  | ***Mean*** | | ***SD*** | |  |
| **ROI Constrained Analysis** | | | | | | | | | | | | | | | | |
| R | Right Insula Lobe/Right Rolandic Operculum/Right Heschls Gyrus/Right Putamen | | | 495 | 43 | | -4 | | 6 | | 6.37 | | 1.043 | |  |  |
| L | Left Insula Lobe/Left Rolandic Operculum/Left Heschls Gyrus/Left Temporal Pole | | | 383 | -41 | | -6 | | 5 | | 5.74 | | 0.718 | |  |  |
| R | Right Caudate Nucleus | | | 374 | 15 | | -10 | | 18 | | 6.27 | | 0.834 | |  |  |
| L | Left Caudate Nucleus | | | 238 | -14 | | -11 | | 18 | | 6.28 | | 0.923 | |  |  |
| R | Right Pallidum/Right Putamen | | | 71 | 27 | | -13 | | -3 | | 4.91 | | 0.528 | |  |  |
| L | Left Caudate | | | 65 | -24 | | -35 | | 6 | | 5.64 | | 0.450 | |  |  |
| R | Right Insula Lobe/Right Inferior Frontal Gyrus (p. Opercularis) | | | 35 | 36 | | 6 | | 16 | | 5.35 | | 0.707 | |  |  |
| L | Left Putamen/Left Pallidum | | | 33 | -29 | | -16 | | -5 | | 4.22 | | 0.025 | |  |  |
| R | Right Insula Lobe | | | 25 | 29 | | -23 | | 19 | | 5.66 | | 0.915 | |  |  |
| R | Right Superior Temporal Gyrus/Right Rolandic Operculum | | | 8 | 51 | | -25 | | 16 | | 5.08 | | 0.427 | |  |  |
| R | Right Caudate | | | 4 | 32 | | -31 | | -1 | | 4.92 | | 0.440 | |  |  |
| R | Right Insula Lobe | | | 4 | 32 | | -23 | | 24 | | 5.46 | | 1.001 | |  |  |
| L | Left Caudate Nucleus | | | 2 | -16 | | 9 | | 20 | | 5.80 | | 0.550 | |  |  |
| **Whole Brain Analysis** | | | | | | | | | | | | | | | | |
| L/R | Left Middle Occipital Gyrus/Left Cerebellum (VI)/Right Lingual Gyrus/Right Middle Occipital Gyrus/Left Lingual Gyrus/Left Calcarine Gyrus/Right Precuneus/Right Middle Temporal Gyrus/Right Cerebellum (VI)/Left Cerebellum (Crus 1)/Right Inferior Occipital Gyrus/Right Angular Gyrus/Right Inferior Temporal Gyrus/Right Calcarine Gyrus/Right Cerebellum (Crus 1)/Right Fusiform Gyrus/Right Inferior Parietal Lobule/Left Cerebellum (IV-V)/Left Superior Occipital Gyrus/Left Cuneus/Right Superior Parietal Lobule/Left Fusiform Gyrus/Right Cuneus/Left Cerebellum (Crus 2)/Cerebellar Vermis (4/5)/Left Precuneus/Right Cerebellum (IV-V)/Right Superior Occipital Gyrus/Left Inferior Occipital Gyrus/Cerebellar Vermis (6)/Right Thalamus/Left Hippocampus/Left Cerebellum (VII)/Cerebellar Vermis (7)/Right Hippocampus/Left Cerebellum (VIII)/Right Posterior Cingulate Cortex/Cerebellar Vermis (3)/Right Cerebellum (III)/Right Middle Cingulate Cortex/Left Inferior Temporal Gyrus/Right Superior Temporal Gyrus/Right ParaHippocampal Gyrus/Left Posterior Cingulate Cortex/Left ParaHippocampal Gyrus/Right Cerebellum (Crus 2)/Right SupraMarginal Gyrus/Right Postcentral Gyrus/Left Cerebellum (X)/Left Thalamus/Right Paracentral Lobule/Left Superior Parietal Lobule | | | 15816 | 8 | | -73 | | 1 | | 6.09 | | 0.925 | |  |  |
| R | Right Precentral Gyrus/Right Postcentral Gyrus/Right Middle Frontal Gyrus/Right Superior Temporal Gyrus/Right Rolandic Operculum/Right Insula Lobe/Right SupraMarginal Gyrus/Right Inferior Frontal Gyrus (p. Triangularis)/Right Inferior Frontal Gyrus (p. Opercularis)/Right Superior Frontal Gyrus/Right Temporal Pole/Right Heschls Gyrus/Right Middle Temporal Gyrus/Right Putamen/Right Inferior Frontal Gyrus (p. Orbitalis) | | | 6121 | 52 | | -2 | | 27 | | 6.52 | | 1.131 | |  |  |
| L | Left Postcentral Gyrus/Left Superior Temporal Gyrus/Left Precentral Gyrus/Left Rolandic Operculum/Left Insula Lobe/Left Middle Temporal Gyrus/Left Heschls Gyrus/Left Inferior Temporal Gyrus/Left SupraMarginal Gyrus/Left Temporal Pole/Left Inferior Parietal Lobule/Left Inferior Frontal Gyrus (p. Opercularis)/Left Middle Frontal Gyrus/Left Putamen | | | 3240 | -53 | | -12 | | 22 | | 6.24 | | 0.997 | |  |  |
| L/R | Right Thalamus/Right Caudate Nucleus/Left Thalamus/Left Caudate Nucleus | | | 1271 | 3 | | -10 | | 16 | | 6.38 | | 0.739 | |  |  |
| R | Right Superior Frontal Gyrus/Right SMA/Right Precentral Gyrus | | | 149 | 19 | | -11 | | 62 | | 4.75 | | 0.215 | |  |  |
| L | Left SMA/Left Superior Frontal Gyrus/Left Middle Cingulate Cortex/Left Middle Frontal Gyrus | | | 122 | -14 | | 3 | | 56 | | 5.33 | | 0.442 | |  |  |
| L | Left Middle Temporal Gyrus/Left Inferior Temporal Gyrus | | | 109 | -48 | | -53 | | 2 | | 5.34 | | 0.470 | |  |  |
| R | Right Superior Frontal Gyrus/Right Middle Frontal Gyrus/Right Inferior Frontal Gyrus (p. Opercularis) | | | 68 | 26 | | 19 | | 40 | | 5.48 | | 0.645 | |  |  |
| L | Left Anterior Cingulate Cortex/Left Middle Cingulate Cortex/Left Superior Frontal Gyrus | | | 54 | -9 | | 11 | | 33 | | 5.76 | | 0.651 | |  |  |
| L/R | Left Cerebellum (VIII)/Cerebellar Vermis (8)/Left Cerebellum (VII) | | | 52 | -3 | | -70 | | -45 | | 4.63 | | 0.053 | |  |  |
| L | Left Middle Cingulate Cortex/Left SMA | | | 51 | -12 | | -8 | | 44 | | 5.71 | | 0.666 | |  |  |
| L | Left Middle Temporal Gyrus/Left Superior Temporal Gyrus/Left Inferior Temporal Gyrus | | | 45 | -46 | | -13 | | -16 | | 5.02 | | 0.142 | |  |  |
| R | Right Hippocampus/Right ParaHippocampal Gyrus/Right Fusiform Gyrus | | | 45 | 34 | | -37 | | -3 | | 5.34 | | 0.352 | |  |  |
| R | Right Pallidum/Right Putamen | | | 35 | 29 | | -12 | | -3 | | 5.28 | | 0.306 | |  |  |
| R | Right Lingual Gyrus/Right Fusiform Gyrus | | | 34 | 28 | | -61 | | -2 | | 5.28 | | 0.321 | |  |  |
| L | Left Precentral Gyrus/Left Middle Frontal Gyrus/Left Superior Frontal Gyrus | | | 33 | -28 | | -2 | | 46 | | 5.18 | | 0.416 | |  |  |
| R | Right Middle Orbital Gyrus/Right Superior Orbital Gyrus | | | 25 | 29 | | 62 | | -11 | | 4.75 | | 0.152 | |  |  |
| R | Right Superior Occipital Gyrus/Right Cuneus | | | 22 | 23 | | -90 | | 35 | | 4.99 | | 0.250 | |  |  |
| L | Left Superior Temporal Gyrus/Left Hippocampus | | | 21 | -39 | | -20 | | -7 | | 4.90 | | 0.160 | |  |  |
| R | Right Insula Lobe/Right Inferior Frontal Gyrus (p. Opercularis) | | | 21 | 36 | | 6 | | 15 | | 5.66 | | 0.579 | |  |  |
| R | Right Cerebellum (VIII) | | | 17 | 14 | | -69 | | -46 | | 4.60 | | 0.033 | |  |  |
| R | Right Superior Parietal Lobule | | | 16 | 22 | | -61 | | 69 | | 5.24 | | 0.193 | |  |  |
| R | Right Middle Cingulate Cortex/Right Posterior Cingulate Cortex | | | 14 | 7 | | -31 | | 28 | | 4.96 | | 0.105 | |  |  |
| L | Left Superior Medial Gyrus/Left Anterior Cingulate Cortex | | | 13 | -11 | | 37 | | 26 | | 4.59 | | 0.025 | |  |  |
| L | Left Precentral Gyrus/Left Middle Frontal Gyrus/Left Inferior Frontal Gyrus (p. Opercularis) | | | 13 | -33 | | 7 | | 32 | | 5.40 | | 0.523 | |  |  |
| L | Left Middle Frontal Gyrus/Left Superior Frontal Gyrus | | | 13 | -24 | | 13 | | 42 | | 5.11 | | 0.384 | |  |  |
| R | Right Superior Orbital Gyrus | | | 9 | 21 | | 33 | | -17 | | 4.69 | | 0.065 | |  |  |
| L | Left Inferior Temporal Gyrus/Left Middle Temporal Gyrus | | | 8 | -45 | | -24 | | -18 | | 5.00 | | 0.108 | |  |  |
| L | Left Middle Temporal Gyrus | | | 8 | -39 | | -58 | | 14 | | 4.66 | | 0.010 | |  |  |
| R | Right Middle Cingulate Cortex | | | 6 | 14 | | 24 | | 36 | | 5.22 | | 0.405 | |  |  |
| R | Right Inferior Parietal Lobule/Right Postcentral Gyrus/Right SupraMarginal Gyrus | | | 6 | 58 | | -29 | | 54 | | 4.95 | | 0.119 | |  |  |
| L | Left Inferior Temporal Gyrus | | | 5 | -44 | | -18 | | -20 | | 5.07 | | 0.050 | |  |  |
| L | Left Cerebellum (VII) | | | 4 | 1 | | -77 | | -42 | | 4.59 | | 0.037 | |  |  |
| R | Right Hippocampus | | | 4 | 23 | | -32 | | 12 | | 5.95 | | 0.604 | |  |  |
| L | Left Superior Frontal Gyrus | | | 4 | -20 | | -9 | | 52 | | 4.83 | | 0.207 | |  |  |
| R | Right Fusiform Gyrus | | | 3 | 32 | | -52 | | -2 | | 5.29 | | 0.323 | |  |  |
| R | Right Anterior Cingulate Cortex | | | 3 | 8 | | 15 | | 25 | | 5.50 | | 0.551 | |  |  |
| R | Right Postcentral Gyrus/Right Superior Parietal Lobule | | | 3 | 50 | | -36 | | 60 | | 5.18 | | 0.280 | |  |  |
| L | Left Inferior Temporal Gyrus | | | 2 | -40 | | -17 | | -18 | | 4.84 | | 0.197 | |  |  |
| L | Left Inferior Temporal Gyrus | | | 2 | -42 | | -25 | | -16 | | 5.02 | | 0.018 | |  |  |
| R | Right Fusiform Gyrus | | | 2 | 32 | | -63 | | -6 | | 4.83 | | 0.178 | |  |  |
| R | Right Hippocampus | | | 2 | 24 | | -35 | | 10 | | 5.16 | | 0.155 | |  |  |
| L | Left Superior Temporal Gyrus | | | 2 | -42 | | -45 | | 14 | | 5.72 | | 0.133 | |  |  |
| L | Left Caudate Nucleus | | | 2 | -18 | | 15 | | 16 | | 5.00 | | 0.001 | |  |  |
| L | Left Superior Occipital Gyrus | | | 2 | -26 | | -92 | | 31 | | 4.65 | | 0.000 | |  |  |
| L | Left Middle Cingulate Cortex | | | 2 | -7 | | -6 | | 34 | | 4.65 | | 0.000 | |  |  |
| R | Right Cuneus | | | 2 | 17 | | -88 | | 42 | | 4.65 | | 0.000 | |  |  |
| R | Right Superior Parietal Lobule | | | 2 | 48 | | -43 | | 60 | | 4.98 | | 0.333 | |  |  |

*X, Y, and Z values are cluster center of mass coordinates in MNI stereotactic space; Voxel stats column depicts the mean and standard deviation of the voxelwise statistics for each effect.*

**Supplemental Table 5. PTP vs. TEHC Abnormalities for Juice vs. Visual Cue Positive Temporal Prediction Errors (PEs)**

| **Hem.** | | **Region(s)** | **Voxels** | | **X** | **Y** | **Z** | | **Voxel Stats** | | | | | **Extracted Cluster Values** | | | | | | |  |  |
| --- | --- | --- | --- | --- | --- | --- | --- | --- | --- | --- | --- | --- | --- | --- | --- | --- | --- | --- | --- | --- | --- | --- |
|  |  |  |  |  |  |  |  |  | ***pTFCE Z*** | | | | | ***Juice Positive Temporal PE*** | | | ***Visual Cue Positive Temporal PE*** | | | |  |  |
|  |  |  |  |  |  |  |  |  | ***Mean*** | | ***SD*** | | | ***PTP*** | ***TEHC*** | | ***PTP*** | | ***TEHC*** | |  |  |
| **ROI Constrained Analysis** | | | | | | | | | | | | | | | | | | | | | | |
| L | Left Pallidum/Left Putamen (-) | | | 22 | -26 | -14 | | -3 | | 4.06 | | 0.082 | 0.008 | | | 0.060 | | 0.004 | | -0.056 | |  |
| L | Left Amygdala (-) | | | 14 | -19 | -8 | | -16 | | 4.00 | | 0.032 | -0.096 | | | 0.085 | | 0.027 | | 0.021 | |  |
| L | Midbrain (-) | | | 13 | -9 | -15 | | -13 | | 4.04 | | 0.055 | -0.020 | | | 0.074 | | 0.096 | | 0.041 | |  |

*X, Y, and Z values are cluster center of mass coordinates in MNI stereotactic space; Voxel stats column depicts the mean and standard deviation of the voxelwise statistics for each effect; Extracted Cluster Values columns list the mean within-subject average % signal changes within identified effect clusters for each group for each of the two constituent conditions composing the juice vs. visual positive temporal PE contrast effect: unexpected vs. expected receipt of juice (juice positive temporal PE) and unexpected vs. expected receipt of visual cue (visual cue positive temporal PE); (-) sign indicates decreased activation for the post-trauma psychopathology (PTP) vs. trauma-exposed healthy comparison (TEHC) groups.*

**Supplemental Table 6. Task Dependent Deactivation for Juice vs. Visual Cue Negative Temporal Prediction Errors (PEs)**

| **Hem.** | | **Region(s)** | **Voxels** | | | **X** | | **Y** | | **Z** | | **Voxel Stats** | | | |  |
| --- | --- | --- | --- | --- | --- | --- | --- | --- | --- | --- | --- | --- | --- | --- | --- | --- |
|  |  |  |  |  |  |  |  |  |  |  |  | ***pTFCE Z*** | | | |  |
|  |  |  |  |  |  |  |  |  |  |  |  | ***Mean*** | | ***SD*** | |  |
| **ROI Constrained Analysis** | | | | | | | | | | | | | | | | |
| R | Right Insula Lobe/Right Rolandic Operculum/Right Inferior Frontal Gyrus (p. Opercularis)/Right Heschls Gyrus/Right Putamen (-) | | | 950 | 41 | | -3 | | 9 | | 7.96 | | 1.755 | |  |  |
| L | Left Caudate Nucleus/Left Amygdala/Left Putamen/Left ParaHippocampal Gyrus/Left Pallidum (-) | | | 913 | -20 | | -11 | | 3 | | 7.21 | | 1.599 | |  |  |
| L | Left Insula Lobe/Left Rolandic Operculum/Left Inferior Frontal Gyrus (p. Opercularis)/Left Heschls Gyrus (-) | | | 872 | -40 | | -9 | | 13 | | 7.84 | | 1.731 | |  |  |
| L/R | Right Caudate Nucleus/Right Olfactory cortex/Left Caudate Nucleus (-) | | | 629 | 15 | | -6 | | 12 | | 7.71 | | 1.605 | |  |  |
| R | Right Amygdala/Right ParaHippocampal Gyrus/Right Putamen/Right Pallidum (-) | | | 526 | 23 | | -5 | | -18 | | 7.24 | | 1.511 | |  |  |
| L/R | Midbrain (-) | | | 225 | -4 | | -11 | | -13 | | 6.56 | | 1.385 | |  |  |
| R | Right Putamen (-) | | | 73 | 39 | | -24 | | -2 | | 7.20 | | 1.238 | |  |  |
| L | Left ParaHippocampal Gyrus/Left Amygdala (-) | | | 53 | -28 | | -3 | | -27 | | 5.60 | | 1.065 | |  |  |
| R | Midbrain (-) | | | 53 | 11 | | -23 | | -12 | | 7.72 | | 1.869 | |  |  |
| L | Left Anterior Cingulate Cortex/Left Mid Orbital Gyrus/Left Superior Medial Gyrus (-) | | | 43 | -12 | | 47 | | -2 | | 6.86 | | 1.395 | |  |  |
| R | Right Putamen (-) | | | 30 | 24 | | -14 | | 10 | | 5.83 | | 0.887 | |  |  |
| L | Left Inferior Frontal Gyrus (p. Triangularis)/Left Insula Lobe/Left Inferior Frontal Gyrus (p. Opercularis) (-) | | | 24 | -34 | | 24 | | 14 | | 6.37 | | 1.202 | |  |  |
| L | Left Insula Lobe (-) | | | 21 | -28 | | -24 | | 20 | | 6.76 | | 1.354 | |  |  |
| R | Right Putamen/Right Amygdala/Right Olfactory cortex (-) | | | 13 | 22 | | 4 | | -10 | | 5.55 | | 0.552 | |  |  |
| L | Left Amygdala/Left ParaHippocampal Gyrus (-) | | | 4 | -23 | | 2 | | -22 | | 4.83 | | 0.343 | |  |  |
| R | Midbrain (-) | | | 4 | 5 | | -22 | | -18 | | 4.68 | | 0.297 | |  |  |
| R | Putamen (-) | | | 3 | 25 | | -9 | | 14 | | 5.25 | | 0.455 | |  |  |
| L | Left Amygdala/Left Temporal Pole (-) | | | 2 | -27 | | 2 | | -24 | | 4.53 | | 0.000 | |  |  |
| R | Right Putamen (-) | | | 2 | 30 | | -12 | | 9 | | 4.53 | | 0.000 | |  |  |
| L | Left Caudate Nucleus (-) | | | 2 | -8 | | 9 | | 16 | | 4.83 | | 0.297 | |  |  |
| **Whole Brain Analysis** | | | | | | | | | | | | | | | | |
| L/R | Left Postcentral Gyrus/Right Postcentral Gyrus/Left Middle Occipital Gyrus/Right Precentral Gyrus/Left Middle Frontal Gyrus/Right Middle Frontal Gyrus/Left Precentral Gyrus/Right Fusiform Gyrus/Left Inferior Parietal Lobule/Right Lingual Gyrus/Right Superior Parietal Lobule/Right Superior Frontal Gyrus/Right Middle Occipital Gyrus/Left Superior Parietal Lobule/Left Precuneus/Left Superior Frontal Gyrus/Right Cerebellum (VI)/Left Lingual Gyrus/Right Precuneus/Left Fusiform Gyrus/Left Calcarine Gyrus/Left Cerebellum (VI)/Right Calcarine Gyrus/Left Inferior Temporal Gyrus/Left Superior Occipital Gyrus/Right Cuneus/Right Inferior Temporal Gyrus/Left Cuneus/Right Superior Occipital Gyrus/Right Middle Temporal Gyrus/Left Inferior Frontal Gyrus (p. Triangularis)/Right SupraMarginal Gyrus/Right Inferior Parietal Lobule/Right Rolandic Operculum/Right Angular Gyrus/Left Cerebellum (IV-V)/Left Paracentral Lobule/Left Cerebellum (Crus 1)/Right Cerebellum (Crus 2)/Right Superior Temporal Gyrus/Right SMA/Right ParaHippocampal Gyrus/Right Cerebellum (IV-V)/Right Insula Lobe/Left SMA/Left Middle Temporal Gyrus/Right Inferior Frontal Gyrus (p. Triangularis)/Right Hippocampus/Right Cerebellum (Crus 1)/Left Rolandic Operculum/Right Inferior Frontal Gyrus (p. Opercularis)/Left SupraMarginal Gyrus/Left Cerebellum (Crus 2)/Left Middle Cingulate Cortex/Left Superior Temporal Gyrus/Cerebellar Vermis (4/5)/Right Inferior Occipital Gyrus/Left Superior Medial Gyrus/Left Insula Lobe/Left Inferior Occipital Gyrus/Right Thalamus/Left Hippocampus/Left Angular Gyrus/Left Thalamus/Right Paracentral Lobule/Right Middle Cingulate Cortex/Left Inferior Frontal Gyrus (p. Opercularis)/Cerebellar Vermis (6)/Left ParaHippocampal Gyrus/Right Middle Orbital Gyrus/Left Caudate Nucleus/Right Caudate Nucleus/Right Temporal Pole/Left Cerebellum (VIII)/Right Amygdala/Left Temporal Pole/Right Inferior Frontal Gyrus (p. Orbitalis)/Left Anterior Cingulate Cortex/Right Superior Orbital Gyrus/Left Superior Orbital Gyrus/Right Superior Medial Gyrus/Right Cerebellum (III)/Cerebellar Vermis (3)/Left Mid Orbital Gyrus/Left Posterior Cingulate Cortex/Left Cerebellum (X)/Left Middle Orbital Gyrus/Left Heschls Gyrus/Right Cerebellum (VIII)/Right Medial Temporal Pole/Left Cerebellum (III)/Right Cerebellum (X)/Right Putamen/Left Amygdala/Right Olfactory cortex/Right Heschls Gyrus/Right Mid Orbital Gyrus/Cerebellar Vermis (1/2)/Right Posterior Cingulate Cortex/Left Putamen/Left Cerebellum (VII)/Cerebellar Vermis (7)/Left Olfactory cortex/Left Cerebellum (IX)/Left Medial Temporal Pole/Right Anterior Cingulate Cortex/Right Cerebellum (VII)/Left Rectal Gyrus/Left Inferior Frontal Gyrus (p. Orbitalis)/Cerebellar Vermis (8)/Right Rectal Gyrus/Left Pallidum/Right Cerebellum (IX)/Right Pallidum/Cerebellar Vermis (10) (-) | | | 81097 | 2 | | -33 | | 18 | | 7.86 | | 1.619 | |  |  |
| L | Left Superior Temporal Gyrus/Left Middle Temporal Gyrus/Left SupraMarginal Gyrus/Left Angular Gyrus (-) | | | 176 | -49 | | -43 | | 16 | | 6.85 | | 1.231 | |  |  |
| L/R | Right Middle Cingulate Cortex/Left Middle Cingulate Cortex/Right Anterior Cingulate Cortex/Left Anterior Cingulate Cortex/Right Superior Frontal Gyrus/Right SMA (-) | | | 132 | 5 | | 11 | | 36 | | 6.26 | | 1.043 | |  |  |
| L/R | Left Anterior Cingulate Cortex/Right Anterior Cingulate Cortex (-) | | | 91 | 0 | | 27 | | 15 | | 6.41 | | 1.040 | |  |  |
| L | Left Middle Temporal Gyrus (-) | | | 61 | -65 | | -55 | | 4 | | 5.79 | | 0.361 | |  |  |
| L | Left Cerebellum (IX) (-) | | | 48 | -1 | | -57 | | -59 | | 5.12 | | 0.026 | |  |  |
| L | Left Middle Temporal Gyrus (-) | | | 46 | -69 | | -34 | | 1 | | 5.77 | | 0.373 | |  |  |
| L | Left Middle Orbital Gyrus/Left Inferior Frontal Gyrus (p. Orbitalis)/Left Superior Orbital Gyrus (-) | | | 33 | -27 | | 38 | | -10 | | 6.54 | | 1.067 | |  |  |
| L | Left Cerebellum (IX) (-) | | | 27 | -9 | | -39 | | -59 | | 5.12 | | 0.021 | |  |  |
| R | Right Insula Lobe/Right Heschls Gyrus (-) | | | 22 | 32 | | -26 | | 15 | | 7.08 | | 1.241 | |  |  |
| R | Right Inferior Frontal Gyrus (p. Orbitalis)/Right Superior Orbital Gyrus/Right Putamen/Right Insula Lobe (-) | | | 20 | 24 | | 28 | | -11 | | 6.05 | | 0.952 | |  |  |
| L | Left Middle Cingulate Cortex (-) | | | 14 | -14 | | -14 | | 43 | | 7.61 | | 0.925 | |  |  |
| L | Left Inferior Temporal Gyrus (-) | | | 13 | -50 | | 10 | | -41 | | 4.59 | | 0.027 | |  |  |
| L | Left Insula Lobe/Left Inferior Frontal Gyrus (p. Triangularis) (-) | | | 6 | -30 | | 30 | | 7 | | 7.03 | | 1.084 | |  |  |
| L | Left Middle Orbital Gyrus/Left Superior Orbital Gyrus (-) | | | 3 | -15 | | 61 | | -18 | | 4.55 | | 0.003 | |  |  |
| R | Right Anterior Cingulate Cortex (-) | | | 3 | 5 | | 9 | | 24 | | 6.56 | | 0.745 | |  |  |
| L | Left Anterior Cingulate Cortex (-) | | | 3 | -4 | | 6 | | 26 | | 6.56 | | 0.257 | |  |  |
| R | Right Lingual Gyrus (-) | | | 2 | 27 | | -98 | | -14 | | 5.82 | | 0.225 | |  |  |
| L | Left Middle Temporal Gyrus (-) | | | 2 | -48 | | -37 | | -6 | | 6.02 | | 0.428 | |  |  |
| L | Left Anterior Cingulate Cortex (-) | | | 2 | -4 | | 11 | | 24 | | 6.12 | | 1.032 | |  |  |
| R | Right Anterior Cingulate Cortex (-) | | | 2 | 8 | | 15 | | 24 | | 6.37 | | 0.778 | |  |  |
| R | Right Anterior Cingulate Cortex/Right Middle Cingulate Cortex (-) | | | 2 | 10 | | 15 | | 28 | | 5.56 | | 0.479 | |  |  |
| R | Right Middle Cingulate Cortex (-) | | | 2 | 18 | | -31 | | 46 | | 5.34 | | 0.254 | |  |  |

*X, Y, and Z values are cluster center of mass coordinates in MNI stereotactic space; Voxel stats column depicts the mean and standard deviation of the voxelwise statistics for each effect.*

**Supplemental Table 7. PTP vs. TEHC Abnormalities for Juice vs. Visual Cue Negative Temporal Prediction Errors (PEs)**

| **Hem.** | | **Region(s)** | **Voxels** | | **X** | **Y** | **Z** | | **Voxel Stats** | | | | | **Extracted Cluster Values** | | | | | | |  |  |
| --- | --- | --- | --- | --- | --- | --- | --- | --- | --- | --- | --- | --- | --- | --- | --- | --- | --- | --- | --- | --- | --- | --- |
|  |  |  |  |  |  |  |  |  | ***pTFCE Z*** | | | | | ***Juice Positive Temporal PE*** | | | ***Visual Cue Positive Temporal PE*** | | | |  |  |
|  |  |  |  |  |  |  |  |  | ***Mean*** | | ***SD*** | | | ***PTP*** | ***TEHC*** | | ***PTP*** | | ***TEHC*** | |  |  |
| **ROI Constrained Analysis** | | | | | | | | | | | | | | | | | | | | | | |
| L | Left Amygdala | | | 32 | -23 | -8 | | -12 | | 4.35 | | 0.135 | 0.000 | | | -0.138 | | -0.023 | | 0.035 | |  |
| L | Left Putamen/Left Pallidum | | | 8 | -28 | -11 | | -2 | | 4.42 | | 0.157 | 0.023 | | | -0.116 | | 0.006 | | -0.035 | |  |

*X, Y, and Z values are cluster center of mass coordinates in MNI stereotactic space; Voxel stats column depicts the mean and standard deviation of the voxelwise statistics for each effect; Extracted Cluster Values columns list the mean within-subject average % signal changes within identified effect clusters for each group for each of the two constituent conditions composing the juice vs. visual negative temporal PE contrast effect: unexpected vs. expected absence of juice (juice negative temporal PE) and unexpected vs. expected absence of visual cue (visual cue negative temporal PE); PTP = post-trauma psychopathology; TEHC = trauma-exposed healthy comparison group.*

**Supplemental Table 8. Regions Demonstrating Blood Oxygenation Level Dependent (BOLD) Encoding of Temporal Difference (TD) Learning Computational Model Prediction Errors (PEs) to Juice Predictive Cues**

| **Hem.** | | **Region(s)** | **Voxels** | | | **X** | | **Y** | | **Z** | | **Voxel Stats** | | | |  |
| --- | --- | --- | --- | --- | --- | --- | --- | --- | --- | --- | --- | --- | --- | --- | --- | --- |
|  |  |  |  |  |  |  |  |  |  |  |  | ***pTFCE Z*** | | | |  |
|  |  |  |  |  |  |  |  |  |  |  |  | ***Mean*** | | ***SD*** | |  |
| **ROI Constrained Analysis** | | | | | | | | | | | | | | | | |
| R | Right Insula Lobe/Right Rolandic Operculum/Right Putamen/Right Heschls Gyrus | | | 197 | 41 | | -5 | | 13 | | 6.15 | | 1.065 | |  |  |
| R | Right Caudate Nucleus/Right Olfactory cortex/Right Putamen | | | 127 | 11 | | 20 | | 3 | | 6.13 | | 0.974 | |  |  |
| R | Right Caudate Nucleus | | | 109 | 13 | | -7 | | 21 | | 5.29 | | 0.637 | |  |  |
| L | Left Caudate Nucleus | | | 69 | -11 | | 21 | | 5 | | 6.04 | | 0.999 | |  |  |
| L | Left Caudate Nucleus | | | 61 | -20 | | -29 | | 15 | | 5.84 | | 0.802 | |  |  |
| R | Right Insula/Right Superior Temporal Gyrus | | | 47 | 43 | | -41 | | 24 | | 5.24 | | 0.511 | |  |  |
| L | Left Insula Lobe/Left Rolandic Operculum | | | 41 | -37 | | -10 | | 23 | | 5.43 | | 0.873 | |  |  |
| L/R | Left Anterior Cingulate Cortex/Right Mid Orbital Gyrus/Left Mid Orbital Gyrus/Left Superior Medial Gyrus (-) | | | 40 | -1 | | 50 | | 2 | | -4.13 | | 0.118 | |  |  |
| R | Right Caudate Nucleus | | | 20 | 18 | | -25 | | 20 | | 5.22 | | 0.646 | |  |  |
| L | Left Insula/Left Rolandic Operculum | | | 20 | -46 | | -20 | | 25 | | 4.96 | | 0.644 | |  |  |
| L | Left Insula Lobe | | | 15 | -35 | | -12 | | 13 | | 3.92 | | 0.015 | |  |  |
| R | Right Caudate Nucleus/Hippocampus | | | 11 | 27 | | -35 | | 8 | | 5.39 | | 0.857 | |  |  |
| R | Right Insula Lobe | | | 11 | 35 | | 11 | | 14 | | 4.55 | | 0.323 | |  |  |
| L/R | Right Olfactory cortex/Left Caudate Nucleus | | | 8 | 1 | | 15 | | 0 | | 4.60 | | 0.212 | |  |  |
| L | Left Caudate Nucleus | | | 6 | -14 | | 2 | | 22 | | 4.80 | | 0.264 | |  |  |
| L | Left Rolandic Operculum | | | 4 | -46 | | -8 | | 14 | | 4.31 | | 0.156 | |  |  |
| R | Right Caudate Nucleus | | | 4 | 15 | | 9 | | 20 | | 5.03 | | 0.778 | |  |  |
| L | Left Hippocampus | | | 3 | -28 | | -37 | | 3 | | 6.01 | | 0.665 | |  |  |
| L | Left Caudate Nucleus | | | 3 | -6 | | -1 | | 11 | | 4.60 | | 0.223 | |  |  |
| R | Right Caudate Nucleus | | | 3 | 17 | | 18 | | 14 | | 5.60 | | 0.629 | |  |  |
| L | Left Caudate Nucleus | | | 3 | -15 | | 15 | | 16 | | 5.24 | | 0.721 | |  |  |
| R | Right Caudate Nucleus | | | 3 | 17 | | 13 | | 18 | | 5.18 | | 0.588 | |  |  |
| L | Left Caudate Nucleus | | | 3 | -15 | | 9 | | 20 | | 4.99 | | 0.685 | |  |  |
| L/R | Left Anterior Cingulate Cortex/Left Mid Orbital Gyrus/Right Mid Orbital Gyrus (-) | | | 3 | -1 | | 37 | | -8 | | -4.05 | | 0.032 | |  |  |
| **Whole Brain Analysis** | | | | | | | | | | | | | | | | |
| R | Right Middle Frontal Gyrus/Right Precentral Gyrus/Right Postcentral Gyrus/Right Superior Frontal Gyrus/Right Rolandic Operculum/Right SupraMarginal Gyrus/Right Angular Gyrus/Right Inferior Frontal Gyrus (p. Opercularis)/Right Middle Temporal Gyrus/Right Insula Lobe/Right Inferior Frontal Gyrus (p. Triangularis)/Right Superior Occipital Gyrus/Right SMA/Right Superior Temporal Gyrus/Right Middle Occipital Gyrus/Right Precuneus/Right Inferior Parietal Lobule/Right Cuneus/Right Superior Parietal Lobule/Right Heschls Gyrus/Right Putamen/Right Temporal Pole/Right Inferior Temporal Gyrus | | | 6861 | 43 | | -5 | | 34 | | 6.88 | | 1.304 | |  |  |
| L | Left Postcentral Gyrus/Left Middle Frontal Gyrus/Left Precentral Gyrus/Left Superior Frontal Gyrus/Left Rolandic Operculum/Left Inferior Parietal Lobule/Left SupraMarginal Gyrus/Left SMA/Left Inferior Frontal Gyrus (p. Triangularis)/Left Middle Cingulate Cortex/Left Superior Temporal Gyrus/Left Inferior Frontal Gyrus (p. Opercularis)/Left Superior Medial Gyrus/Left Heschls Gyrus/Left Anterior Cingulate Cortex/Left Paracentral Lobule/Left Superior Orbital Gyrus | | | 4688 | -40 | | 3 | | 35 | | 7.10 | | 1.265 | |  |  |
| L/R | Left Cerebellum (VI)/Right Cerebellum (VI)/Left Cerebellum (Crus 1)/Right Cerebellum (Crus 1)/Right Lingual Gyrus/Right Fusiform Gyrus/Left Cerebellum (Crus 2)/Cerebellar Vermis (6)/Left Lingual Gyrus/Left Fusiform Gyrus/Cerebellar Vermis (7)/Left Calcarine Gyrus/Left Cerebellum (IV-V)/Right Cerebellum (Crus 2)/Right Calcarine Gyrus/Right Inferior Occipital Gyrus | | | 2813 | 3 | | -78 | | -19 | | 6.12 | | 0.737 | |  |  |
| L/R | Right Caudate Nucleus/Left Caudate Nucleus/Right Thalamus/Left Thalamus/Left Hippocampus/Right Hippocampus/Cerebellar Vermis (3)/Right Olfactory cortex/Right Rectal Gyrus/Right Lingual Gyrus/Right Posterior Cingulate Cortex/Right Putamen/Left Posterior Cingulate Cortex | | | 1224 | 3 | | -4 | | 11 | | 6.58 | | 1.122 | |  |  |
| L/R | Right Superior Occipital Gyrus/Right Middle Occipital Gyrus/Right Cuneus/Left Cuneus | | | 494 | 22 | | -88 | | 25 | | 5.12 | | 0.238 | |  |  |
| R | Right Hippocampus/Right Calcarine Gyrus/Right Fusiform Gyrus/Right ParaHippocampal Gyrus/Right Lingual Gyrus/Right Amygdala/Right Precuneus/Right Inferior Temporal Gyrus | | | 439 | 35 | | -33 | | -9 | | 6.50 | | 1.062 | |  |  |
| L | Left Superior Parietal Lobule/Left Angular Gyrus/Left Precuneus/Left Middle Occipital Gyrus/Left Inferior Parietal Lobule/Left Postcentral Gyrus/Left Superior Occipital Gyrus | | | 395 | -23 | | -57 | | 47 | | 6.23 | | 0.931 | |  |  |
| L | Left Superior Occipital Gyrus/Left Middle Occipital Gyrus/Left Cuneus/Left Calcarine Gyrus | | | 278 | -17 | | -95 | | 14 | | 5.68 | | 0.619 | |  |  |
| L/R | Right SMA/Left Paracentral Lobule/Left Middle Cingulate Cortex/Left SMA/Right Middle Cingulate Cortex | | | 238 | 3 | | -20 | | 56 | | -4.78 | | 0.150 | |  |  |
| L | Left Hippocampus/Left Calcarine Gyrus/Left Precuneus/Left Lingual Gyrus/Left ParaHippocampal Gyrus/Left Inferior Temporal Gyrus/Left Fusiform Gyrus | | | 233 | -30 | | -45 | | -1 | | 7.18 | | 1.194 | |  |  |
| L | Left Middle Occipital Gyrus/Left Inferior Occipital Gyrus/Left Middle Temporal Gyrus/Left Fusiform Gyrus/Left Angular Gyrus/Left Lingual Gyrus | | | 204 | -34 | | -76 | | 5 | | 5.88 | | 0.946 | |  |  |
| L/R | Left Rectal Gyrus/Left Mid Orbital Gyrus/Right Mid Orbital Gyrus/Right Rectal Gyrus/Left Anterior Cingulate Cortex | | | 156 | -1 | | 37 | | -14 | | -4.78 | | 0.120 | |  |  |
| R | Right Middle Orbital Gyrus/Right Inferior Frontal Gyrus (p. Orbitalis)/Right Superior Orbital Gyrus | | | 132 | 28 | | 40 | | -11 | | 5.96 | | 0.640 | |  |  |
| L/R | Left Superior Medial Gyrus/Left Anterior Cingulate Cortex/Left Mid Orbital Gyrus/Right Superior Medial Gyrus/Right Mid Orbital Gyrus | | | 76 | -1 | | 57 | | 1 | | -4.69 | | 0.085 | |  |  |
| R | Right Middle Occipital Gyrus/Right Inferior Occipital Gyrus/Right Fusiform Gyrus | | | 58 | 33 | | -81 | | 4 | | 6.10 | | 0.974 | |  |  |
| R | Right Middle Occipital Gyrus/Right Middle Temporal Gyrus | | | 54 | 39 | | -69 | | 12 | | 5.95 | | 0.952 | |  |  |
| R | Right Inferior Occipital Gyrus/Right Inferior Temporal Gyrus/Right Middle Occipital Gyrus/Right Middle Temporal Gyrus | | | 49 | 38 | | -66 | | -3 | | 5.83 | | 0.850 | |  |  |
| L/R | Cerebellar Vermis (4/5)/Right Cerebellum (IV-V)/Cerebellar Vermis (3) | | | 45 | 5 | | -46 | | -3 | | 5.21 | | 0.305 | |  |  |
| L | Left Middle Temporal Gyrus/Left Middle Occipital Gyrus | | | 37 | -45 | | -55 | | 4 | | 5.63 | | 0.652 | |  |  |
| L | Left Inferior Parietal Lobule/Left Postcentral Gyrus/Left Superior Parietal Lobule | | | 28 | -27 | | -43 | | 47 | | 5.95 | | 0.815 | |  |  |
| L/R | Left Superior Medial Gyrus/Right Superior Medial Gyrus | | | 27 | -1 | | 34 | | 48 | | -4.72 | | 0.104 | |  |  |
| L | Left Superior Medial Gyrus/Left Anterior Cingulate Cortex | | | 15 | -14 | | 41 | | 18 | | 5.78 | | 0.446 | |  |  |
| R | Right Superior Temporal Gyrus/Right Middle Temporal Gyrus | | | 12 | 46 | | -33 | | 4 | | 5.11 | | 0.281 | |  |  |
| L | Left Middle Cingulate Cortex | | | 11 | -16 | | -23 | | 41 | | 6.26 | | 0.869 | |  |  |
| L | Left Middle Occipital Gyrus | | | 8 | -31 | | -75 | | 17 | | 4.98 | | 0.188 | |  |  |
| L | Left Cuneus | | | 8 | -3 | | -91 | | 33 | | 4.70 | | 0.006 | |  |  |
| L | Left Superior Medial Gyrus/Left Mid Orbital Gyrus/Left Superior Orbital Gyrus | | | 7 | -13 | | 55 | | 0 | | 4.69 | | 0.004 | |  |  |
| R | Right Insula Lobe | | | 7 | 35 | | 11 | | 13 | | 4.85 | | 0.173 | |  |  |
| R | Right Calcarine Gyrus/Right Superior Occipital Gyrus/Right Middle Occipital Gyrus | | | 6 | 23 | | -91 | | 3 | | 5.11 | | 0.281 | |  |  |
| L | Left Rolandic Operculum/Left Insula Lobe | | | 6 | -38 | | -9 | | 20 | | 5.16 | | 0.271 | |  |  |
| L | Left Calcarine Gyrus/Left Lingual Gyrus | | | 5 | -24 | | -72 | | 3 | | 5.38 | | 0.540 | |  |  |
| L | Left Middle Occipital Gyrus | | | 3 | -29 | | -84 | | 3 | | 4.79 | | 0.100 | |  |  |
| R | Right Calcarine Gyrus | | | 3 | 20 | | -79 | | 11 | | 4.79 | | 0.100 | |  |  |
| L | Left Cuneus | | | 3 | -1 | | -94 | | 25 | | 4.79 | | 0.098 | |  |  |
| R | Right Middle Cingulate Cortex | | | 3 | 14 | | 3 | | 41 | | 4.96 | | 0.277 | |  |  |
| L | Left Middle Temporal Gyrus/Left Inferior Temporal Gyrus | | | 2 | -48 | | -37 | | -14 | | 4.84 | | 0.149 | |  |  |
| R | Right Fusiform Gyrus | | | 2 | 32 | | -59 | | -4 | | 5.64 | | 0.120 | |  |  |
| R | Right Middle Occipital Gyrus/Right Calcarine Gyrus | | | 2 | 26 | | -88 | | 3 | | 5.12 | | 0.138 | |  |  |
| L | Left Thalamus | | | 2 | -2 | | -28 | | 7 | | 5.10 | | 0.416 | |  |  |
| L | Left Caudate Nucleus | | | 2 | -8 | | -3 | | 18 | | 5.26 | | 0.000 | |  |  |
| L | Left Insula Lobe/Left Rolandic Operculum | | | 2 | -39 | | -12 | | 22 | | 5.75 | | 0.233 | |  |  |
| L | Left SupraMarginal Gyrus | | | 2 | -47 | | -26 | | 26 | | 4.84 | | 0.149 | |  |  |
| L | Left Middle Cingulate Cortex | | | 2 | -14 | | -10 | | 43 | | 5.99 | | 0.000 | |  |  |

*X, Y, and Z values are cluster center of mass coordinates in MNI stereotactic space; Voxel stats column depicts the mean and standard deviation of the voxelwise statistics for each effect.*

**Supplemental Table 9. PTP vs. TEHC Abnormalities for Blood Oxygenation Level Dependent (BOLD) Encoding of Temporal Difference (TD) Learning Computational Model Prediction Errors (PEs) to Juice Predictive Cues**

| **Hem.** | | **Region(s)** | **Voxels** | | **X** | **Y** | **Z** | | **Voxel Stats** | | | | | **Extracted Cluster Values** | | |  |  |
| --- | --- | --- | --- | --- | --- | --- | --- | --- | --- | --- | --- | --- | --- | --- | --- | --- | --- | --- |
|  |  |  |  |  |  |  |  |  | ***pTFCE Z*** | | | | | ***Juice Cue TD Learning PE*** | | |  |  |
|  |  |  |  |  |  |  |  |  | ***Mean*** | | ***SD*** | | | ***PTP*** | ***TEHC*** | |  |  |
| **ROI Constrained Analysis** | | | | | | | | | | | | | | | | | | |
| R | Right Insula Lobe/Right Rolandic Operculum/Right Putamen/Right Inferior Frontal Gyrus (p. Opercularis)/Right Heschls Gyrus/Right Inferior Frontal Gyrus (p. Triangularis) | | | 670 | 41 | 4 | | 3 | | 4.38 | | 0.320 | 0.168 | | | -0.156 | |  |
| R | Right Putamen/Right Caudate Nucleus/Right Pallidum/Right Rectal Gyrus | | | 184 | 21 | 14 | | 1 | | 4.23 | | 0.169 | 0.118 | | | -0.235 | |  |
| L | Left Insula Lobe/Left Inferior Frontal Gyrus (p. Opercularis)/Left Putamen/Left Rolandic Operculum | | | 167 | -38 | 6 | | 2 | | 4.12 | | 0.152 | 0.146 | | | -0.199 | |  |
| L | Left Putamen/Left Pallidum | | | 32 | -28 | -3 | | 5 | | 3.99 | | 0.053 | 0.085 | | | -0.343 | |  |
| **Whole Brain Analysis** | | | | | | | | | | | | | | | | | |  |
| R | Right Insula Lobe/Right Inferior Frontal Gyrus (p. Orbitalis)/Right Inferior Frontal Gyrus (p. Opercularis)/Right Putamen/Right Inferior Frontal Gyrus (p. Triangularis)/Right Rolandic Operculum/Right Caudate Nucleus/Right Temporal Pole/Right Middle Frontal Gyrus/Right Heschls Gyrus/Right Pallidum/Right Superior Temporal Gyrus/Right Middle Orbital Gyrus | | | 1580 | 46 | 16 | | 1 | | 5.00 | | 0.332 | 0.181 | | | -0.205 | |  |
| R | Right SupraMarginal Gyrus/Right Superior Temporal Gyrus/Right Rolandic Operculum/Right Postcentral Gyrus/Right Angular Gyrus/Right Inferior Parietal Lobule | | | 268 | 64 | -36 | | 27 | | 4.70 | | 0.107 | 0.178 | | | -0.189 | |  |
| L | Left Insula Lobe/Left Putamen/Left Inferior Frontal Gyrus (p. Opercularis) | | | 50 | -37 | 5 | | 0 | | 4.67 | | 0.084 | 0.145 | | | -0.249 | |  |

*X, Y, and Z values are cluster center of mass coordinates in MNI stereotactic space; Voxel stats column depicts the mean and standard deviation of the voxelwise statistics for each effect; Extracted Cluster Values columns list the mean within-subject average % signal changes within identified effect clusters for each group for the parametric modulation of BOLD signal as a function of TD learning PEs to juice-predictive cues; PTP = post-trauma psychopathology; TEHC = trauma-exposed healthy comparison group.*

**Supplemental Table 10. Regions Demonstrating Blood Oxygenation-Level Dependent (BOLD) Encoding of Temporal Difference (TD) Learning Computational Model Prediction Errors (PEs) to Juice Receipt**

| **Hem.** | | **Region(s)** | **Voxels** | | | **X** | | **Y** | | **Z** | | **Voxel Stats** | | | |  |
| --- | --- | --- | --- | --- | --- | --- | --- | --- | --- | --- | --- | --- | --- | --- | --- | --- |
|  |  |  |  |  |  |  |  |  |  |  |  | ***pTFCE Z*** | | | |  |
|  |  |  |  |  |  |  |  |  |  |  |  | ***Mean*** | | ***SD*** | |  |
| **ROI Constrained Analysis** | | | | | | | | | | | | | | | | |
| L/R | Left Insula Lobe/Right Insula Lobe/Left Putamen/Right Putamen/Left Rolandic Operculum/Right Caudate Nucleus/Right Pallidum/Left Pallidum/Left Caudate Nucleus/Right Rolandic Operculum/Left Amygdala/Right Amygdala/Right ParaHippocampal Gyrus/Right Inferior Frontal Gyrus (p. Opercularis)/Left Inferior Frontal Gyrus (p. Opercularis)/Left Inferior Frontal Gyrus (p. Triangularis)/Left ParaHippocampal Gyrus/Left Heschls Gyrus/Right Inferior Frontal Gyrus (p. Triangularis)/Right Heschls Gyrus/Left Temporal Pole/Left Olfactory cortex/Right Olfactory cortex/Right Rectal Gyrus/Left Rectal Gyrus/Left Inferior Frontal Gyrus (p. Orbitalis) | | | 10608 | 0 | | -3 | | 2 | | 10.42 | | 2.642 | |  |  |
| R | Right Insula Lobe/Right Rolandic Operculum/Right Heschls Gyrus | | | 208 | 51 | | -31 | | 23 | | 9.02 | | 2.313 | |  |  |
| R | Right Caudate Nucleus | | | 49 | 27 | | -30 | | 3 | | 6.64 | | 1.437 | |  |  |
| L | Left Caudate Nucleus | | | 31 | -23 | | -34 | | 7 | | 6.78 | | 0.925 | |  |  |
| R | Right Insula Lobe/Right Heschls Gyrus | | | 27 | 30 | | -24 | | 16 | | 6.79 | | 1.536 | |  |  |
| L | Left Anterior Cingulate Cortex/Left Superior Medial Gyrus/Left Mid Orbital Gyrus | | | 23 | -13 | | 48 | | -1 | | 5.98 | | 0.804 | |  |  |
| R | Right Caudate Nucleus | | | 8 | 33 | | -21 | | -9 | | 9.34 | | 1.353 | |  |  |
| **Whole Brain Analysis** | | | | | | | | | | | | | | | | |
| L/R | Right Middle Temporal Gyrus/Left Middle Temporal Gyrus/Right Precuneus/Left Precuneus/Right Superior Temporal Gyrus/Left Postcentral Gyrus/Left Middle Occipital Gyrus/Right Postcentral Gyrus/Right Middle Frontal Gyrus/Right Inferior Temporal Gyrus/Left Superior Temporal Gyrus/Left Inferior Parietal Lobule/Left Inferior Temporal Gyrus/Right Middle Cingulate Cortex/Right SupraMarginal Gyrus/Right Fusiform Gyrus/Right Middle Occipital Gyrus/Left Cerebellum (Crus 1)/Left Superior Parietal Lobule/Left Middle Cingulate Cortex/Left Middle Frontal Gyrus/Right Lingual Gyrus/Right Superior Parietal Lobule/Right Precentral Gyrus/Left Lingual Gyrus/Left Cerebellum (VI)/Left Fusiform Gyrus/Right Insula Lobe/Left Insula Lobe/Right Angular Gyrus/Right Cerebellum (VI)/Left Calcarine Gyrus/Left SMA/Left Cuneus/Right Superior Frontal Gyrus/Right SMA/Left Precentral Gyrus/Right Inferior Frontal Gyrus (p. Triangularis)/Right Calcarine Gyrus/Right Inferior Parietal Lobule/Left Inferior Frontal Gyrus (p. Triangularis)/Right Cuneus/Left SupraMarginal Gyrus/Right Inferior Frontal Gyrus (p. Opercularis)/Right Rolandic Operculum/Right Putamen/Right Cerebellum (Crus 1)/Right Superior Occipital Gyrus/Left Superior Occipital Gyrus/Right Thalamus/Left Thalamus/Left Putamen/Left Rolandic Operculum/Left Inferior Frontal Gyrus (p. Opercularis)/Left Superior Frontal Gyrus/Left Cerebellum (Crus 2)/Right ParaHippocampal Gyrus/Right Cerebellum (Crus 2)/Right Inferior Occipital Gyrus/Left Cerebellum (VIII)/Left Inferior Occipital Gyrus/Left Angular Gyrus/Right Temporal Pole/Left Temporal Pole/Left ParaHippocampal Gyrus/Left Anterior Cingulate Cortex/Right Hippocampus/Right Caudate Nucleus/Left Hippocampus/Right Cerebellum (VIII)/Left Posterior Cingulate Cortex/Left Superior Medial Gyrus/Right Medial Temporal Pole/Left Cerebellum (IV-V)/Cerebellar Vermis (6)/Right Inferior Frontal Gyrus (p. Orbitalis)/Left Caudate Nucleus/Left Cerebellum (VII)/Right Pallidum/Right Superior Medial Gyrus/Right Paracentral Lobule/Right Amygdala/Left Medial Temporal Pole/Right Anterior Cingulate Cortex/Right Posterior Cingulate Cortex/Cerebellar Vermis (4/5)/Left Pallidum/Right Cerebellum (IV-V)/Left Amygdala/Right Cerebellum (VII)/Left Paracentral Lobule/Left Heschls Gyrus/Left Inferior Frontal Gyrus (p. Orbitalis)/Right Superior Orbital Gyrus/Left Cerebellum (IX)/Right Heschls Gyrus/Right Middle Orbital Gyrus/Right Olfactory cortex/Cerebellar Vermis (3)/Left Cerebellum (X)/Right Cerebellum (III)/Cerebellar Vermis (7)/Right Cerebellum (X)/Left Olfactory cortex/Left Cerebellum (III)/Cerebellar Vermis (1/2)/Cerebellar Vermis (8)/Cerebellar Vermis (10)/Left Middle Orbital Gyrus/Right Cerebellum (IX)/Left Superior Orbital Gyrus/Right Rectal Gyrus/Cerebellar Vermis (9)/Right Mid Orbital Gyrus/Left Rectal Gyrus | | | 112709 | 3 | | -32 | | 13 | | 9.06 | | 2.647 | |  |  |
| L | Left Superior Medial Gyrus/Left Anterior Cingulate Cortex/Left Mid Orbital Gyrus/Left Superior Frontal Gyrus | | | 126 | -13 | | 48 | | 8 | | 5.62 | | 0.881 | |  |  |
| L | Left Superior Frontal Gyrus/Left Middle Frontal Gyrus | | | 8 | -17 | | 19 | | 47 | | 4.98 | | 0.456 | |  |  |
| L | Left Cerebellum (Crus 2)/Left Cerebellum (Crus 1) | | | 3 | -50 | | -70 | | -40 | | 4.97 | | 0.313 | |  |  |
| L | Left Cerebellum (VII)/Left Cerebellum (Crus 1) | | | 2 | -38 | | -38 | | -39 | | 4.65 | | 0.001 | |  |  |
| L | Left Middle Temporal Gyrus/Left Inferior Temporal Gyrus | | | 2 | -68 | | -48 | | -11 | | 4.65 | | 0.001 | |  |  |
| L | Left Middle Temporal Gyrus | | | 2 | -68 | | -50 | | -7 | | 5.12 | | 0.470 | |  |  |
| R | Right Superior Frontal Gyrus | | | 2 | 20 | | 13 | | 46 | | 5.12 | | 0.470 | |  |  |

*X, Y, and Z values are cluster center of mass coordinates in MNI stereotactic space; Voxel stats column depicts the mean and standard deviation of the voxelwise statistics for each effect.*

**Supplemental Table 11. PTP vs. TEHC Abnormalities for Blood Oxygenation Level Dependent (BOLD) Encoding of Temporal Difference (TD) Learning Computational Model Prediction Errors (PEs) to Juice Delivery**

| **Hem.** | | **Region(s)** | **Voxels** | | **X** | **Y** | **Z** | | **Voxel Stats** | | | | | **Extracted Cluster Values** | | |  |  |
| --- | --- | --- | --- | --- | --- | --- | --- | --- | --- | --- | --- | --- | --- | --- | --- | --- | --- | --- |
|  |  |  |  |  |  |  |  |  | ***pTFCE Z*** | | | | | ***Juice Cue TD Learning PE*** | | |  |  |
|  |  |  |  |  |  |  |  |  | ***Mean*** | | ***SD*** | | | ***PTP*** | ***TEHC*** | |  |  |
| **ROI Constrained Analysis** | | | | | | | | | | | | | | | | | | |
| L | Left Amygdala (-) | | | 22 | -29 | -7 | | -13 | | 3.92 | | 0.026 | 0.054 | | | 0.174 | |  |
| L | Left Putamen (-) | | | 18 | -31 | -11 | | 1 | | 4.09 | | 0.063 | 0.035 | | | 0.156 | |  |

*X, Y, and Z values are cluster center of mass coordinates in MNI stereotactic space; Voxel stats column depicts the mean and standard deviation of the voxelwise statistics for each effect; Extracted Cluster Values columns list the mean within-subject average % signal changes within identified effect clusters for each group for the parametric modulation of BOLD signal as a function of TD learning PEs to juice delivery; (-) sign indicates decreased activation for the post-trauma psychopathology (PTP) vs. trauma-exposed healthy comparison (TEHC) groups.*

**Supplemental Table 12. Regions Demonstrating Blood Oxygenation-Level Dependent (BOLD) Encoding of Temporal Difference (TD) Learning Computational Model Prediction Errors (PEs) to Non-Juice Predictive Cues**

| **Hem.** | | **Region(s)** | **Voxels** | | | | | **X** | | | **Y** | | **Z** | | | **Voxel Stats** | | | | | |  |
| --- | --- | --- | --- | --- | --- | --- | --- | --- | --- | --- | --- | --- | --- | --- | --- | --- | --- | --- | --- | --- | --- | --- |
|  |  |  |  |  |  |  |  |  |  |  |  |  |  |  |  | ***pTFCE Z*** | | | | | |  |
|  |  |  |  |  |  |  |  |  |  |  |  |  |  |  |  | ***Mean*** | | | ***SD*** | | |  |
| **ROI Constrained Analysis** | | | | | | | | | | | | | | | | | | | | | | |
| R | Right Mid Orbital Gyrus/Right Anterior Cingulate Cortex (-) | | | 24 | | 8 | | | 41 | | | -4 | | | -4.09 | | | 0.116 | | |  |  |
| L | Left Rolandic Operculum/Left Insula Lobe | | | 21 | | -45 | | | -11 | | | 20 | | | 4.38 | | | 0.235 | | |  |  |
| R | Right Insula Lobe/Right Superior Temporal Gyrus | | | 16 | | 41 | | | -40 | | | 24 | | | 3.99 | | | 0.026 | | |  |  |
| L | Left Anterior Cingulate Cortex/Left Superior Medial Gyrus (-) | | | 16 | | -3 | | | 46 | | | 2 | | | -4.00 | | | 0.035 | | |  |  |
| R | Right Caudate Nucleus/Right Pallidum | | | 4 | | 12 | | | 9 | | | 0 | | | 3.98 | | | 0.029 | | |  |  |
| **Whole Brain Analysis** | | | | | | | | | | | | | | | | | | | | | | |
| R | Right Postcentral Gyrus/Right Precentral Gyrus/Right SupraMarginal Gyrus/Right Middle Frontal Gyrus/Right Rolandic Operculum/Right Inferior Frontal Gyrus (p. Opercularis) | | | | 1045 | | 55 | | | -7 | | | | 34 | | | 4.85 | | | 0.160 | | |
| L | Left Precentral Gyrus/Left Postcentral Gyrus/Left Rolandic Operculum/Left Middle Frontal Gyrus/Left SMA/Left Superior Frontal Gyrus/Left Paracentral Lobule | | | | 902 | | -40 | | | -10 | | | | 42 | | | 4.93 | | | 0.243 | | |
| R | Right Middle Temporal Gyrus/Right Middle Occipital Gyrus/Right Inferior Temporal Gyrus/Right Inferior Occipital Gyrus | | | | 54 | | 45 | | | -71 | | | | 1 | | | 4.68 | | | 0.085 | | |
| R | Right Postcentral Gyrus/Right Superior Parietal Lobule/Right Precuneus | | | | 25 | | 17 | | | -42 | | | | 66 | | | 4.70 | | | 0.083 | | |
| L | Left Precuneus/Left Paracentral Lobule/Left Postcentral Gyrus/Left Superior Parietal Lobule | | | | 21 | | -15 | | | -39 | | | | 66 | | | 4.68 | | | 0.063 | | |
| R | Right Superior Parietal Lobule/Right Inferior Parietal Lobule/Right Postcentral Gyrus | | | | 10 | | 34 | | | -45 | | | | 57 | | | 4.66 | | | 0.041 | | |
| L | Left Inferior Parietal Lobule/Left Postcentral Gyrus | | | | 5 | | -32 | | | -36 | | | | 42 | | | 4.64 | | | 0.034 | | |
| R | Right Superior Frontal Gyrus/Right SMA | | | | 4 | | 20 | | | 6 | | | | 62 | | | 4.59 | | | 0.018 | | |
| R | Right Mid Orbital Gyrus/Right Anterior Cingulate Cortex | | | | 2 | | 9 | | | 40 | | | | -6 | | | -4.61 | | | 0.007 | | |

*X, Y, and Z values are cluster center of mass coordinates in MNI stereotactic space; Voxel stats column depicts the mean and standard deviation of the voxelwise statistics for each effect.*

**Supplemental Table 13. Regions Demonstrating Blood Oxygenation-Level Dependent (BOLD) Encoding of Temporal Difference (TD) Learning Computational Model Prediction Errors (PEs) to Visual Cue Receipt**

| **Hem.** | | **Region(s)** | **Voxels** | | | **X** | | **Y** | | **Z** | | **Voxel Stats** | | | |  |
| --- | --- | --- | --- | --- | --- | --- | --- | --- | --- | --- | --- | --- | --- | --- | --- | --- |
|  |  |  |  |  |  |  |  |  |  |  |  | ***pTFCE Z*** | | | |  |
|  |  |  |  |  |  |  |  |  |  |  |  | ***Mean*** | | ***SD*** | |  |
| **ROI Constrained Analysis** | | | | | | | | | | | | | | | | |
| L/R | Left Putamen/Left Caudate Nucleus/Left Insula Lobe/Left Pallidum/Left Rolandic Operculum/Left Heschls Gyrus/Left Olfactory cortex/Left Amygdala/Left Rectal Gyrus | | | 2154 | -22 | | -7 | | 4 | | 6.60 | | 1.564 | |  |  |
| R | Right Caudate Nucleus/Right Putamen/Right Pallidum/Right Amygdala | | | 1207 | 20 | | 1 | | 6 | | 6.80 | | 1.595 | |  |  |
| R | Right Insula Lobe/Right Putamen/Right Inferior Frontal Gyrus (p. Opercularis)/Right Inferior Frontal Gyrus (p. Triangularis)/Right Rolandic Operculum | | | 625 | 37 | | 13 | | 4 | | 7.59 | | 2.143 | |  |  |
| L | Left Insula Lobe/Left Inferior Frontal Gyrus (p. Triangularis)/Left Inferior Frontal Gyrus (p. Opercularis)/Left Temporal Pole/Left Inferior Frontal Gyrus (p. Orbitalis)/Left Superior Temporal Gyrus/Left Rolandic Operculum | | | 547 | -37 | | 16 | | 3 | | 7.70 | | 1.465 | |  |  |
| R | Right Caudate Nucleus | | | 60 | 28 | | -31 | | 0 | | 8.86 | | 2.438 | |  |  |
| R | Right Superior Temporal Gyrus/Right Heschls Gyrus/Right Insula Lobe | | | 57 | 46 | | -21 | | -3 | | 8.85 | | 2.770 | |  |  |
| L | Left Amygdala/Left ParaHippocampal Gyrus | | | 55 | -29 | | -5 | | -21 | | 5.87 | | 1.119 | |  |  |
| R | Right Amygdala | | | 32 | 28 | | -1 | | -16 | | 6.05 | | 1.171 | |  |  |
| R | Right Heschls Gyrus/Right Insula Lobe/Right Superior Temporal Gyrus | | | 31 | 37 | | -25 | | 12 | | 5.43 | | 0.513 | |  |  |
| L | Left Caudate Nucleus | | | 23 | -25 | | -32 | | 1 | | 6.73 | | 1.607 | |  |  |
| L | Left Insula Lobe/Left Superior Temporal Gyrus/Left Rolandic Operculum | | | 16 | -43 | | -38 | | 24 | | 4.73 | | 0.006 | |  |  |
| L | Left Insula Lobe/Left Inferior Frontal Gyrus (p. Opercularis) | | | 15 | -31 | | 4 | | 19 | | 5.25 | | 0.454 | |  |  |
| R | Right Insula Lobe/Right Superior Temporal Gyrus | | | 13 | 49 | | -41 | | 23 | | 5.86 | | 0.938 | |  |  |
| L | Left Insula Lobe | | | 6 | -31 | | -8 | | 16 | | 5.46 | | 0.726 | |  |  |
| R | Right Heschls Gyrus/Right Insula Lobe | | | 4 | 49 | | -16 | | 7 | | 5.83 | | 0.879 | |  |  |
| L | Left Insula Lobe | | | 4 | -34 | | 1 | | 7 | | 5.01 | | 0.568 | |  |  |
| L | Left Insula Lobe | | | 2 | -32 | | -6 | | 13 | | 4.72 | | 0.000 | |  |  |
| **Whole Brain Analysis** | | | | | | | | | | | | | | | | |
| L/R | Right Middle Temporal Gyrus/Left Middle Temporal Gyrus/Left Middle Occipital Gyrus/Right Middle Frontal Gyrus/Right Lingual Gyrus/Right Precuneus/Right Superior Temporal Gyrus/Right Fusiform Gyrus/Left Fusiform Gyrus/Left Calcarine Gyrus/Left Lingual Gyrus/Left Inferior Temporal Gyrus/Right Middle Occipital Gyrus/Left Precentral Gyrus/Left Cerebellum (Crus 1)/Right Calcarine Gyrus/Right Inferior Temporal Gyrus/Left Superior Temporal Gyrus/Right Cerebellum (VI)/Left Precuneus/Left Cerebellum (VI)/Left Middle Frontal Gyrus/Left Inferior Parietal Lobule/Right Cuneus/Left Superior Parietal Lobule/Right Superior Occipital Gyrus/Right Cerebellum (Crus 1)/Left Cuneus/Left Superior Occipital Gyrus/Left Postcentral Gyrus/Right Superior Parietal Lobule/Right Middle Cingulate Cortex/Left SMA/Right Inferior Frontal Gyrus (p. Triangularis)/Right Inferior Parietal Lobule/Left Cerebellum (Crus 2)/Left Inferior Frontal Gyrus (p. Triangularis)/Right Precentral Gyrus/Left Insula Lobe/Right Superior Frontal Gyrus/Right Inferior Frontal Gyrus (p. Opercularis)/Left Inferior Occipital Gyrus/Right Thalamus/Left Thalamus/Right SMA/Left Cerebellum (VIII)/Right Angular Gyrus/Left Middle Cingulate Cortex/Right Insula Lobe/Right Inferior Occipital Gyrus/Left Putamen/Right SupraMarginal Gyrus/Right Cerebellum (IV-V)/Right Putamen/Left Superior Medial Gyrus/Left Cerebellum (IV-V)/Right Inferior Frontal Gyrus (p. Orbitalis)/Right ParaHippocampal Gyrus/Left Inferior Frontal Gyrus (p. Opercularis)/Left Superior Frontal Gyrus/Right Caudate Nucleus/Left Temporal Pole/Right Temporal Pole/Left Cerebellum (VII)/Left ParaHippocampal Gyrus/Left Caudate Nucleus/Right Superior Medial Gyrus/Left SupraMarginal Gyrus/Right Cerebellum (Crus 2)/Cerebellar Vermis (4/5)/Left Rolandic Operculum/Cerebellar Vermis (6)/Right Middle Orbital Gyrus/Right Cerebellum (VIII)/Right Hippocampus/Left Hippocampus/Left Anterior Cingulate Cortex/Left Angular Gyrus/Right Anterior Cingulate Cortex/Left Inferior Frontal Gyrus (p. Orbitalis)/Left Medial Temporal Pole/Right Superior Orbital Gyrus/Left Cerebellum (IX)/Cerebellar Vermis (7)/Left Paracentral Lobule/Right Posterior Cingulate Cortex/Cerebellar Vermis (8)/Left Posterior Cingulate Cortex/Left Heschls Gyrus/Right Pallidum/Right Postcentral Gyrus/Right Heschls Gyrus/Left Pallidum/Right Paracentral Lobule/Right Amygdala/Right Medial Temporal Pole/Right Cerebellum (VII)/Right Cerebellum (IX)/Right Mid Orbital Gyrus/Cerebellar Vermis (3)/Cerebellar Vermis (9)/Right Rolandic Operculum/Left Amygdala/Cerebellar Vermis (10)/Right Olfactory cortex/Left Cerebellum (III)/Left Cerebellum (X)/Right Cerebellum (III)/Cerebellar Vermis (1/2)/Left Olfactory cortex/Left Superior Orbital Gyrus/Left Rectal Gyrus | | | 89618 | 2 | | -47 | | 9 | | 10.45 | | 4.186 | |  |  |
| L | Left Postcentral Gyrus/Left Rolandic Operculum/Left Inferior Frontal Gyrus (p. Opercularis) | | | 13 | -64 | | 1 | | 15 | | 4.79 | | 0.006 | |  |  |
| R | Right Pallidum | | | 2 | 24 | | -8 | | 5 | | 4.79 | | 0.000 | |  |  |
| L | Left Inferior Frontal Gyrus (p. Triangularis) | | | 2 | -58 | | 20 | | 5 | | 4.79 | | 0.000 | |  |  |
| L | Left Inferior Frontal Gyrus (p. Opercularis) | | | 2 | -62 | | 12 | | 17 | | 4.80 | | 0.001 | |  |  |
| L | Left Precentral Gyrus/Left Postcentral Gyrus | | | 2 | -28 | | -23 | | 74 | | 4.79 | | 0.000 | |  |  |

*X, Y, and Z values are cluster center of mass coordinates in MNI stereotactic space; Voxel stats column depicts the mean and standard deviation of the voxelwise statistics for each effect.*
